# Mass drug administration of ivermectin, diethylcarbamazine, plus albendazole compared with diethylcarbamazine plus albendazole for reduction of lymphatic filariasis endemicity in Papua New Guinea: a cluster-randomised trial

**DOI:** 10.1016/S1473-3099(22)00026-3

**Published:** 2022-08

**Authors:** Moses Laman, Livingstone Tavul, Stephan Karl, Bethuel Kotty, Zebede Kerry, Stephen Kumai, Anna Samuel, Lina Lorry, Lincoln Timinao, S Cade Howard, Leo Makita, Lucy John, Sibauk Bieb, James Wangi, Jeffrey M Albert, Michael Payne, Gary J Weil, Daniel J Tisch, Catherine M Bjerum, Leanne J Robinson, Christopher L King

**Affiliations:** a Papua New Guinea Institute for Medical Research, Goroka, Papua New Guinea; b Australian Institute of Tropical Health and Medicine, James Cook University, Smithfield, QLD, Australia; c Center for Global Health and Diseases, Case Western Reserve University, Cleveland, OH, USA; d Department of Population and Quantitative Health Sciences, Case Western Reserve University, Cleveland, OH, USA; e National Department of Health, Waigani, Papua New Guinea; f WHO Papua New Guinea, NTD Program, Waigani, Papua New Guinea; g Department of Medicine, Washington University, St Louis, MO, USA; h Burnet Institute, Melbourne, VIC, Australia; i Department of Epidemiology and Preventive Medicine, Monash University, Melbourne, VIC, Australia; j Veterans Affairs Research Administration, Cleveland, OH, USA

## Abstract

**Background:**

A single co-administered dose of a triple-drug regimen (ivermectin, diethylcarbamazine, and albendazole) has been shown to be safe and more efficacious for clearing *Wuchereria bancrofti* microfilariae than the standard two-drug regimen of diethylcarbamazine plus albendazole in clinical trials. However, the effectiveness of mass drug administration with the triple-drug regimen compared with the two-drug regimen is unknown. We compared the effectiveness of mass drug administration with the triple-drug and two-drug regimens for reducing microfilariae prevalence to less than 1% and circulating filarial antigen prevalence to less than 2%, levels that are unlikely to sustain transmission of lymphatic filariasis, in Papua New Guinea.

**Methods:**

This open-label, cluster-randomised study was done in 24 villages in a district endemic for lymphatic filariasis in Papua New Guinea. Villages paired by population size were randomly assigned to receive mass drug administration with a single dose of the triple-drug oral regimen of ivermectin (200 μg per kg of bodyweight) plus diethylcarbamazine (6 mg per kg of bodyweight) plus albendazole (400 mg) or a single dose of the two-drug oral regimen of diethylcarbamazine (6 mg per kg of bodyweight) plus albendazole (400 mg). This is a follow-on study of a previously reported safety study (ClinicalTrials.govNCT02899936). All residents aged 5 years or older and non-pregnant women were asked to participate. After cross-sectional night blood microfilariae and circulating filarial antigen surveys, mass drug administration was provided at baseline and repeated 12 months later. The primary outcomes were mean prevalence of microfilariae and circulating filarial antigen at 12 months and 24 months, assessed in all residents willing to participate at each timepoint. This study is registered with ClinicalTrials.gov, NCT03352206.

**Findings:**

Between Nov 18, 2016, and May 26, 2017, 4563 individuals were enrolled in 24 clusters; 12 clusters (2382 participants) were assigned to the triple-drug regimen and 12 clusters (2181 participants) to the two-drug regimen. Mean drug ingestion rates (of residents aged ≥5 years) were 66·1% at baseline and 63·2% at 12 months in communities assigned to the triple-drug regimen and 65·9% at baseline and 54·9% at 12 months in communities assigned to the two-drug regimen. Microfilariae prevalence in the triple-drug regimen group decreased from 105 (4·4%) of 2382 participants (95% CI 3·6–5·3) at baseline to nine (0·4%) of 2319 (0·1–0·7) at 12 months and four (0·2%) of 2086 (0·1–0·5) at 24 months. In the two-drug regimen group, microfilariae prevalence decreased from 93 (4·3%) of 2181 participants (95% CI 3·5–5·2) at baseline to 29 (1·5%) of 1963 (1·0–2·1) at 12 months and eight (0·4%) of 1844 (0·2–0·9) at 24 months (adjusted estimated risk ratio 4·5, 95% CI 1·4–13·8, p=0·0087, at 12 months; 2·9, 95% CI 1·0–8·8, p=0·058, at 24 months). The prevalence of circulating filarial antigen decreased from 523 (22·0%) of 2382 participants (95% CI 20·3–23·6) at baseline to 378 (16·3%) of 2319 (14·9–17·9) at 12 months and 156 (7·5%) of 2086 (6·4–8·7) at 24 months in the triple-drug regimen group and from 489 (22·6%) of 2168 participants (20·7–24·2) at baseline to 358 (18·2%) of 1963 (16·7–20·1) at 12 months and 184 (10·0%) of 1840 (8·7–11·5) at 24 months in the two-drug regimen group; after adjustment, differences between groups were not significant.

**Interpretation:**

Mass administration of the triple-drug regimen was more effective than the two-drug regimen in reducing microfilariae prevalence in communities to less than the target level of 1%, but did not reduce circulating filarial antigen prevalence to less than 2%. These results support the use of mass drug administration with the triple-drug regimen to accelerate elimination of lymphatic filariasis.

**Funding:**

Bill & Melinda Gates Foundation.

## Introduction

Lymphatic filariasis is a parasitic infection caused by the filarial nematodes *Wuchereria bancrofti, Brugia malayi*, and *Brugia timori*. After infection, adult worms reside in the human lymphatic system and release immature forms (microfilariae) into the bloodstream; mosquito vectors then transmit the parasite via feeding activity. Infection of the lymphatic system and its ensuing dysfunction can cause recurrent swelling and disfigurement of limbs (elephantiasis), genitalia (hydrocele) in men, and breasts in women, and occasionally lymphadenitis and lymphangitis, resulting in major economic and psychosocial consequences.

In 2000, WHO launched the Global Programme to Eliminate Lymphatic Filariasis with the goal of eliminating the disease by 2020,^1^ now extended to 2030.^2^ Outside of sub-Saharan Africa, WHO recommended annual mass drug administration of diethylcarbamazine and albendazole for eligible populations for at least 5 years to reduce microfilariae prevalence such that it cannot sustain transmission, although areas with lower prevalence of lymphatic filariasis and higher coverage have achieved elimination endpoints with fewer rounds of mass drug administration.^3^ WHO recommends that in areas where lymphatic filariasis is transmitted by anopheline or culicine mosquitoes, prevalences of less than 1% microfilariae or less than 2% circulating filarial antigen (a biomarker of adult worm viability^4^) are used as possible thresholds to suggest interruption of transmission, at which point the area can enter a transmission assessment survey.^5^, ^6^ The circulating filarial antigen assessment is usually preferred, since rapid diagnostic tests are convenient to use in the field.^7^ With this approach, there has been significant progress in elimination of lymphatic filariasis in many parts of the world.^1^ Despite these successes, lymphatic filariasis persists in many countries, including Papua New Guinea, because of limited efficacy of diethylcarbamazine plus albendazole against adult filarial worms,^8^ and the logistical challenges of repeated annual treatments.

In recent randomised clinical trials, a single round of the triple-drug regimen of ivermectin, diethylcarbamazine, and albendazole was shown to be superior to the two-drug regimen of diethylcarbamazine plus albendazole for the complete suppression of microfilariae in the peripheral circulation for as long as 5 years.^8^, ^9^, ^10^, ^11^ The sustained absence of microfilariae in people who remained positive for circulating filarial antigen for up to 5 years after treatment showed that most adult worms were killed, as demonstrated by antigen testing and ultrasound, and any surviving worms are probably are sterilised,^9^, ^12^ thereby removing them from the transmission cycle. A large multicentre cluster-randomised trial has shown that the triple-drug regimen has an acceptable safety profile compared with the two-drug regimen,^13^ and 1-year follow-up studies of microfilaraemia-positive individuals from these safety studies showed the superior efficacy of the triple-drug regimen over the two-drug regimen in multiple countries.^14^, ^15^, ^16^, ^17^ As a result, WHO now recommends the triple-drug regimen for areas outside of Africa with no previous mass drug administration and in areas where transmission has not been interrupted despite multiple rounds of the two-drug regimen.^18^ The current WHO recommendation is for two annual rounds of mass drug administration with the triple-drug regimen to interrupt transmission,^18^ although the actual number of rounds required is not yet established and the effectiveness of the triple-drug regimen has not been assessed in populations. As noted, the triple-drug regimen does not kill all adult worms. Since detection of circulating filarial antigen indicates that adult worms are viable, circulating filarial antigen can persist for years after treatment, even though microfilariae have cleared.^12^ Thus, studying a community's microfilariae status after treatment with the triple-drug regimen might provide better definitive evidence of transmission interruption than circulating filarial antigen prevalence.

**Research in context**

**Evidence before this study**

In November, 2017, WHO changed its guidelines to recommend two rounds of mass drug administration of a new triple-drug combination of ivermectin, diethylcarbamazine, and albendazole for lymphatic filariasis in endemic areas outside sub-Saharan Africa that had not started mass drug administration for the disease or had not achieved interruption of transmission despite repeated rounds of mass drug administration. A previously published review by Abuelazm and colleagues in 2022 reported publications describing the efficacy of the triple-drug regimen, but none show evidence for the effectiveness of the triple-drug regimen in populations or the number of rounds of mass drug administration required to reach targets of microfilariae and circulating filarial antigen prevalence of less than 1% and less than 2%, respectively, across study sites, thresholds established by WHO to indicate interruption of transmission. We searched PubMed on Aug 3, 2021, for publications in English containing “mass drug administration”, “ivermectin”, “diethylcarbamazine”, “albendazole”, and “effectiveness” either in the title or the abstract. Our search found no articles examining the effectiveness of the triple-drug regimen in populations.

**Added value of this study**

Our findings show that one round of mass drug administration of the triple-drug regimen of ivermectin, diethylcarbamazine, and albendazole achieved a mean microfilariae prevalence of less than 1% across study villages; however, two villages with the highest baseline prevalence remained at or slightly above this threshold. Two rounds of mass drug administration resulted in microfilariae prevalence of less than 1% in all study villages. We also found that the triple-drug regimen was superior to the two-drug regimen in reaching these endpoints. Circulating filarial antigen prevalence dropped more slowly than microfilariae prevalence, and is a less suitable endpoint for assessing the effect of mass drug administration with the triple-drug regimen 24 months later.

**Implications of all the available evidence**

The findings of this study support the WHO recommendation that two rounds of mass drug administration of ivermectin, diethylcarbamazine, and albendazole can successfully reach WHO elimination targets for lymphatic filariasis, and this occurs more rapidly than with the previous two-drug regimen of diethylcarbamazine plus albendazole. Our results support the use of mass drug administration with the triple-drug regimen to accelerate elimination of lymphatic filariasis.

We did a cluster-randomised trial to assess whether one or two rounds of mass drug administration of the triple-drug regimen can reduce microfilariae prevalence to less than 1% and circulating filarial antigen prevalence to less than 2% compared with standard treatment with the two-drug regimen in an area of Papua New Guinea that had not previously received mass treatment for lymphatic filariasis.

## Methods

### Study design and participants

**Figure 1:**
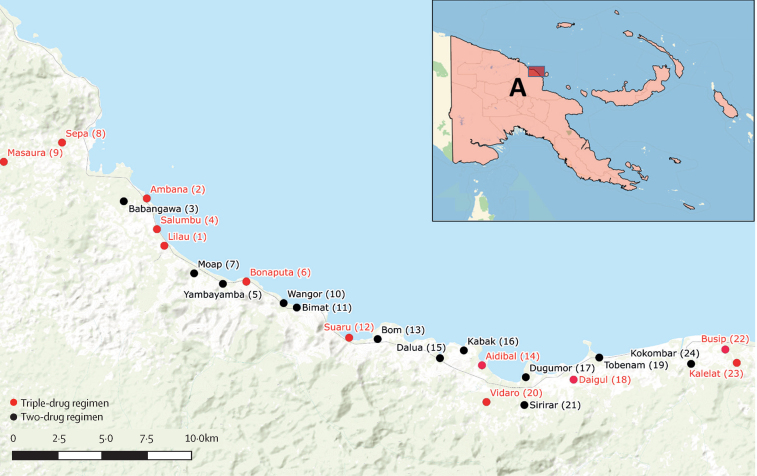
Study map Insert shows location of study site in Papua New Guinea. Numbers in brackets refer to village code numbers (as shown in appendix pp 2–3). Map previously published in Tavul et al (2022).^17^

This study was a parallel-arm, cluster-randomised controlled trial done in 24 villages in the Bogia District, Madang Province, Papua New Guinea, between Nov 18, 2016, and Aug 2, 2019. The villages are scattered along the northern coast of the Bismarck Sea (figure 1). The primary economic activities are subsistence farming and fishing. Study communities were selected because they had no previous community-based treatment for lymphatic filariasis and previous unpublished surveys and recent convenience sampling had shown the presence of lymphatic filariasis. Long-lasting insecticide-treated nets are distributed as part of the malaria control programme throughout Papua New Guinea and were distributed in the study area in 2014 and 2017. Of note, long-lasting insecticide-treated nets distributed between 2013 and 2019 had markedly reduced bioefficacy for killing mosquitoes.^19^

Participants were initially registered as part of a multicentre, multicountry clinical trial of triple-drug treatment (ivermectin with diethylcarbamazine and albendazole) versus a two-drug combination (diethylcarbamazine plus albendazole) that assessed safety as the primary outcome (ClinicalTrials.gov NCT02899936).^13^ A relevant secondary outcome of the parent study was the efficacy in individuals of the triple-drug regimen versus the two-drug regimen 12 months after treatment (see safety protocol, appendix pp 6–95), which will be reported elsewhere. Using the sample size from the parent study, a new protocol was written to examine the effectiveness of the triple-drug regimen versus the two-drug regimen in communities. The primary outcome in this new protocol was the effect of the initial treatment and a second round of mass drug administration at 12 months on community prevalence of microfilariae and circulating filarial antigen at 12 months and 24 months in Papua New Guinea (ClinicalTrials.gov NCT03352206; for effectiveness protocol see appendix pp 96–130). Other secondary outcomes from the safety protocol were included in the effectiveness protocol: community acceptance of mass drug administration with the triple-drug regimen versus the two-drug regimen, and the effect of the regimens on transmission of lymphatic filariasis. Secondary outcomes from the effectiveness protocol, described below, will also be reported elsewhere.

Clusters were villages representing distinct political and cultural units in Papua New Guinea with locally recognised borders. All eligible and consenting people in each community were offered a single co-administered dose of either the triple-drug regimen or the two-drug regimen immediately following enrolment for the safety study and once again 12 months later after re-enrolment for the effectiveness trial. Before treatment, all individuals were tested for the presence of circulating filarial antigen and if positive were then tested for the presence of microfilariae at baseline and at 12 months. Participants were tested again for microfilariae at 24 months, although they were not treated at this time.

A census of all members in all households in each community was done at baseline and social mobilisation activities to support mass drug administration were initiated before study enrolment. Inclusion criteria were all eligible residents in study villages. Exclusion criteria were children younger than 5 years, weight less than 15 kg, pregnancy (or last menstrual period >4 weeks ago or unknown), breastfeeding within 7 days of delivery, acute or chronic illness severe enough to interfere with activities of daily living, or any history or previous allergy to the study drugs. Age-specific treatment rates were calculated based on the total population in the census aged 5 years or older, including those ineligible for mass drug administration (e.g., pregnant women, chronic illness). Overall treatment coverage rate excluded children younger than 5 years who were ineligible for mass drug administration.

Institutional review boards at University Hospitals Cleveland Medical Center, the Papua New Guinea Institute of Medical Research, and the Papua New Guinea Medical Research Advisory Committee approved the trial. All participants in each village provided written informed consent. Participation of minors required their assent and written consent from at least one parent or guardian.

### Randomisation and masking

Randomisation was performed at the village level. Study statisticians identified pairs of villages with similar population sizes and lymphatic filariasis infection rates on the basis of limited convenience sampling. Study statisticians used a SAS random number generator to assign the villages in each pair to treatment with the triple-drug regimen or the two-drug regimen. Randomisation was done before enrolment so that community members and investigators administering drugs knew treatment allocation. Staff reading the slides for microfilariae (distant from study sites) were masked to treatment allocation by use of identification numbers that could not be linked to the village without a key held by the study statisticians. Measurement of circulating filarial antigen was not masked because it was done at the time of blood collection.

### Procedures

Fingerstick blood samples were collected at the time of the surveys for circulating filarial antigen testing with Filariasis Test Strips (FTS; Alere, Scarborough, ME, USA) according to the manufacturer's protocol. Test results were scored as previously described,^20^ based on the intensity of the test line. Test scores were recorded as follows: 0, no test line visible (negative test); 1, the test line is present but weaker than the procedural control line; 2, the test line is equal in intensity to the control line; or 3, the test line is stronger than the control line. Tests with no control line were considered invalid and repeated. The FTS detect a biomarker for infection with *W bancrofti* adult worms and have high sensitivity for detecting people with microfilaraemia. In clinical trials, we found positivity for FTS and microfilariae before and after treatment to be 100% concordant.^8^, ^9^ In larger community studies, FTS also detected 100% of microfilaraemia-positive individuals.^21^ People with positive FTS results were tested for microfilaraemia by use of 60 μL thick blood smears prepared from fingerstick samples collected between 2100 h and 0100 h, as previously described.^21^

Treatment included a single dose of the three-drug oral regimen of ivermectin (200 μg per kg of bodyweight) plus diethylcarbamazine (6 mg per kg of bodyweight) plus albendazole (400 mg) or a single dose of the two-drug oral regimen of diethylcarbamazine (6 mg per kg of bodyweight) plus albendazole (400 mg). Project staff directly observed ingestion of study medications by participants. Participants were activity monitored for adverse events 24 and 48 hours after treatment and passively up to day 7, as previously described.^13^

### Outcomes

The primary objective was to determine whether clusters assigned to the triple-drug regimen were more likely to achieve a mean microfilariae prevalence of less than 1% or circulating filarial antigen prevalence of less than 2%, or both, compared with the two-drug regimen 12 months and 24 months after one and two rounds of mass drug administration. The primary outcomes were mean prevalence of microfilariae and circulating filarial antigen at 12 months and 24 months. As secondary outcomes, the protocol lists the effect of mass drug administration with the triple-drug regimen or the two-drug regimen on the proportion of mosquitoes infected with *W bancrofti* DNA (molecular xenomonitoring), acquisition of antifilarial antibodies, effect on soil-transmitted helminths, and community acceptability of the triple-drug and two-drug regimens, which will be reported elsewhere. Of note, the primary outcome in the clinical trial registration incorrectly included antifilarial antibodies as a primary outcome, which it is not per protocol.

### Statistical analysis

The sample size for this clinical trial was determined in the parent study as part of a multicentre safety study, as previously described.^13^ The primary objective was to test whether microfilariae prevalence could reach less than 1% in clusters assigned to the triple-drug regimen compared with clusters assigned to the two-drug regimen. In the original safety protocol, the required sample size was estimated to be 1000 individuals in each treatment group on the basis of a target of reaching a true microfilariae prevalence of 0·5% with an upper 95% CI of less than 1%. A formal power calculation was not included in the secondary effectiveness protocol; however, we have provided this calculation here based on a priori information. We expect the triple-drug regimen at 24 months to provide a 0·2% prevalence (on the basis of an assumed baseline microfilariae prevalence of 5% and assuming 96% clearance with treatment).^8^ For the two treatment groups, we assumed a dropout rate by 24 months of 15%, approximately equal sample sizes, approximately equal village sizes, equal coverage, and a within-cluster (village) correlation of 0·01. For the two-drug regimen, previous data suggest a clearance rate of 56% at 24 months, which would yield an expected 2·2% microfilariae prevalence. Using a two-sided (0·05 alpha level) Z test with pooled variance, we calculated that there is more than 99% power to detect the above difference in microfilariae prevalence rates. Further, this sample size provides 81% power for the test of a within-treatment group microfilariae prevalence of less than 1% (based on a one-sided 0·05 alpha-level binomial test, under an actual 0·2% prevalence—as expected for treatment with the triple-drug regimen—and other assumptions previously stated). Similarly, there is 85% power for the test of within-treatment group circulating filarial antigen prevalence of less than 2% (assuming an actual 0·8% prevalence).

Individuals included in the analysis were all village members willing to participate and who met inclusion criteria for receiving mass drug administration at each sampling timepoint.

Statistical analyses were done with SPSS version 25 and SAS 9.4. A generalised estimating equations approach (using the GENMOD procedure in SAS) was used to estimate microfilariae or circulating filarial antigen prevalence (for each treatment group and time) while accounting for clustering within villages. Each model (which included only an intercept term) used the identity link function and exchangeable working correlation; corresponding robust 95% CIs for each prevalence (and p values for the test of prevalence equal to 0·01) were computed. A generalised estimating equations approach was also used to assess differences in proportions (separately for the microfilariae positive and circulating filarial antigen positive [binary] outcomes, and for the 12-month and 24-month follow-up times) between the two treatment groups adjusting for baseline predictors. Specifically, a modified Poisson regression (logit link) model was used with an exchangeable working correlation structure with villages as clusters using individual level data.^22^ This approach allows for the estimation of treatment effects interpreted as risk ratios. Aside from the binary (village-assigned) treatment indicator, the model covariates included the individual's age, sex, and use of bed net, and the percentage of people treated in the individual's village (coverage). A (robust) Wald test of the treatment effect was performed and the corresponding estimated risk ratio (and 95% CI) obtained (for each follow-up time and outcome). Estimated effects were considered statistically significant if the p value was less than 0·05. This study is registered with ClinicalTrials.gov, NCT03352206.

### Role of the funding source

The funder of the study contributed to the study concept, but had no role in study design, data collection, data analysis, data interpretation, or writing of the report.

## Results

**Figure 2:**
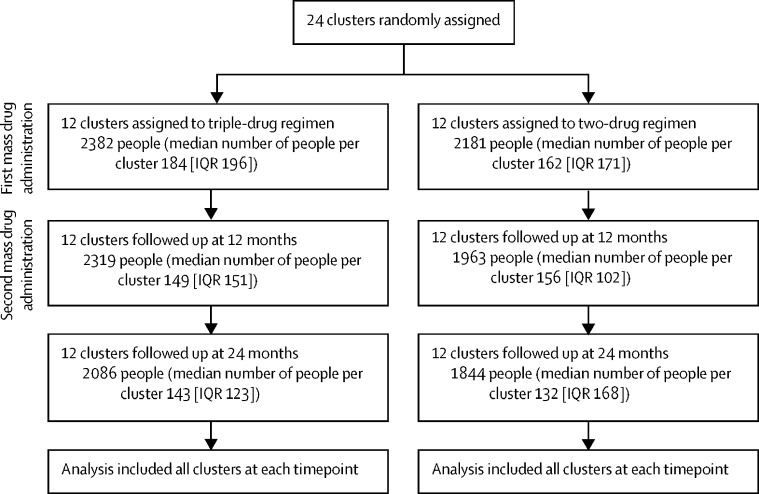
Trial profile

Participants were enrolled from Nov 18, 2016, until May 26, 2017, in 24 communities in Papua New Guinea (figure 2). Populations in the study villages and the number of people sampled are shown in the table and appendix (pp 2–3). At baseline, 4563 individuals participated in the trial, with 2382 in the triple-drug regimen group (12 villages) and 2181 in the two-drug regimen group (12 villages). This was a serial cross-sectional study and people were asked to give consent at each timepoint. The reduced number of individuals at each timepoint represents fewer individuals willing to participate.

**Table:** Characteristics of the study population stratified by treatment group

	Population for all clusters (range of population for each cluster)*	**Number of enrolled participants**	**Number of male participants (%)**	**Number of female participants (%)**	**Median age of participants (IQR)**	**Number of participants FTS positive (%; range**†**)**	**Number of participants microfilariae positive (%; range**†**)**
**Triple-drug regimen (12 clusters)**							
Baseline	3682 (146–584)	2382	1286 (54·0%)	1096 (46·0%)	23 (12–45)	523 (22·0%; 7·7–39·5%)	105 (4·4%; 0·0–17·6%)
12 months	NA	2319	1175 (50·7%)	1144 (49·3%)	20 (11–43)	378 (16·3%; 3·3–32·6%)	9 (0·4%; 0·0–1·5%)
24 months	NA	2086	1067 (51·2%)	1019 (48·8%)	18 (10–44)	156 (7·5%; 1·5–18·5%)	4 (0·2%; 0·0–0·6%)
**Two-drug regimen (12 clusters)**							
Baseline	3326 (132–755)	2181	1160 (53·2%)	1021 (46·8%)	21 (11–46)	489 (22·4%; 1·2–43·4%)	93 (4·3%; 0·0–13·1%)
12 months	NA	1963	1023 (52·1%)	940 (47·9%)	20 (10–43)	358 (18·2%; 1·9–33·8%)	29 (1·5%; 0·0–2·6%)
24 months	NA	1844	1002 (54·3%)	842 (45·7%)	19 (11–41)	184 (10·0%; 1·3–30·8%)	8 (0·4%; 0·0–1·4%)

FTS=Filariasis Test Strips (correlate with circulating filarial antigen concentrations). NA=not applicable.

* The population in the 12 communities after the baseline census (range of population in the 12 clusters) aged 5 years or older.

† Proportion of enrolled participants with positive test (range of proportion across the 12 clusters).

Mean household size was six individuals (range 1–13) with an average of 2·3 adults. 4128 (50·0%) of 8253 individuals in the total population in the study area were aged 18 years or older. The proportions of male and female participants, median age of participants (table), and proportion of individuals using long-lasting insecticide-treated nets (net use: triple-drug regimen group, mean 85·8%, range 79·3–98·9%; two-drug regimen group, mean 82·1%, range 76·9–100%) were similar between treatment groups. The frequency and intensity of lymphatic filariasis infection as determined by mean percent circulating filarial antigen positivity (triple-drug regimen, 523 [22·0%] of 2382 participants; two-drug regimen, 489 [22·4%] of 2181 participants; table) and proportion of individuals with weak (1+), medium (2+), and high (3+) semi-quantitative circulating filarial antigen scores were similar at baseline (151 [28·9%] of 523 participants, 199 [38·0%], and 174 [33·3%] in the triple-drug regimen group; 140 [28·6%] of 489 participants, 182 [37·2%], and 167 [34·2%] in the two-drug regimen group, respectively; appendix pp 2–3). The mean percent microfilariae positivity by treatment group was also similar (105 [4·4%] of 2382 participants in the triple-drug regimen group; 93 [4·3%] of 2181 participants in the two-drug regimen group; table). Community mass drug administration ingestion rates (based on the total population aged ≥5 years) were 2432 (66·1%) of 3682 participants at baseline and 2319 (63·2%) of 3671 at 12 months in triple-drug regimen communities and 2192 (65·9%) of 3326 at baseline and 2061 (54·9%) of 3757 at 12 months in the two-drug regimen communities. Treatment coverage varied with age: drugs were ingested by a mean 35·1% of children aged 5–9 years in the triple-drug regimen group compared with 40·2% in the two-drug regimen group, by 72·7% of adolescents (aged 10–17 years) in the triple-drug regimen group compared with 75·7% in the two-drug regimen group, and by 71·8% of adults (aged 18 years or older) in the triple-drug regimen group compared with 69·8% in the two-drug regimen group (appendix p 4–5).

1 year after the first round of mass drug administration, prevalence of microfilariae in the 12 villages assigned to the triple-drug regimen decreased from 105 (4·4%) of 2382 participants (95% CI 3·6–5·3) at baseline to nine (0·4%) of 2319 (95% CI 0·1–0·7). 1 year after the second round of mass drug administration, microfilariae prevalence further decreased to four (0·2%) of 2086 participants (95% CI 0·1–0·5). Using a generalised estimating equations approach, a test for microfilariae prevalence less than 1% within villages assigned to the triple-drug regimen at 12 months was significant (p=0·00031; table, appendix p 2). By contrast, the prevalence of microfilariae in the 12 villages assigned to the two-drug regimen decreased from 93 (4·3%) of 2181 participants (95% CI 3·5–5·2) at baseline to 29 (1·5%) of 1963 (95% CI 1·0–2·1; p=0·32) at 12 months, and to eight (0·4%) of 1844 (95% CI 0·2–0·9) at 24 months (p=0·00073). Thus, the microfilariae prevalence was significantly less than 1% for the triple-drug regimen group at both 12 months and 24 months, and for the two-drug regimen group at 24 months.

**Figure 3:**
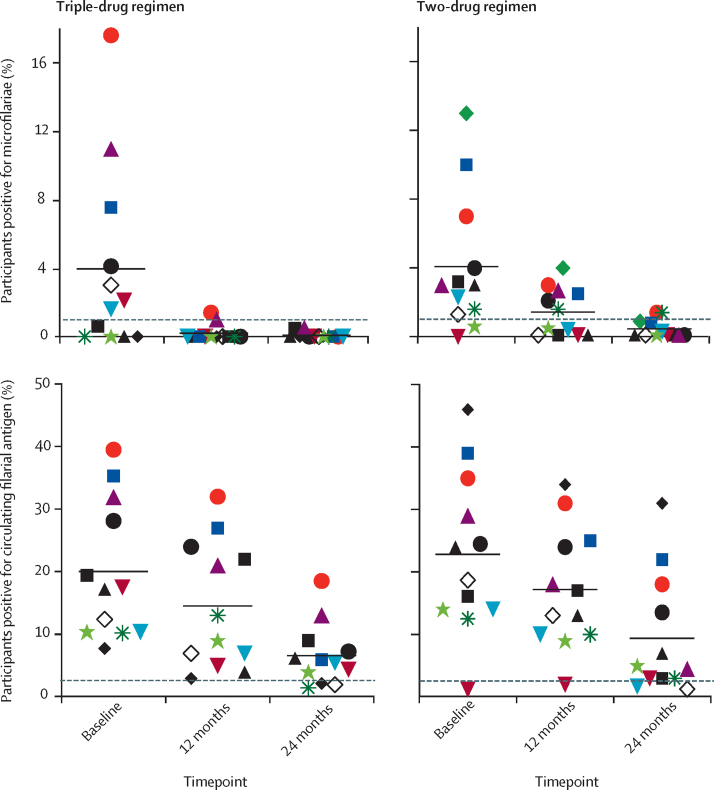
Prevalence of microfilariae and circulating filarial antigen at baseline, 12 months, and 24 months after initial mass drug administration overall and in each village Each symbol represents a separate village at different timepoints. Horizontal lines indicate overall mean prevalence. There were 12 villages in the triple-drug regimen group and 12 villages in the two-drug regimen group. Dashed lines indicate the 1% microfilariae prevalence and 2% circulating filarial antigen prevalence targets recommended by WHO.

We examined the more stringent criterion of whether mass drug administration could achieve microfilariae prevalence of less than 1% in all study villages after one and two rounds of treatment. In two of 12 villages in the triple-drug regimen group, prevalence was 1·5% (95% CI 0·2–5·3) and 1·0% (95% CI 0·4–2·3) 12 months after one round of mass drug administration; these two villages had the highest microfilariae prevalence at baseline (17·2% and 10·5%, respectively; figure 3). After the second round of mass drug administration, all 12 study villages assigned to the triple-drug regimen had microfilariae prevalence of less than 1% at 24 months (figure 3, appendix pp 2–3). By contrast, microfilariae prevalence in the two-drug regimen group remained higher than the 1% target in six villages at 12 months (range 1·6–4·0%) and in two villages at 24 months (ie, after two rounds of mass drug administration; 1·4% and 1·4%).

**Figure 4:**
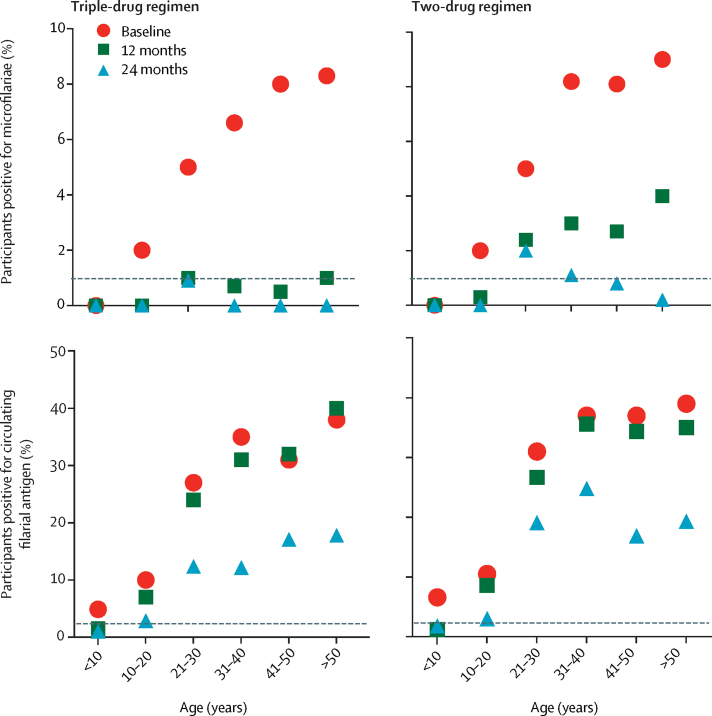
Prevalence of microfilariae and circulating filarial antigen at baseline, 12 months, and 24 months after the first round of mass drug administration by age group Dashed lines indicate the 1% microfilariae prevalence and 2% circulating filarial antigen prevalence targets recommended by WHO.

To assess differences between treatment groups, we used a generalised estimating equations Poisson regression model controlling for clustering, village-level coverage of mass drug administration, individual bednet use, age, and sex. The estimated risk ratio for the difference between treatment groups in reduction of microfilariae prevalence was 4·5 (95% CI 1·4–13·8) at 12 months (p=0·0087) and 2·9 (95% CI 1·0–8·8, p=0·058) at 24 months. Neither village treatment coverage nor bednet use were significant predictors of decreases in microfilariae prevalence after the first round of mass drug administration (p=0·23 and p=0·69, respectively) or at 24 months (p=0·32 and p=0·17, respectively). The greater reduction in microfilariae prevalence in the triple-drug regimen group relative to the two-drug regimen group was seen in all age groups (figure 4).

The prevalence of circulating filarial antigen in the 12 villages assigned to the triple-drug regimen decreased from 523 (22·0%) of 2382 participants (95% CI 20·3–23·6) at baseline to 378 (16·3%) of 2319 (95% CI 14·9–17·9) at 12 months, and to 156 (7·5%) of 2086 (95% CI 6·4–8·7) at 24 months. In the 12 villages assigned to the two-drug regimen, the prevalence of circulating filarial antigen decreased from 489 (22·4%) of 2181 participants (95% CI 20·7–24·2) at baseline to 358 (18·2%) of 1963 (95% CI 16·7–20·1) at 12 months, and to 184 (10·0%) of 1844 (95% CI 8·7–11·5) at 24 months (table, figure 3). Thus, there was no evidence that the targeted circulating filarial antigen prevalence of less than 2% was reached for either regimen at 12 months or 24 months. The reduction in circulating filarial antigen prevalence did not differ significantly between the triple-drug regimen and two-drug regimen groups at 12 and 24 months as shown by a generalised estimating equations Poisson regression model controlling for clustering, mass drug administration coverage, individual bednet use, age, and sex (estimated risk ratio 1·1, 95% CI 0·7–1·5, p=0·81, at 12 months; and 1·8, 0·9–3·4, p=0·087, at 24 months).

**Figure 5:**
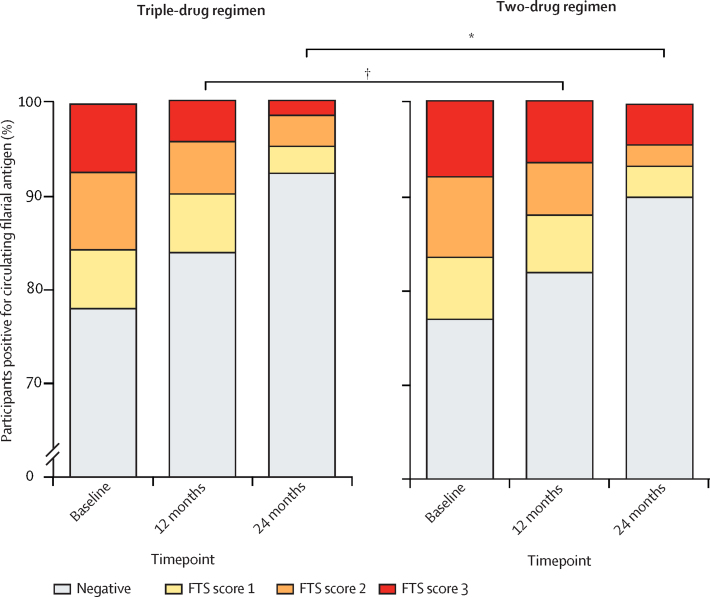
Changes in FTS scores (which correlate with circulating filarial antigen concentrations) by treatment group, before and after mass drug administration The FTS scores (FTS 0–3) did not differ significantly between the treatment groups at baseline (χ^2^=0·28, p=0·96), but did differ significantly after mass drug administration at 12 months (χ^2^=9·42, p=0·024) and 24 months (χ^2^=35·60, p<0·0001). In FTS-positive individuals only, participants in the triple-drug regimen group were significantly less likely than participants in the two-drug regimen group to have the highest FTS scores (FTS 3 *vs* FTS 1 or 2) at 12 months (χ^2^=6·56, p=0·010) and 24 months (χ^2^=26·13, p<0·0001), but not at baseline (χ^2^=0·101, p=0·750). FTS scores: 0, no test line visible (negative test); 1, the test line is present but weaker than the procedural control line (weak); 2, the test line is equal in intensity to the control line (medium); or 3, the test line is stronger than the control line (high). FTS= Filariasis Test Strips. *p<0·0001. †p=0·024.

Circulating filarial antigen clearance following treatment occurred across age groups, with a slightly greater effect in the triple-drug regimen group than in the two-drug regimen group in participants aged 21–40 years (figure 4). Circulating filarial antigen scores decreased to a greater extent in the triple-drug regimen group compared with the two-drug regimen group (figure 5). The FTS scores (FTS 0–3) did not differ significantly between the triple-drug regimen and the two-drug regimen groups at baseline, but did differ significantly after mass drug administration at 12 months and 24 months. Furthermore, among FTS-positive individuals only, participants in the triple-drug regimen group were significantly less likely than participants in the two-drug regimen group to have the highest FTS scores (FTS 3 *vs* FTS 1 or 2) at 12 months and 24 months, but not at baseline.

Adverse events in study groups following the first round of treatment are reported elsewhere.^13^

## Discussion

This study found that a single round of mass drug administration with a triple-drug regimen of ivermectin, diethylcarbamazine, and albendazole reduced community prevalence of microfilariae by ten times; this reduction was 4·5 times greater than that seen in communities assigned to the two-drug regimen of diethylcarbamazine and albendazole. To our knowledge, this study is the first to show the superior effectiveness of the triple-drug regimen versus the two-drug regimen at the community level. Although the difference between groups in microfilariae prevalence was less pronounced after two rounds of mass drug administration, two of 12 villages assigned to the two-drug regimen had residual prevalence greater than 1% after two rounds compared with none of 12 villages assigned to the triple-drug regimen.

The presence of circulating filarial antigen is a sensitive biomarker for the presence and number of viable adult *W bancrofti* worms.^4^ Prevalence of circulating filarial antigen decreased less substantially than microfilariae prevalence after the first round of mass drug administration without a significant difference between treatment groups. However, prevalence of circulating filarial antigen fell sharply after the second round of mass drug administration with an overall greater decrease in prevalence in the triple-drug regimen group than in the two-drug regimen group at 24 months. These results, together with reductions in FTS scores after mass drug administration, are consistent with the known partial macrofilaricidal effects of these treatments. The effect of mass drug administration with the triple-drug regimen was greater on clearance of microfilariae than on clearance of circulating filarial antigen. This finding is consistent with results from previous clinical trials suggesting that the triple-drug regimen has a strong sterilising effect on adult worms in addition to its partial macrofilaricidal effect.^9^, ^23^ Prevalence of circulating filarial antigen remained above the 2% WHO pre-transmission threshold in most villages after two rounds of mass drug administration in both treatment groups. Thus, circulating filarial antigen monitoring underestimated the effect of mass drug administration on the potential for continuing transmission relative to microfilariae results.

Several limitations of the study should be highlighted. Poor compliance with mass drug administration was seen in children younger than 10 years. However, because few young children were microfilaraemic, they were not important sources of microfilariae for transmission of lymphatic filariasis in this area. Reduced compliance in adults in the second round of mass drug administration might have contributed to the reduced efficacy of mass drug administration in villages assigned to the two-drug regimen. Because of the higher effectiveness of the triple-drug regimen, most microfilariae-positive individuals had completely cleared by 12 months and the reduced coverage at 12 months would have had less effect on community microfilariae burden in villages treated with the triple-drug regimen. Another limitation is that the village residents can change over the period of observation, with people moving in from other areas not receiving mass drug administration and others moving away, which could affect estimates of microfilariae rates in the communities.

Several factors should be considered regarding the generalisability of our results. First, lymphatic filariasis is transmitted in Papua New Guinea by anopheline mosquitoes, which are less competent vectors than culicine or *Aedes* mosquitoes.^24^ More rounds of mass drug administration with the triple-drug regimen might be required to achieve elimination of lymphatic filariasis in areas with transmission by *Culex* or *Aedes* mosquitoes. Second, the efficacy of the triple-drug regimen might vary in different regions. The efficacy of a single dose of the triple-drug regimen for clearing microfilariae at 1-year post treatment has ranged from 94% to 97% in Papua New Guinea and Haiti, 84% in India, 78% in Côte d'Ivoire, and 63% in Fiji.^8^, ^9^, ^14^, ^16^, ^25^ These differences might be caused by variable susceptibility of adult worms or differences in absorption and metabolism of drugs, although factors such as compliance with swallowing the drugs and reinfection might also account for variability in measured efficacy. Third, results obtained in this small research project where mass drug administration was distributed by dedicated teams with high compliance might not be replicated by national programmes that treat hundreds of thousands or millions of people. Fourth, this study also examined whether mass drug administration reached the target of less than 1% microfilariae prevalence in each cluster, a criterion that might be considered to provide greater confidence that transmission of lymphatic filariasis has been interrupted. This approach contrasts with the current recommendation of reaching a mean microfilariae prevalence of less than 1% across sample clusters throughout an implementation unit, which could discount areas with poor coverage or high initial baseline infection rates.

Our findings have important implications for countries that are considering the use of the triple-drug regimen for elimination of lymphatic filariasis. The consistently high efficacy of the triple-drug regimen seen in this study and in other areas of Papua New Guinea^8^, ^26^ suggests that elimination of lymphatic filariasis is a feasible goal for this country, which has the highest burden of lymphatic filariasis in the South Pacific.^27^ Coverage and compliance are key for any mass drug administration programme, and the superior treatment regimen will not fix poor compliance. One or two rounds of the triple-drug regimen with high compliance might be sufficient to interrupt transmission of lymphatic filariasis in some settings. Our study also showed that prevalence of circulating filarial antigen was not a sensitive parameter for assessing the risk for continuing transmission of lymphatic filariasis after two rounds of mass drug administration of the triple-drug regimen. Thus, pending development of new tools, assessment of the effect of mass drug administration with the triple-drug regimen should focus on detection of microfilariae, which can be facilitated by prescreening for circulating filarial antigen and restricting microfilariae testing to those with positive antigen tests.

## Data sharing

The dataset with deidentified participant information described in the current paper and the study protocol will be made available upon request to the corresponding author.

## Declaration of interests

We declare no competing interests.
