# Supplementary material for: Mass drug administration of ivermectin, diethylcarbamazine, plus albendazole compared with diethylcarbamazine plus albendazole for reduction of lymphatic filariasis endemicity in Papua New Guinea: a cluster-randomised trial
Source: Lancet Infect Dis. 2022 Aug;22(8):1200–9. doi: 10.1016/S1473-3099(22)00026-3 (PMC9300473; doi:10.1016/S1473-3099(22)00026-3)
Supplement: Supplementary appendix [file mmc1.pdf]

THE LANCET
Infectious Diseases

Supplementary appendix

This appendix formed part of the original submission and has been peer reviewed. We post it as supplied by the authors.

Supplement to: Laman M, Tavul L, Karl S, et al. Mass drug administration of ivermectin, diethylcarbamazine, plus albendazole compared with diethylcarbamazine plus albendazole for reduction of lymphatic filariasis endemicity in Papua New Guinea: a cluster-randomised trial. *Lancet Infect Dis* 2022; published online May 6. https://doi.org/10.1016/S1473-3099(22)00026-3.

Table of content for the appendix

Table 1. Individual clusters (villages) stratified by treatment group with median age, sex, and lymphatic filariasis infection parameters
……………………………………………………………………………..2

Table 2. Individual cluster census and drug coverage rates at baseline and 12 months stratified by treatment group age and sex
……………………………………………………………………………4

Safety protocol (90 pages)
……………………………………………………………………………………………6-95

Effectiveness protocol (35 pages)
………………………………………………………………………………..96-130

BASELINE											
MDA Treatment	Village #	# enrolled	# of males	Median age	% FTS +	% FTS+ Score 1 (Low)	% FTS+ Score 2 (Medium)	% FTS+ Score 3 (High)	% MF+	95% CI
IDA	1	187	94 (50%)	23.5	28.1% (53/187)	15% (8/53)	36% (19/53)	49% (26/53)	3.2 % (6/187)	(0.7-5.7%)
IDA	2	301	154 (51%)	23	16.9% (51/301)	35% (18/51)	27% (14/51)	37% (19/51)	0.7% (2/301)	(0.0-1.6%)
IDA	4	63	40 (64%)	20	19.0% (12/63)	58% (7/12)	42% (5/12)	0% (0/12)	0% (0/63)	
IDA	6	144	81 (56%)	20	17.9% (27/144)	55% (15/27)	33% (9/27)	11% (3/27)	1.4% (2/144)	(0.0-3.3%)
IDA	8	322	171 (53%)	20	7.7% (25/322)	52% (13/25)	40% (10/25)	8% (2/25)	0% (0/322)	
IDA	10	119	61 (51%)	23	39.5% (47/119)	8% (4/47)	38% (18/47)	53% (25/47)	17.2% (20/116)	(10.8-24.5%)
IDA	12	290	144 (49%)	23	37.0% (107/290)	20% (21/107)	38% (41/107)	42% (45/107)	7.6% (22/290)	(4.8-11.3%)
IDA	14	404	233 (57%)	25	33.9% (137/404)	23% (31/137)	40% (55/137)	37% (51/137)	10.5% (42/399)	(8.0-14.3%)
IDA	18	181	96 (53%)	24	10.4% (19/181)	68% (13/19)	21% (4/19)	10% (2/19)	1.7% (3/181)	(0.3-4.8%)
IDA	20	217	108 (49%)	23	12.4% (28/217)	46% (13/28)	54% (15/28)	0% (0/28)	2.8% (6/217)	(1.0-5.0%)
IDA	22	58	29 (50%)	22	13.0% (7/58)	71% (5/7)	28% (2/7)	0% (0/7)	0% (0/58)	
IDA	23	96	64 (66%)	19	10.4% (10/96)	20% (2/10)	70% (7/10)	10% (1/10)	0% (0/96)	
TOTALS	**Totals**	**2382**	**1286 (53%)**	**23**	**22% (523/2382)**	**29% (151/523)**	**38% (199/523)**	**33% (174/523)**	**4.4% (105/2367)**	**3.6-5.3%**

12 months

MDA Treatment	Village	# enrolled	# of males	Median age	% FTS +	% FTS+ Score 1 (Weak)	% FTS+ Score 2 (Medium)	% FTS+ Score 3 (Strong)	% MF+	95% CI
IDA	1	163	88 (54%)	20	24% (39/163)	31% (12/39)	51% (20/39)	18% (7/39)	0% (0/163)	
IDA	2	197	98 (50%)	21.5	22% (43/197)	53% (23/43)	34% (15/43)	12% (5/43)	0% (0/197)	
IDA	4	104	51 (49%)	18	4% (4/104)	50% (2/4)	50% (2/4)	0% (0/4)	0% (0/104)	
IDA	6	86	52 (60%)	19	5% (4/86)	75% (3/4)	25% (1/4)	0% (0/4)	0% (0/86)	
IDA	8	299	153 (51%)	18.5	3% (10/299)	80% (8/10)	20% (2/10)	0% (0/10)	0% (0/299)	
IDA	10	136	61 (45%)	20	32% (44/136)	23% (10/44)	23% (10/44)	54% (24/44)	1.5% (2/136)	(0.2-5.3%)
IDA	12	267	127 (48%)	18	27% (71/267)	24% (17/71)	42% (30/71)	34% (24/71)	0% (0/267)	
IDA	14	572	303 (53%)	21	21% (121/572)	37% (45/121)	33% (40/121)	30% (36/121)	1.0% (6/572)	(0.4-2.3%)
IDA	18	132	67 (51%)	23	7% (10/132)	40% (4/10)	30% (3/10)	30% (3/10)	0.8% (1/132)	(0.0-4.3%)
IDA	20	172	81 (47%)	19.5	7% (13/172)	54% (7/13)	38% (5/13)	8% (1/13)	0% (0/172)	
IDA	22	97	41 (43%)	22	13% (11/97)	91% (10/11)	0% (0/11)	9% (1/11)	0% (0/97)	
IDA	23	94	53 (57%)	19	9% (8/94)	37% (3/8)	25% (2/8)	37% (3/8)	0% (0/94)	
TOTALS	**Totals**	**2319**	**1175 (51%)**	**20**	**16% (378/2319)**	**38% (144/378)**	**34% (130/378)**	**28% (104/378)**	**0.4% (9/2319)**	**(0.1-0.7%)**

24 months

MDA Treatment	Village	# enrolled	# of males	Median age	% FTS +	% FTS+ Score 1 (Weak)	% FTS+ Score 2 (Medium)	% FTS+ Score 3 (Strong)	% MF+	95% CI
IDA	1	151	78 (52%)	17	7.3% (11/151)	64% (7/11)	27% (3/11)	9% (1/11)	0% (0/151)	
IDA	2	181	86 (47%)	19	9% (16/181)	37% (6/16)	44% (7/16)	19% (3/16)	0.6% (1/181)	(0.0-3.0%)
IDA	4	96	57 (59%)	18	6.2% (6/96)	50% (3/6)	17% (1/6)	33% (2/6)	0% (0/96)	
IDA	6	90	58 (64%)	21.5	4.4% (4/90)	75% (3/4)	25% (1/4)	0% (0/4)	0% (0/90)	
IDA	8	228	111 (49%)	17	2.2% (5/228)	60% (3/5)	40% (2/5)	0% (0/5)	0% (0/228)	
IDA	10	97	45 (46%)	18	18.5% (18/97)	28.0% (5/18)	44% (8/18)	28% (5/18)	0% (0/97)	
IDA	12	198	80 (40%)	17	6% (12/198)	33.3% (4/12)	42% (5/12)	25% (3/12)	0% (0/198)	
IDA	14	467	243 (51.8%)	19	13% (63/467)	32% (20/63)	51% (32/63)	17% (11/63)	0.6% (3/467)	(0.1-1.8%)
IDA	18	236	129 (55%)	19	5.5% (13/236)	31% (4/13)	61% (8/13)	8% (1/13)	0% (0/236)	
IDA	20	108	52 (48%)	20	2% (2/108)	100% (2/2)	0% (0/2)	0% (0/0)	0% (0/108)	
IDA	22	134	71 (53%)	17	1.5% (2/134)	50% (1/2)	50% (1/2)	0% (0/2)	0% (0/134)	
IDA	23	100	58 (58%)	19	4% (4/100)	50% (2/4)	0% (0/4)	50% (2/4)	0% (0/100)	
TOTALS	**Totals**	**2086**	**1067 (51%)**	**18**	**7.5% (156/2086)**	**38% (60/156)**	**43% (68/156)**	**18% (28/156)**	**0.2% (4/2086)**	**(0.1-0.5%)**

BASELINE

MDA Treatment	Village	# enrolled	# of males	Median age	% FTS +	% FTS+ Score 1 (Weak)	% FTS+ Score 2 (Medium)	% FTS+ Score 3 (Strong)	% MF+	(95% CI)
DA	3	125	76 (60%)	19	20.0% (25/125)	20% (5/25)	20% (5/25)	60% (15/25)	4% (5/125)	(1.3-9.1%)
DA	5	62	37 (60%)	20	14.5% (9/62)	22% (2/9)	78% (7/9)	0% (0/9)	3.2% (2/62)	(0.4-11.2%)
DA	7	100	52 (52%)	26	24% (24/100)	33% (8/24)	33% (8/24)	33% (8/24)	3% (3/100)	(0.6-5.2%)
DA	9	82	34 (41%)	23	1.2% (1/82)	0% (0/1)	100% (1/1)	0% (0/1)	0% (0/82)	
DA	11	145	80 (55%)	24.5	43% (63/145)	11% (7/64)	28% (18/64)	59% (38/64)	13% (19/145)	(8.1-19.7%)
DA	13	215	112 (52%)	20	35% (76/215)	25% (19/76)	59% (45/76)	16% (12/76)	7.0% (15/215)	(4.0-11.3%)
DA	15	273	127 (47%)	21	39% (106/273)	17% (18/106)	26% (28/106)	57% (60/106)	10.4% (28/273)	(7.0-14.7%)
DA	16	177	100 (56%)	19	18.5% (33/177)	52% (16/31)	26% (8/31)	31% (9/31)	3.4% (6/177)	(1.3-7.2%)
DA	17	392	204 (52%)	19	14% (56/392)	32% (18/56)	38% (21/56)	30% (17/56)	2.3% (9/392)	(1.1-4.3%)
DA	19	223	120 (54%)	24	18.7% (41/223)	44% (18/41)	46% (19/41)	10% (4/41)	1.3% (3/223)	(0.3-3.7%)
DA	21	64	34 (53%)	20	12.5% (8/64)	50% (4/8)	12.5% (1/8)	37.5% (3/8)	1.6% (1/64)	(0.0-8.4%)
DA	24	323	167 (50%)	21	14% (47/323)	53% (25/47)	45% (21/47)	2% (1/47)	0.6% (2/323)	(0.0-2.2%)
TOTALS	**Totals**	**2181**	**1160 (53%)**	**21**	**23% (489/2176)**	**29% (140/489)**	**37% (182/489)**	**34% (167/489)**	**4.3% (93/2168)**	**(3.5-5.2%)**

12 months

MDA Treatment	Village	# enrolled	# of males	Median age	% FTS +	% FTS+ Score 1 (Weak)	% FTS+ Score 2 (Medium)	% FTS+ Score 3 (Strong)	% MF+

DA	3	192	89 (46%)	22.5	24% (45/190)	38% (17/45)	40% (18/45)	22% (10/45)	2% (4/190)	(0.6-5.3%)
DA	5	90	51 (57%)	20.5	17% (15/90)	69% (10/15)	20% (3/15)	13% (2/15)	0% (0/90)	
DA	7	131	70 (55%)	18	13% (16/126)	37% (6/16)	31% (5/16)	31% (5/16)	0% (0/126)	
DA	9	107	48 (45%)	20	2% (2/107)	100% (2/2)	0% (0/2)	0% (0/2)	0% (0/107)	
DA	11	152	88 (58%)	24	34% (51/151)	27% (14/51)	39% (20/51)	33% (17/51)	4% (6/151)	(1.5-8.5%)
DA	13	219	120 (55%)	19	31% (68/219)	22% (15/68)	34% (23/68)	44% (30/68)	3% (6/219)	(1.1-5.9%)
DA	15	282	135 (48%)	19	25% (70/278)	16% (11/70)	18% (13/70)	66% (46/70)	2.5% (7/278)	(1.0-5.1%)
DA	16	116	69 (59%)	23.5	18% (21/116)	43% (9/21)	19% (4/21)	38% (8/21)	2.6% (3/116)	(0.5-7.4%)
DA	17	263	137 (52%)	18	10% (26/263)	38% (10/26)	42% (11/26)	20% (5/26)	0.4% (1/263)	(0.0-2.1%)
DA	19	161	80 (50%)	18	13% (21/161)	48% (10/21)	28% (6/21)	24% (5/21)	0% (0/161)	
DA	21	61	30 (48%)	19	10% (6/61)	66% (4/6)	34% (2/6)	0% (0/6)	1.6% (1/61)	(0.0-8.8%)
DA	24	189	106 (56%)	23	9% (17/189)	65% (11/17)	23% (4/17)	12% (2/17)	0.5% (1/189)	(0.0-2.9%)
TOTALS	**Totals**	**1963**	**1023 (52%)**	**20**	**18% (358/1963)**	**33% (119/358)**	**30% (109/358)**	**36% (130/358)**	**1.5% (29/1963)**	**(1.0-2.1%)**

				**24 months**						
**MDA Treatment**	**Village**	**# enrolled**	**# of males**	**Median age**	**% FTS +**	**% FTS+ Score 1 (Weak)**	**% FTS+ Score 2 (Medium)**	**% FTS+ Score 3 (Strong)**	**% MF+**	
DA	3	155	82 (53%)	17	13.5% (21/155)	24% (5/21)	33% (7/21)	43% (9/21)	0% (0/155)	
DA	5	73	41 (56%)	20	3% (2/73)	100% (2/2)	0% (0/2)	0% (0/2)	0% (0/73)	
DA	7	115	63 (55%)	19	7% (8/115)	50% (4/8)	25% (2/8)	25% (2/8)	0% (0/115)	
DA	9	71	26 (37%)	17	3% (2/71)	100% (2/2)	0% (0/2)	0% (0/2)	0% (0/71)	
DA	11	107	66 (62%)	19	31% (33/107)	27% (9/33)	36% (12/33)	36% (12/33)	0.9% (1/107)	(0.0-5.1%)
DA	13	216	134 (62%)	19	18% (39/216)	26% (10/39)	20% (8/39)	49% (19/39)	1.4% (3/216)	(0.3-4.0%)
DA	15	248	126 (51%)	16	22% (54/248)	24% (13/54)	13% (7/54)	63% (34/54)	0.8% (2/248)	(0.1-2.9%)
DA	16	66	39 (59%)	18	4.5% (3/66)	67% (2/3)	33% (1/3)	0% (0/3)	0% (0/66)	
DA	17	297	161 (54%)	18	1.7% (5/297)	40% (2/5)	40% (2/5)	20% (1/5)	0.3% (1/297)	(0.0-1.9%)
DA	19	149	88 (59%)	18	1.3% (2/149)	50% (1/2)	0% (0/2)	50% (1/2)	0% (0/149)	
DA	21	71	35 (49%)	18	3% (2/71)	0% (0/2)	0% (0/2)	100% (2/2)	1.4% (1/71)	(0.0-7.6%)
DA	24	276	141 (51%)	21	5% (13/276)	77% (10/13)	23% (3/13)	0% (0/13)	0% (0/276)	
TOTALS	**Totals**	**1844**	**1002 (54%)**	**19**	**10% (184/1840)**	**33% (60/184)**	**23% (42/184)**	**43% (80/184)**	**0.4% (8/1844)**	**(0.2-0.9%)**

MDA treatment	Village #	Age 5-9 years males			Age 5-9 years females			Age 10-17 years males			Age 10-17 years females			Age 18+ years males			Age 18+ years females		
Baseline		*Census*	*MDA*	*Coverage*	*Census*	*MDA*	*Coverage*	*Census*	*MDA*	*Coverage*	*Census*	*MDA*	*Coverage*	*Census*	*MDA*	*Coverage*	*Census*	*MDA*	*Coverage*
IDA	1	26	14	53.8	26	12	46.2	36	22	61.1	32	21	65.6	77	59	76.6	78	60	76.9
IDA	2	40	9	22.5	38	11	28.9	52	41	78.8	54	36	66.7	157	104	66.2	132	100	75.8
IDA	4	16	1	6.3	12	1	8.3	23	13	56.5	15	11	73.3	47	36	76.6	33	23	69.7
IDA	6	27	6	22.2	17	6	35.3	27	18	66.7	18	16	88.9	81	57	70.4	72	41	56.9
IDA	8	46	20	43.5	30	13	43.3	57	54	94.7	56	47	83.9	167	109	65.3	135	91	67.4
IDA	10	12	5	41.7	15	7	46.7	27	19	70.4	21	14	66.7	51	37	72.5	50	37	74.0
IDA	12	32	13	40.6	30	11	36.7	27	23	85.2	47	40	85.1	98	92	93.9	95	84	88.4
IDA	14	60	21	35.0	47	19	40.4	60	49	81.7	65	49	75.4	201	163	81.1	151	103	68.2
IDA	18	33	10	30.3	20	4	20.0	44	21	47.7	53	23	43.4	134	75	56.0	123	68	55.3
IDA	20	12	9	75.0	19	11	57.9	36	30	83.3	31	25	80.6	63	62	98.4	61	60	98.4
IDA	22	14	3	21.4	4	1	25.0	20	10	50.0	18	15	83.3	42	20	47.6	48	29	60.4
IDA	23	10	3	30.0	16	4	25.0	37	25	67.6	18	13	72.2	56	36	64.3	54	37	68.5
**Totals**		**328**	**114**		**274**	**100.0**		**446**	**325.0**		**428**	**310.0**		**1174**	**850.0**		**1032**	**733.0**	
**Mean % coverage**				**34.8**			**36.5**			**72.9**			**72.4**			**72.4**			**71.0**
**SD**				**17.0**			**13.1**			**14.2**			**11.9**			**13.8**			**11.9**

MDA treatment	Village #	Age 5-9 years males			Age 5-9 years females			Age 10-17 years males			Age 10-17 years females			Age 18+ years males			Age 18+ years females		
12 months		*Census*	*MDA*	*Coverage*	*Census*	*MDA*	*Coverage*	*Census*	*MDA*	*Coverage*	*Census*	*MDA*	*Coverage*	*Census*	*MDA*	*Coverage*	*Census*	*MDA*	*Coverage*
IDA	1	26	7	26.9	26	10	38.5	36	31	86.1	32	18	56.3	77	47	61.0	78	46	59.0
IDA	2	40	8	20.0	38	30	78.9	52	22	42.3	54	31	57.4	157	66	42.0	132	61	46.2
IDA	4	16	5	31.3	12	7	58.3	23	17	73.9	15	13	86.7	47	28	59.6	33	29	87.9
IDA	6	27	5	18.5	17	5	29.4	27	13	48.1	18	15	83.3	81	33	40.7	72	51	70.8
IDA	8	46	22	47.8	30	15	50.0	57	46	80.7	56	54	96.4	167	84	50.3	135	61	45.2
IDA	10	12	3	25.0	15	12	80.0	27	21	77.8	21	19	90.5	51	37	72.5	50	41	82.0
IDA	12	32	21	65.6	30	21	70.0	27	33	122.2	50	27	54.0	92	72	78.3	84	66	78.6
IDA	14	60	51	85.0	47	35	74.5	70	74	105.7	65	73	112.3	201	171	85.1	151	154	102.0
IDA	18	33			20			44	20	45.5	53	22	41.5	134	66	49.3	123	38	30.9
IDA	20	12	7	58.3	19	8	42.1	30	26	86.7	31	35	112.9	62	48	77.4	61	48	78.7
IDA	22	14	4	28.6	4	5	125.0	20	12	60.0	18	17	94.4	42	25	59.5	48	34	70.8
IDA	23	10	2	20.0	16	4	25.0	37	12	32.4	18	17	94.4	56	40	71.4	54	18	33.3
**Totals**		**328**	**135.0**		**274**	**152.0**		**450**	**327.0**		**431**	**341.0**		**1167**	**717.0**		**1021**	**647.0**	
**Mean % coverage**				**41.2**			**55.5**			**72.7**			**79.1**			**61.4**			**63.4**
**SD**				**21.2**			**27.5**			**25.9**			**22.7**			**14.1**			**21.5**

MDA treatment	Village #	Age 5-9 years males			Age 6-9 years females			Age 10-17 years males			Age 10-17 years females			Age 18+ years males			Age 18+ years females		
Baseline		*Census*	*MDA*	*Coverage*	*Census*	*MDA*	*Coverage*	*Census*	*MDA*	*Coverage*	*Census*	*MDA*	*Coverage*	*Census*	*MDA*	*Coverage*	*Census*	*MDA*	*Coverage*
DA	3	27	7	25.9	26	7	26.9	35	26	74.3	24	19	79.2	97	47	48.5	77	23	29.9
DA	5	18	10	55.6	17	2	11.8	19	9	47.4	19	9	47.4	54	40	74.1	45	34	75.6
DA	7	20	3	15.0	19	4	21.1	18	13	72.2	22	16	72.7	52	36	69.2	52	28	53.8
DA	9	6	4	66.7	17	6	35.3	9	5	55.6	13	12	92.3	48	26	54.2	39	24	61.5
DA	11	25	5	20.0	11	6	54.5	22	19	86.4	17	16	94.1	61	52	85.2	54	39	72.2
DA	13	31	7	22.6	29	4	13.8	74	34	45.9	53	33	62.3	136	73	53.7	130	86	66.2

DA	15	36	26	72.2	27	23	85.2	50	36	72.0	57	34	59.6	99	68	68.7	104	91	87.5
DA	16	19	13	68.4	13	9	69.2	34	30	88.2	21	8	38.1	51	42	82.4	47	39	83.0
DA	17	46	20	43.5	45	29	64.4	69	68	98.6	49	45	91.8	159	116	73.0	147	104	70.7
DA	19	36	5	13.9	33	11	33.3	57	45	78.9	32	25	78.1	107	70	65.4	108	67	62.0
DA	21	13	2	15.4	11	1	9.1	27	13	48.1	16	10	62.5	52	19	36.5	48	19	39.6
DA	24	72	16	22.2	83	18	21.7	93	49	52.7	79	41	51.9	226	102	45.1	202	97	48.0
**Totals**		**304**	**118.0**		**288**	**120.0**		**453**	**347.0**		**359**	**268.0**		**991**	**691.0**		**931**	**651.0**	
**Mean % coverage**				**38.8**			**41.7**			**76.6**			**74.7**			**69.7**			**69.9**
**SD**				**22.0**			**24.1**			**17.2**			**17.8**			**14.6**			**16.5**

**MDA treatment**	**Village #**	**Age 5-9 years males**			**Age 6-9 years females**			**Age 10-17 years males**			**Age 10-17 years females**			**Age 18+ years males**			**Age 18+ years females**		
12 months		***Census***	***MDA***	***Coverage***	***Census***	***MDA***	***Coverage***	***Census***	***MDA***	***Coverage***	***Census***	***MDA***	***Coverage***	***Census***	***MDA***	***Coverage***	***Census***	***MDA***	***Coverage***
DA	3	27	3	11.1	26	6	23.1	35	22	62.9	24	27	112.5	97	64	66.0	77	63	81.8
DA	5	18	10	55.6	17	6	35.3	19	12	63.2	19	9	47.4	54	29	53.7	45	23	51.1
DA	7	20	13	65.0	19	9	47.4	18	15	83.3	22	22	100.0	52	42	80.8	52	25	48.1
DA	9	7	7	100.0	17	13	76.5	11	9	81.8	13	15	115.4	48	30	62.5	39	30	76.9
DA	11	25	13	52.0	11	2	18.2	22	19	86.4	17	16	94.1	61	56	91.8	54	45	83.3
DA	13	31	8	25.8	29	12	41.4	74	39	52.7	53	31	58.5	136	73	53.7	130	65	50.0
DA	15	36	28	77.8	27	21	77.8	50	37	74.0	57	39	68.4	99	70	70.7	104	81	77.9
DA	16	13	12	92.3	13	7	53.8	25	12	48.0	11	9	81.8	51	45	88.2	42	31	73.8
DA	17	46	19	41.3	45	17	37.8	69	42	60.9	49	44	89.8	159	76	47.8	147	65	44.2
DA	19	36	9	25.0	33	8	24.2	57	38	66.7	32	31	96.9	107	53	49.5	108	41	38.0
DA	21	13	3	23.1	11	6	54.5	27	23	85.2	16	9	56.3	52	34	65.4	48	17	35.4
DA	24	72	18	25.0	83	9	10.8	93	55	59.1	79	39	49.4	226	82	36.3	202	103	51.0
**Totals**		**344**	**143.0**		**331**	**116.0**		**500**	**268.0**		**392**	**291.0**		**1142**	**654.0**		**1048**	**589.0**	
**Mean % coverage**				**41.6**			**35.0**			**53.6**			**74.2**			**57.3**			**56.2**
**SD**				**28.1**			**20.6**			**12.6**			**23.2**			**16.2**			**17.2**

“Community Based Safety Study of 2-drug (Diethylcarbamazine and Albendazole) versus 3-drug (Ivermectin, Diethylcarbamazine and Albendazole) Therapy for Lymphatic Filariasis in Papua New Guinea”

Protocol Identifier: DOLF_IDA_Papua New Guinea

Type: Community Based Mass Drug Administration

DOLF Project Principal Investigators: Gary Weil, MD, Washington University, USA and Christopher King, MD, PhD, MPH, Case Western Reserve University, USA

Study Site Principal Investigators: Leanne Robinson, PhD, MPH and William Pomat, PhD, Papua New Guinea Institute for Medical Research, Papua New Guinea

Initial Protocol: v1.0 22 April 2016

Amendment 1: v2.0 19 May 2016

Amendment 2: v2.1 05 July 2017

INVESTIGATOR AGREEMENT

"Community Based Safety Study of 2-drug (Diethylcarbamazine and Albendazole) versus 3-drug (Ivermectin, Diethylcarbamazine and Albendazole) Therapy for Lymphatic Filariasis in Papua New Guinea"

DOLF_IDA_Papua New Guinea: v2.1 05 July 2017

I have read the protocol, including the appendices, and I agree that it contains all necessary details for me and my staff to conduct this study as described. I will conduct this study as outlined and make a reasonable effort to complete the study within the time designated.

I will provide all study personnel, participating in the study under my supervision copies of the protocol and access to all study related information provided by the DOLF project. I will discuss with them to ensure they are full informed about the study drug(s) and the study procedures.

Principle Investigator: ____

Name/Title (Print/Type)

Signed: ____ **Date:** ________________

NOTE: Both the Project PI and local PI should have signed investigator agreements on file.

Co-Investigators:

Center for Global Health and Diseases, Case Western Reserve University, Cleveland, OH, USA
James Kazura, MD
Ronald Blanton, MD
Daniel J Tisch, PhD

Department of Medicine, Washington University School of Medicine, St. Louis, MO, USA
Gary Weil, MD

PNG Institute for Medical Research
Moses Laman, MBBS, PhD
Livingstone Tavul, MSc

PNG National Department of Health
Sibauk Bieb, MBBS
Lucy John, MBBS
Leo Makita Principal advisor, Malaria and Vector-Borne Diseases
Mary Yohogu, NTD Program manager

World Health Organisation (WHO) WPRO
James Wangi, MPH, MBBS, MBBE

Team Roster

Principal Investigators

1.1.1

1.1.2 Christopher L King, MD, PhD

Center for Global Health and Diseases
Biomedical Research Building
2109 Adelbert Rd.
Case Western Reserve University School of Medicine
Cleveland, OH, 44106-4983
Tel 216-368-4817, email: cxk21@case.edu

Leanne Robinson, PhD, MPH
Papua New Guinea Institute of Medical Research (PNG IMR)
Madang, PNG
Tel 675-422-2909, email: robinson@wehi.EDU.AU

William Pomat, PhD
PNG IMR

Goroka, PNG
Tel 675-532-2800, email: william.pomat@pngimr.org.pg

1.1.3 Investigators

PNG IMR
Madang, PNG
Tel 675-852-2909

Moses Laman, MBBS, PhD email: drmlaman@yahoo.com
Livingstone Tavul, MSc email: ltteruah1@gmail.com

Center for Global Health and Diseases
Biomedical Research Building
2109 Adelbert Rd.
Case Western Reserve University School of Medicine
Cleveland, OH, 44106-4983

1.1.4

1.1.5 James W Kazura, MD

Tel 216-368-4810; email jxk14@case.edu

1.1.6 Ronald E Blanton, MD

Tel 216-368-4814; email reb6@case.edu

Daniel J Tisch, MPH, PhD email: dxt37@case.edu

PNG National Department of Health

Sibauk Beib, MBBS email: svbeib@gmail.com
Lucy John, MBBS email: Lucy_John@health.gov.pg
Leo Makita email: leo.makita@gmail.com
Mary Yohogu email: myohogu51@gmail.com

WHO WPRO
James Wangi, MPH, MBBS, MBBE email: wangij@wpro.who.int/pg

Protocol Statisticians
Daniel J Tisch, MPH, PhD
Case Western Reserve University School of Medicine
Center for Global Health & Diseases, Dept. of Epidemiology and Biostatistics

2109 Adelbert Road,
Cleveland, OH, USA 44106
Tel. 216 368 4818 Fax: 216 368 4825 email: dxt37@case.edu

Ken Schechtman, PhD
Dept. of Internal Medicine and Division of Biostatistics, Washington University School of Medicine
660 South Euclid, St. Louis, MO 63110
Tel. 314-454-8356; Fax 314-454-5113; email: wshannon@im.wustl.edu

1.1.6.1.1.1.1 Medical Monitor

Charles H King, MD, MS
Center for Global Health and Diseases, Case Western Reserve University School of Medicine, Cleveland, OH

1.1.6.1.1.1.2 PARTICIPATING SITES

Case Western Reserve University
IRB approval and oversight provided by University Hospitals Case Medical Center Cleveland, OH USA

PNG IMR
Madang and Maprik, PNG
IRB approval and oversight provided by PNG IMR, Goroka and the PNG Medical Research Advisory Committee (MRAC), Port Moresby

PNG National Department of Health (PNG NDoH)
Vector Borne Disease Control Program; Lymphatic Filariasis Elimination Program

LAYMAN PROTOCOL SUMMARY

Results from ongoing studies conducted in East Sepik Province, PNG have shown that using three drugs together (Ivermectin [IVM], Diethylcarbamazine [DEC] and Albendazole [ALB], i.e., "IDA") is more effective than the current two-drug combination (DEC+ALB) being used in the global program to eliminate lymphatic filariasis in PNG. Lymphatic filariasis (LF) elimination in PNG involves annual mass drug administration (MDA) *with the current standard two drug regimens in LF endemic areas for at least 5 years*.

A single dose of the three drugs together, IDA, very quickly cleared all lymphatic filariasis "worms" or "microfilariae" (Mf) from the blood of 68 heavily infected individuals with LF in PNG. All treated participants remained Mf negative one year after treatment, something that is only achieved in ~20 to 30% of individuals treated with the standard two-drug combination in PNG. A single dose of the triple drug combination therefore appears to kill or permanently sterilize adult worms.

This new treatment could make a huge difference in the global effort to eliminate LF in PNG and other LF endemic countries by reducing the number of rounds of Mass Drug Administration (MDA) required for elimination. i.e., completely and irrevocably eliminating transmission of LY by the local mosquito vectors. The greatest impact of this new treatment may be in areas with high infection rates where MDA has not yet been introduced, and also could be very useful areas where LF infection persists despite several years of annual MDA with the current 2-drug regimens. In PNG, both areas exist and continue to present a challenge to LF elimination efforts.

Although all of the individual drugs used in the IDA triple drug combination have been provided as MDA to hundreds of millions of people since 2000, there is only limited experience with the three drugs used together. Currently a total of 120 participants with heavy LF infections have been treated with the triple drug combination in clinical trials in PNG and Cote d'Ivoire. Many of the LF infected people given IDA experienced brief side effects that are commonly associated with two drug regimens that include DEC+ALB (in PNG and other countries in the Pacific and Asia) or IVR+ALB (in various regions of sub-Saharan Africa). Side effects included fever, headache, myalgia, and dizziness that usually resolved within 24 to 48 hours. The frequency of adverse events (AE) was higher in triple drug IDA treatment compared to standard two-drug treatment. However, the overall severity of the AEs was similar between IDA and the two drug regimens and no serious AE's requiring hospitalization occurred.

Although there is little doubt that the IDA triple drug combination is more effective than current two-drug MDA combinations for LF elimination, more safety data are urgently needed before IDA can be rolled out as an MDA regimen for millions of people. The current two-drug MDA combination for LF was studied in closely monitored community trials before being endorsed for widespread use. Similar data are now required for the new triple drug IDA combination. The Bill & Melinda Gates Foundation is willing to financially support such studies in four countries, including PNG.

The primary objective of the trial will be to study the safety and tolerability of the triple drug IDA combination, by comparing the number and type of adverse events that develop after a single treatment of LF infected and uninfected participants with IDA or DEC+ALB (the current standard regimen) in LF endemic communities in PNG. Secondary objectives will be to i) compare how much more effective the IDA combination is than DEC+ALB in killing microfilariae in community settings; and ii) compare community acceptance of MDA with the triple drug IDA compared to the two-drug combination of DEC+ALB.

TABLE OF CONTENTS

INVESTIGATOR AGREEMENT 2
1.1.1 3
1.1.2 Christopher L King, MD, PhD 3
1.1.3 Investigators....................4
1.1.4 4
1.1.5 James W Kazura, MD 4
1.1.6 Ronald E Blanton, MD....................4
TABLE OF CONTENTS....................8
2 PROTOCOL SUMMARY 13
3 BACKGROUND INFORMATION AND RATIONALE 16
3.1 Country Specific Background....................17
4 POTENTIAL RISKS AND BENEFITS 19
4.1 Risks of Blood Draw 19
4.2 Risks of Study Drugs 19
4.3 Potential Participant and Community Benefit....................19
4.4 Study Participation and Cost....................20
4.5 Compensation for Injury....................20
5 STUDY DESIGN AND OBJECTIVES 21
5.1 Study Objectives....................21
5.1.1 Secondary Objectives 21
5.2 Study Design 21
5.3 Study Screening and Enrollment....................21
5.3.1 Study Site 21
5.4 Preparatory Activities....................22
5.4.1 Social Mobilization 22
5.4.2 Household Enumeration, Census and Geo-Referencing 22
5.5 Pre-Treatment Assessment Team 22
5.6 Inclusion and Exclusion Criteria....................22
5.7 Pregnant Females 23
5.8 Informed Consent....................23
5.9 Baseline Survey....................24
5.10 Screening for Filarial Antigenemia and Microfilaria 24
5.11 Assessment of Efficacy of IDA on STH (including *Strongyloides* by qPCR*)* 24
5.12 Randomization....................25
5.13 Withdrawal....................25
5.14 Efficacy and Effectiveness of IDA vs DA....................26
5.15 Retreatment....................26
5.16 Guidelines for Stopping the Trial....................26
5.17 Triple Drug Regimen Acceptability....................27
6 INVESTIGATIONAL PRODUCT 28
6.1 Study Drug Background....................28

6.1.1 Product Supply and Storage 29
7 STUDY PROCEDURES/EVALUATIONS/SCHEDULE 30
7.1 Triple Drug Therapy (IDA) and Two-Drug Therapy (DA) 30
7.2 Overall Study Schedule 30
8 SAFETY REPORTING AND SAFETY MONITORING 31
8.1 Definitions 31
8.2 Assessment of Adverse Events 32
8.2.1 Serious Adverse Event (SAE) Assessment and Management 32
8.3 Reporting of Pregnancy 33
8.4 Safety Monitoring by the Oversight Committee 33
9 CLINICAL MANAGEMENT OF EVENTS 34
9.1 Adverse Event Monitoring and Management 34
9.1.1 Mild Localized Symptoms 34
9.1.2 Moderate to Severe Localized Adverse Events 34
9.1.3 Moderate to Severe Systemic Adverse Events 35
9.2 Rapid Response Teams for Management of Adverse Events 35
10 STATISTICAL CONSIDERATIONS 36
10.1 Safety 36
10.2 Efficacy 36
10.3 Enrolling Additional Participants 36
11 DATA HANDLING/RECORD KEEPING/SOURCE DOCUMENTS 37
11.1 Types of Data Collected 37
11.2 Study Records Retention 38
11.3 Source Documents 38
12 RESPONSIBILITIES 39
12.1 Investigator Responsibilities 39
12.1.1 Good Clinical Practice 39
12.2 Institutional Review Board (IRB)/Ethics Committee (EC) 39
12.3 Informed Consent 39
12.3.1 Informed Consent Training 39
12.3.2 Country Specific ICF Information 40
12.4 Participant Privacy 40
12.5 Data Ownership 40
13 PUBLICATION POLICY 42
14 LITERATURE REFERENCES 43
LIST OF APPENDICES 45
Appendix 1: Study Flow Diagram (Country Specific) 46
Appendix 2: Participant Enrollment Form [Example] 47
Appendix 3: Participant Monitoring Form [Example] 50
Appendix 4: Guide To Assigning Adverse Event Severity 53
Appendix 5: Adverse Event Evaluation and Report Form (AEERF) [Example] 56
Appendix 5a: Required Reporting Guideline For Serious Adverse Events 60
Appendix 6A: Informed Consent Form [Example] 64

Appendix 6B: Informed re-Consent Form (V.1, 24 may 2017) **Error! Bookmark not defined.**
Appendix 7: Treatment Acceptability Study Protocol..73

LIST OF ABBREVIATIONS

GENERAL PROJECT ABBREVIATIONS

AE	Adverse Event/Adverse Experience
AEERF	Adverse Event Evaluation and Report Form
AFC	Anti-Filariasis Campaign
Ag	antigenemia
ALB	Albendazole
CDD	Community Drug Distributor
CRF/eCRF	Case Report Form also referred to as eCRF (electronic case report form)
DA	Two Drug Therapy (dyethilcarbamzine and albendazole)
DEC	Diethylcarbamazine
DOLF	Death for Onchocerciasis and Lymphatic Filariasis
DOT	Directly Observed Treatment
DSMB, DSRB or DMC	Data and Safety Monitoring Board also called Data Safety Review Board or Data Monitoring Committee
EC	Ethics Committee (may also be called IRB or Institutional Review Board)
EDC	Electronic Data Capture
FTS	Filariasis Test Strip
GCP	Good Clinical Practice
GPELF	Global Programme to Eliminate Lymphatic Filariasis
GPS	Global Positioning System
ICF	Informed Consent Form
ICH	International Conference on Harmonization
IDA	Triple Drug Therapy (Ivermectin, Diethylcarbamazine,and Albendazole)
IMA	IMA World Health

IRB	Institutional Review Board (may also be called EC)
IVM	Ivermectin
LF	Lymphatic Filariasis
MDA	Mass Drug Administration
MF	Microfilaria(e)
Mg	Milligram
NTD	Neglected Tropical Diseases
NLM	National Library of Medicine
PHM	Public Health Midwife
PI	Principal Investigator
SAE	Serious Adverse Event/Serious Adverse Experience
SOP	Standard Operating Procedure
TAS	Transmission Assessment Surveys
UNID	Unique Study Identification Numbers
UR	University of Ruhuna
USAID	United States Agency for International Development
WHO	World Health Organization

COUNTRY SPECIFIC ABBREVIATIONS

PNGIMR	Papua New Guinea Institute for Medical Research

2 PROTOCOL SUMMARY

**Study Title:**	Community Based Safety Study of 2-drug (Diethylcarbamazine and Albendazole) versus 3-drug (Ivermectin, Diethylcarbamazine and Albendazole) Therapy for Lymphatic Filariasis in Papua New Guinea
**Type of Study:**	Mass Drug Administration
**Population:**	IDA/ Triple Drug Arm: participants more than or equal to 5 years of age<br>DA/ Dual Drug Arm (DA): participants more than or equal to 5 years of age
**Number of Treated Areas:**	Study sites will be communities in Madang Province, Bogia District and as alternatives, Dreikiker District, East Sepik Province and East New Britian Province
**Duration of Study Participation**	Single treatment with daily adverse event follow-up thru Day 7, then a long-term follow-up visit at 1 year.
**Study Drugs**	Ivermectin (3 mg tablets) *not included in two arm treatment<br>Diethylcarbamazine (100 mg tablets)<br>Albendazole (400 mg tablets)
**Primary Objective:**	Determine the frequency, type, and severity of adverse events following triple drug therapy (IVM+DEC+ALB) compared to the standard two drug treatment (DEC+ALB) in infected and uninfected individuals in a community
**Secondary Objectives:**	Compare the efficacy of IDA (3 drug therapy) to DA (2 drug therapy) administered in communities for clearance of MF and filarial antigenemia (Ag)<br>Assess the effect of intensity of filarial infection on the frequency and severity of adverse events<br>Compare community acceptance of Mass Drug Administration with three drug vs two drug therapy<br>To examine the impact of IDA vs DA on transmission of LF.<br>To evaluate the impact of IDA vs. DA on reduction of scabies and other skin infections.

**DOLF PROJECT**	This protocol is specific to Papua New Guinea, but results will also be included in the larger DOLF project. Data will be available/reviewed at a country level and at the project level.

STUDY DESIGN

General Flow Diagram:

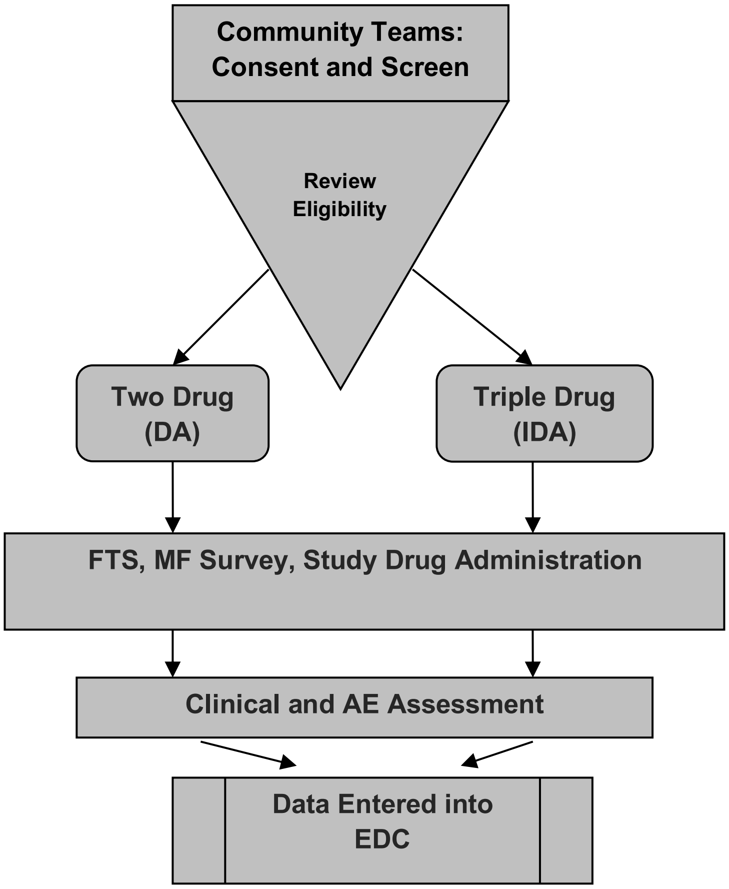

**DOLF_IDA_Papua New Guinea Study**	**ARM 1**	**Sample Size: 3000**	**Triple Drug**
	**ARM 2**	**Sample Size: 3000**	**Two Drug**

NOTE: A Study Flow Diagram specific for Papua New Guinea is provided in Appendix 1.

3 BACKGROUND INFORMATION AND RATIONALE

LF is a parasitic worm infection where adult male and adult female worms that have mated in the lymph nodes of the human lymphatic system release immature forms (microfilaria or MF) that appear in the blood stream at night. These MF are taken up by mosquitoes, after which they develop to infective forms over 1-2 weeks that continue the parasite lifecycle when blood seeking female mosquitoes again bite a potential human host. Dying adult worms provoke disabling and disfiguring obstruction of the lymphatic vessels. In 2000, the World Health Organization (WHO) launched the Global Programme to Eliminate Lymphatic Filariasis (GPELF) to eliminate lymphatic filariasis as a public health problem by 2020. To interrupt transmission, WHO recommends therapy using combinations of two medicines delivered to entire at-risk populations through a strategy known as mass drug administration (MDA). Ivermectin and albendazole are administered in areas where onchocerciasis is co-endemic; diethylcarbamazine and albendazole are administered in areas where onchocerciasis is not co-endemic.

Results of a pilot study in Papua New Guinea suggest that triple drug therapy (ivermectin, diethylcarbamazine and albendazole) is superior to the currently recommended two-drug regimen [11]. A single dose of the triple therapy rapidly achieved complete clearance of *Wuchereria bancrofti* microfilariae from the blood of 12 individuals for at least one year post-treatment. All six individuals tested at 24 months were still amicrofilaremic, suggesting that the triple therapy might permanently sterilizes adult filarial worms. Many people treated in these studies experienced transient systemic adverse events commonly associated with diethylcarbamazine or ivermectin treatment of filariasis. Adverse events were more frequent after the triple therapy than after the usual combination of two drugs. However, no serious adverse events were observed. Preliminary results from two larger clinical trials in Papua New Guinea and in Cote d'Ivoire (West Africa) are consistent with results from the pilot study. The dramatic reduction and sustained clearance of microfilaremia along with the safety profiles seen in these studies suggest that the triple drug therapy may be a useful tool for achieving the goal of eliminating lymphatic filariasis as a public health problem by 2020.

Although the studies mentioned above have clearly demonstrated the superiority of the triple drug therapy for clearing *W. bancrofti* microfilariae from the blood, more safety and efficacy data are needed before triple therapy can be rolled out on a large scale as a mass drug administration regimen in lymphatic filariasis endemic countries. WHO recommends a best practice called “cohort event monitoring” for demonstrating safety of new drug regimens for public health program use. Establishing safety through such methodology requires pre and post treatment assessments from at least 10,000 people treated with the triple therapy across multiple settings.

The inclusion of IVM to an MDA regimen also provides additional public health benefits, since it complements the deworming effect of ALB , a global initiative for the control of gastrointestinal worm infections (e.g., hookworm, Ascaris, Trichuris, Strongyloides) and eliminates lice and scabies mites (4).

3.1 Country Specific Background

Papua New Guinea (PNG) has some of the most heavily infected populations with lymphatic filariasis in world and most areas in PNG have never received any treatment for lymphlatic filariasis. Adverse events (AEs) following MDA are directly related the burden of infection, especially the microfilarial levels which are rapidly killed by ivermectin [IVM] and diethylcarbamazine [DEC]. The addition of IVM to existing treatment regimen with DEC+ALB might be expected to increase AEs, especially in individuals with high MF levels as was observed PNG (Thomsen, et al CID, 2016). Thus studies in PNG will be crucial to establish the safety of triple drug therapy in participant with high microfilaria level of the principal human filarial parasite *W. bancrofti*. Results from ongoing studies conducted in East Sepik Province have shown that using three drugs together (ivermectin [IVM], diethylcarbamazine [DEC] and albendazole [ALB]) is more effective than the current two-drug combination (DEC/ALB) being used in the global program to eliminate lymphatic filariasis in Papua New Guinea. Lymphatic filariasis elimination in PNG involves annual mass drug administration (MDA) in lymphatic filariasis (LF) endemic areas for at least 5 years.

A single dose of the three drugs together very quickly cleared all lymphatic filariasis “worms” or “microfilariae” (MF) from the blood of 68 heavily infected with LF in PNG. All but two treated participants remained MF negative 1 year after treatment, something that is only achieved in ~ 20 to 30% of individuals treated with the standard two-drug combination in PNG. The two MF positive individuals at one year had only one MF in 2 mls of blood, a level unlikely to be transmissible. Moreover these individuals lived in a community with exceptionly high transmission and it is possible that they may be been re-infected during the course of year followup. A single dose of the triple drug combination therefore appears to kill or permanently sterilize adult worms. In this larger study of triple drug treatment, AEs were slightly higher compared to the standard two drug regimen, but this difference was not significantly different. There was one severe AE in a participant that received the triple drug regimen, however this resolved within 24 hours, did not require hospitalization, and was deemed not be an SAE by medical professional evaluating the individual.

This new treatment could make a huge difference in the global effort to eliminate LF by reducing the number of rounds of MDA required for elimination. The greatest impact of this new treatment may be in areas with high infection rates where MDA has not yet been introduced, but it also could be very useful for areas where LF infection persists despite several years of annual MDA with current 2-drug regimens. In PNG, both areas exist and continue to present a challenge to LF elimination efforts.

The WHO, academic experts, and the donor community are excited, because IDA has the potential to accelerate LF elimination around the world. Although the studies cited above have clearly demonstrated the superiority of the IDA regimen for clearing *W. bancrofti* MF, more safety and efficacy data are needed before IDA can be rolled out on a large scale. The WHO and Bill & Melinda Gates Foundation have accepted the efficacy data, but in order to elevate this approach to WHO policy and obtain increased drug company donation, it will be necessary

to obtain evidence of an acceptable AE profile in large multi-center international studies. WHO recommends a best practice called “cohort event monitoring” for demonstrating the safety of new drug regimens for public health program use (see A Practical Handbook on the Pharmacovigilance of Medicines used in the Treatment of Tuberculosis, World Health Organization, Geneva, 2012). Establishing the safety of IDA for MDA through such methodology requires pre- and post-treatment assessment from at least 10,000 people treated across multiple settings. The current two-drug MDA regimens were studied in closely monitored community trials in a similar manner before they were endorsed for widespread use by the GPELF. This study is designed to obtain data on the safety and effectiveness of IDA therapy in the PNG population to guide future use of the therapy for the treatment of LF in PNG and to be included in the larger data set from all contributing countries to advance IDA therapy for use in elimintating LF wordwide.

4 POTENTIAL RISKS AND BENEFITS

4.1 Risks of Blood Draw

Blood collection via finger prick is considered to be minimal risk and little or no discomfort is anticipated. The risk of infection is minimized by the use of standard sterile techniques. On occasion a participant may faint during or after the finger prick. Study personnel will be alert to participant reactions after the blood collection and will provide aid as needed.

4.2 Risks of Study Drugs

The combinations of ivermectin plus albendazole or DEC plus albendazole are widely used for MDA. There also have been clinical trials of DEC plus Ivermectin and for triple drug therapy that show no significant drug interactions [11]. Risks of each drug separately, with some indication of how likely these are to occur, are summarized below:

Diethylcarbamazine (DEC): The most common side effects reported are itching and swelling of face, headache, joint pain, unusual tiredness or weakness. These are transient. Less common are dizziness, nausea or vomiting. Fever, painful and tender glands in groin, neck and armpits or skin rash can occur, and are usually associated with high burdens of infection as judged by the level of blood microfilaremia.

Albendazole (ALB): The most common side effects reported are headache, nausea, stomach pain and vomiting and are usually associated with heavy soil-transmitted helminths infections. Severe allergic reactions occur rarely, and include rash, hives, itching, difficulty breathing, tightness in the chest, swelling of the mouth, face, lips, or tongue, dark urine. Mild elevation in liver transaminases can occur, but normalize with cessation of treatment. These AEs are usually associated with prolonged ALB therapy.

Ivermectin (IVM): The most common side effects reported are diarrhea, dizziness and nausea. Rare side effects include rash, hives, itching, difficulty breathing, chest tightness, swelling of the mouth, face, lips, or tongue, eye pain, fainting, and fast heartbeat. Mild decrease in leukocyte counts, elevated liver function tests, and cardiovascular effects that included tachycardia and orthostatic hypotension have been described. Infrequently, treatment can exacerbate bronchial asthma. These AEs are usually associated with prolonged therapy.

4.3 Potential Participant and Community Benefit

Infected participants, who sign an informed consent, will be treated for the LF infection. LF transmission to the community will be reduced by participation in either treatment arm. A broader community benefit may be facilitated by the triple drug regimen as it is believed the triple drug regimen has the potential to markedly reduce the number of MDA treatments needed to achieve transmission interruption and elimination of LF.

Both regimens provide treatment for intestinal worms, and the triple drug treatment has the added benefit of providing an effective treatment for scabies.

If the triple drug intervention proves successful, the triple therapy is likely to be adopted in many LF endemic areas globally. In order to facilitate such an uptake of triple therapy into national treatment policies, the study will be performed by Papua New Guinea Institute for Medical Research (PNGIMR) in collaboration with National Department of Health responses for the LF control program and results from this study will be combined and shared with the World Health Organization.

4.4 Study Participation and Cost

Participation is voluntary and participants may decline participation without consequences. There will be no cost to the individual to participate in the study and they will not be paid for their participation. The study will cover cost associated with laboratory test, study drugs, and clinical monitoring.

4.5 Compensation for Injury

The study drugs have been widely used for treatment of lymphatic filariasis and it is anticipated that injury resulting from treatment will be rare. In the event that a participant experiences a serious adverse event (SAE) attributable to study treatment, the project will help in supporting the medical treatment and/or hospitalization required.

In the event that a participant experiences a Serious Adverse Event attributable to treatment, the project will help support medical treatment and hospitalization required. If the participant dies as a direct consequences of treatment, then compensation to the family will be provided. It is anticipated that any injuries resulting from this study will be rare because the drugs employed have been widely used for treatment of lymphatic filariasis with very few serious adverse events.

5 STUDY DESIGN AND OBJECTIVES

5.1 Study Objectives

To determine the frequency, type and severity of adverse events following triple-drug therapy (IVM+DEC+ALB, IDA) compared to the standard two-drug treatment (DEC+ALB, DA) in infected and uninfected individuals in a community.

5.1.1 Secondary Objectives

1. To compare the efficacy of IDA vs. DA administered in communities for clearance of MF and filarial antigenemia (Ag).

2. To assess the effect of intensity of filarial infection on the frequency and severity of adverse events.

3. To compare community acceptance of MDA with IDA vs. DA.

4. To examine the impact of IDA vs DA on transmission LF.

5. To evaluate the impact of IDA vs. DA on reduction scabies and other skin infections.

5.2 Study Design

The trial will be an open labelled two-armed study. The two arms are (1) MDA with IDA (triple drug therapy) and (2) MDA with the currently used combination of DA (two-drug regimen). An overview of the study flow is provided in Appendix 1.

The primary endpoint will be the rate of AE and SAE among participants. The definitions of mild, moderate, severe and serious AE are provided in Appendix 4.

5.3 Study Screening and Enrollment

5.3.1 Study Site

Previous studies have identified the mainland and coastal islands of Northern PNG as the most effected by lymphatic filariasis (5-8). The study will be conducted in an area with high prevalence of lymphatic filariasis (LF). We have identified three potential study sites: i) Bogia District, Madang Province as the most likely study site where many of the villages average between 50 to 60% antigen positivity from a survey conducted in March 2016. Other potential sites are in areas of ii) East Sepik Province (ESP), and/or Sandaun Provinces (SP). None of these sites have previously received MDA. Another potential site is in East New Britian Province (ENB). Some areas within ENB have antigen positive rates as high as 68% based on recent screening by our study team. Other areas in ENB have low or neglible LF infection rates and some have received MDA.

5.4 Preparatory Activities

5.4.1 Social Mobilization

Prior to the administration of the drugs, intense social mobilization activities will be conducted to ensure maximum community participation. This will include development and distribution of key messages that will emphasize the acceptance and swallowing of the drugs along with their benefits and safety.

5.4.2 Household Enumeration, Census and Geo-Referencing

Health workers with the research team and community drug distributors (CDD) will enumerate and record the GPS coordinates of each house and compound within the selected study areas (PHMs) (House Visit #1, Appendix 1). A census will be performed to collect name, age and sex of each household member greater than or equal to 5 years of age. Basic information on house structure that might affect mosquito exposure to lymphatic filariasis infection, e.g. type of structure, whether screened windows present, existence of a toilet, running water, electricity and/or insecticide treated bed nets will also be collected.

5.5 Pre-Treatment Assessment Team

The pre-treatment assessment (House visit #2, Appendix 1) team will be composed of people with basic medical training able to perform a medical history and a basic physical examination (local health workers, physicians, and nursing or medical students), laboratory technicians, and community drug distributors involved in previous MDA for LF and known by the local community.

5.6 Inclusion and Exclusion Criteria

Inclusion Criteria

1. Age ≥ 5 years, for IDA and DA arms (males and females).
2. Able to provide informed consent or give parental consent for minors to participate in the trial
3. No evidence of severe or systemic co-morbidities except for features of filarial disease

Exclusion Criteria

1. Age < 5 years (ivermectin is not approved for use in children less than 5 years of age)
2. Unable to provide informed consent or give parental consent for minors to participate in the trial
3. Pregnant women (DEC, ivermectin and albendazole are not known to be safe for use during pregnancy)

4. Severe chronic illness (chronic renal insufficiency, severe chronic liver disease, or any illness that is severe enough to interfere with activities of daily living)

5. History of previous allergy to MDA drugs

5.7 Pregnant Females

Pregnant females will not be eligible to participate in this study because of the unknown effects of the drugs and drug combination used in this study. Females will be asked about the timing of the first day of their last menstrual period. Females who report that their last menstrual period started 4 weeks or longer before the interview will be excluded from the study. Females who do not recall the timing of their last menstrual period will also be excluded.

5.8 Informed Consent

A waiver of consent is being requested for the Census and Geo-referencing portion of the study prior to receiving formal consent. The study team will be collecting information about the communities and residents. This portion of the study is not greater than minimal risk and the members of the study team who are conducting the Census and Geo-referencing portion of the study will explain what they are collecting to village residents.

Individuals will be evaluated as to whether they meet the inclusion/exclusion criteria, before they give informed consent.Before any physical procedures or drug administration occur, signed consent will be obtained from participants.

Adult participants will sign a written, informed consent before the inclusion process (Appendix 6). Minors from ages 5 to 7 years do not need to give assent to the study. Respecting the cultural practices and norms in PNG, minors between the ages 7 and 12 years will not sign an assent form, but their parents will sign consent to allow their participation. This process reflects the cultural norms and practices usually employed in studies in PNG, and respect the roles that parents and the community have in the informed consent process. Minors aged 14-17 will sign the adult consent form in order to participate.

In the event that a participant is unable to read or has insufficient level of knowledge to comprehend the consent form, another villager with sufficient reading and writing skills will act a witness to the consenting process. The witness should not be involved in the implementation of the study. Participants who do not speak or read English are neither specifically included nor excluded from this study. There are hundreds of languages in Papua New Guinea. A native speaking community worker who is knowledgeable about research and the study will translate the consent form from English into the local language.

5.9 Baseline Survey

After consenting and prior to evaluation for LF infection and treatment, all individuals will be assigned a unique ID and be enrolled using a participant enrollment form (Appendix 3). Questions will be asked to each participant about their general health and last menstrual period (to establish pregnancy for women of childbearing age). Each individual will be asked if they have signs of LF complications (hydrocele, lymphedema, lymphangitis, and lymphadenitis), if they took treatment during the previous MDA for LF and if they recently took albendazole, diethylcarbamazine, or ivermectin for other conditions. Participants reporting lymphedema will be examined to identify the location and grade of the lymphedema.

Impact of IDA versus DA on LF transmission using xeno-monitoring

Mosquito collections will be conucted prior to commencement of community MDA. After consultation with community leaders as to those areas of their community where many mosquitoes are observed near or within households, light traps will be strategically placed and mosquitoes collected for 24 to 48 hours. Anopheline mosquitos known to transmit lymphatic filariasis will be collected from the light traps, pools of 10 to 25 mosquitos made, and DNA extracted from the pooled mosquitoes for PCR amplification of *W. bancrofti* DNA. The proportion of filarial infected mosquitoes will then be determined. The same xeno-monitoring will be conducted in the same villages one year following treatment.

5.10 Screening for Filarial Antigenemia and Microfilaria

Approximately 75µl of capillary blood from each eligible individual will be collected via finger prick to be deposited on the rapid diagnostic test Filariasis Test Strip (FTS, Alere™, WHO approved)) for LF antigen detection in the field. Following application of blood for the FTS, a few drops of blood will be applied to a filter paper and dried for subsequent serological evaluation of LF exposure infection. Participants with positive FTS tests will be visited at night (10 - 12 am) for microfilaria testing (60 µl measured volume blood smear- 3 lines, prepared according to the project standard operating procedure (SOP)) collected by the finger prick method.

Study participants will be informed that their blood samples may be shipped to other countries like the United States for additional parasitology testing. These samples may be stored for a longer time after the intended testing. No HIV or human genetic testing will be performed.

Universal precautions for individuals collecting and working with blood samples to include proper disposal of contaminated materials (test strips, lancets, capillary tubes, blood film slides) will be in accordance with the guidelines prescribed by the local health authorities.

5.11 Assessment of Efficacy of IDA on STH (including *Strongyloides* by qPCR*)*

Expected number of positive individuals per treatment arm: We assume that per treatment arm we will collect stool samples from a community of about 1,000 individuals. With a

confidence level of 95% and a confidence interval of 5% we would require testing at least 278 participants. Assuming a sample size of 300 (~278) and a confidence level of 95% we expect to treat at a prevalence of 50% (ie any STH) between 133 and 167 infected individuals and at a prevalence of 10% (ie one STH species only) between 20-40 infected individuals. Based on previous data from eastern Indonesia and PNG we expect a minimum prevalence of any STH of 50%.

Study sites: It is sufficient to select the 300 individuals per treatment arm from one study area/village, because a more homogeneous distribution of STH compared to LF can be expected. Susceptibility to the drug treatment should not vary within the same region.

Collection of stool samples: We will follow the DOLF '*SOP for Stool Collection*'. A convenience sampling method will be used without special regard to sex and age, because drug efficacy is unlikely to depend on these parameters. A follow-up stool sample will be collected 2-4 weeks and one year after treatment from the same individuals that provided the baseline sample. We expect a compliance of 90% because of pre-selection of compliant individuals. Participants who are FTS positive one year after treatment will also be asked to provide a stool sample.

Assessment of STH eggs: We will use the Kato Katz method before and after treatment, because of its sufficient sensitivity in high prevalence areas, its simple performance in the field, and the standardized quantitative assessment. We will follow the DOLF '*SOP Kato Katz Procedure'.* Stool aliquots will be preserved for later examination by qPCR. This will enable us to archive STH DNA samples before and after IDA treatment and to test for efficacy of IDA for *Strongyloides*. We will follow the DOLF 'SOP Stool Sample Management ' (version 2016). Participants will be informed that that the stool samples may be shipped to other countries like the United States for qPCR testing. These samples may be stored for a longer time after the intended testing. No HIV or human genetic testing will be performed.

5.12 Randomization

Communities will be assigned treatment either by randomization or by purposively matching communities based on population and prevalence of LF. If the prevalence is homogenous across the communities, each site may be randomly assigned to one of the two treatment arms. If the prevalence is heterogeneous, communities will be selected into each arm so that the population and prevalence between the two treatment arms is similar.

5.13 Withdrawal

Participation in this project is completely voluntary, and participants may terminate participation at any time. Also if the well-being of the participant is compromised in any way, based on the opinion of the investigator, the participant can also be withdrawn from the study. Even if the participant leaves the project early, we will encourage them to contact us at any time within the month after treatment to report any possible study-related AEs.

All particpants that sign the informed consent and receive study drug will be included in the analysis.

5.14 Efficacy and Effectiveness of IDA vs DA

One year post MDA, all individuals who were positive for either microfilaremia or filarial antigenemia (FTS) during the baseline survey will be tested for filarial antigen using the FTS to assess their response to treatment and to compare the efficacy of the two treatment regimens. Persons with positive FTS will also be tested for nocturnal microfilaremia by blood smear (finger prick).

We will also collect stool samples from all treated individuals who were positive for helminth or LF infections in order to describe the long term effect of both treatment regimens on STH.

Additionally, for all participants who were positive for filarial antigenemia, 60µl of capillary blood will be applied to a filter paper. The dried filter disks will be stored in a cool, dry place until used for testing. Study participants will be informed that their blood on filter paper will be tested for stongyloides and potentially for other diseases of public health importance and will be shipped to the United States and stored for a longer time after the intended testing. No HIV or genetic testing will be performed.

5.15 Retreatment

Any individual who tests positive for lymphatic filariasis at 12 months (by microscopy or antigen test) will be re-treated with the standard MDA regimen (single dose of DEC with Albendazole). If triple drug therapy (IDA) is recommended by the WHO or by national regulatory agencies for lymphatic filariasis and if investigators have adequate supply of ivermectin, infected individuals may be offered IDA. This practice is meant to ensure that all participants who participated in the study may get the most beneficial treatment. Pregnant women will not be eligible for re-treatment.

5.16 Guidelines for Stopping the Trial

There are no pre-specified criteria for terminating the study early.

Upon review of the data for the trial, the DSMB will make decisions regarding the continuation of the trial. The final decision to stop the trial is left to the recommendation of the DSMB. If the DSMB recommends discontinuation or modification of the study, the Chair of the DSMB will meet or talk with the DOLF Project Team at the earliest opportunity to review the basis for the recommendation. The study should be stopped if a treatment arm shows a significant increase in unacceptable side effects that would include, death, fever, and nausea that persist more than a day and would require hospitalization.

5.17 Triple Drug Regimen Acceptability

A survey to assess the treatment acceptability in the community is planned to follow the safety trial (Appendix 7). The overall aim is to understand the community's acceptance of the 3-drug regimen as well as gain insight into the feasibility of administering this new therapy in the future. Part of the investigation will include assessing community member's perception of the possible side effects experienced as a result of the 3-drug therapy compared to the 2-drug therapy, and how that might affect future rounds of mass drug administration (MDA) at the community level.

Community acceptance will be measured using a survey to community members receiving both the 2-drug and 3-drug treatments during the safety trial. The survey participants will be identified from the roster of individuals enrolled in the safety trial. To complement this survey, a series of focus group discussions in the community as well as key informant interviews are proposed with community leaders, health personnel and drug distributors in the same communities to assess perceptions about the 3-drug versus the 2-drug regimen. The community acceptability study will be carried out within one month of the completion of the safety trial. The protocol for the acceptability survey is included in Appendix 7 of this protocol. The community questionnaire and topic guides will be submitted to the the EC for approval as an amendment prior to implementation of the survey.

6 INVESTIGATIONAL PRODUCT

Each of the drugs used in this study is approved for human use and has a prior history of use in the treatment of Lymphatic Filariasis.

6.1 Study Drug Background

Albendazole (ALB) has been known to cause degenerative alterations in the tegument and intestinal cells of the worm by binding to the colchicine-sensitive site of tubulin, thus inhibiting its polymerization or assembly into microtubules [12]. The loss of cytoplasmic microtubules leads to impaired uptake of glucose by larval and adult stages of the parasite, and depletes glycogen stores. Degenerative changes in endoplasmic reticulum and mitochondria of the germinal layer, and the subsequent release of lysosomal enzymes result in decreased production of adenosine triphosphate, which is the source of energy required for survival of the helminth. Due to diminished energy production, the parasite is immobilized and eventually dies. Adverse events are uncommon in persons who are treated with a single dose of albendazole (apart from AEs that result from parasite death). Some patients report mild gastrointestinal AEs such as nausea after ingesting the tablet.

Ivermectin (IVM) is an avermectin compound of macrocyclic lactones derived from the bacterium *Streptomyces avermitilis* [13]. The mechanism by which IVM kills LF microfilariae is not known with certainty, but the drug interferes with glutamate gated ion channels that can affect parasite contractility and release of immunomodulatory molecules by the parasite [13]. IVM also has a direct effect on the central nervous system and muscle function of worms as it enhances strength of inhibitory neurotransmission pathways. The main concern with the use of IVM in animals and humans is neurotoxicity, which can be manifest as ataxia. Neurotoxicity has not been observed in humans given single dose IVM for LF or other parasitic infections [14]. IVM has been used to treat millions of people with LF and onchocerciasis. Peak IVM serum concentrations are reached approximately 4-5 hours after administration. The half-life of IVM in various populations ranges from 12 to 56 hours [15]. There is no evidence of drug: drug interaction between ALB and IVM [16]. IVM can cause nausea, dizziness and occasionally pruritus, but these are infrequent, transient and usually mild. Serious adverse events have occurred in patients with heavy *Loa loa* infections.

DEC (diethylcarbamazine citrate) is an anthelminthic drug that is structurally distinct from ALB and IVM [17]. DEC inhibits arachidonic acid metabolism by LF, and inducible nitric oxide synthase and the cyclooxygenase pathway may be essential for activity *in vivo* [17]. DEC also has anti-inflammatory properties. The mechanisms of action of DEC remain poorly understood. Its ability to kill MF and adult worm depends on the host immune responses since the drug has little direct activity on parasites in vitro. The drug has potent activity against LF microfilaria. DEC has about 50-70% efficacy in killing or sterilization of adult worms [19]. The drug is rapidly absorbed from the gastrointestinal tract, has a serum half-life of 12 to 14 hours, and is excreted in the urine with

little modification by liver metabolism. Adverse events from DEC are unusual apart from those that result from killing filarial worms.

6.1.1 Product Supply and Storage

Only WHO approved drugs will be used in this study. DEC and albendazole will be provided by WHO, and a request will be submitted to Merck to provide ivermectin. Alternatively WHO approved generic ivermectin may be purchased.

All three study drugs are approved and distributed globally by WHO as part of GPELF. Detailed information for each drug is available from the pharmaceutical manufacturer. All products should be maintained between18-25 °C.

7 STUDY PROCEDURES/EVALUATIONS/SCHEDULE

7.1 Triple Drug Therapy (IDA) and Two-Drug Therapy (DA)

The triple-drug combination will consist of a single dose of ivermectin (200 µg /kg), DEC (6mg/kg) and albendazole (flat dose of 400 mg). The two-drug combination will consist of a single dose of DEC (6mg/kg) and albendazole (flat dose of 400 mg). Study personnel will directly observe oral administration of drugs. Drugs will be given after the informed consent has been obtained. The study population will be encouraged to eat before swallowing the medicine (without chewing the tablets) with a glass of water. Vomited doses will be replaced. Drug administration will be supervised (directly observed treatment or DOT) to ensure that all enrolled individuals swallow the drugs. There will be one supervisor per study team.

Universal precautions for individuals collecting and working with blood samples to include proper disposal of contaminated materials (test strips, lancets, capillary tubes, blood film slides) will be in accordance with the guidelines prescribed by the local health authorities.

7.2 Overall Study Schedule

A flow diagram illustrating the study events schedule is presented in Appendix 1.

8 SAFETY REPORTING AND SAFETY MONITORING

The post-treatment assessment team will be composed of individuals with basic medical training who are able to perform a medical history and a basic physical examination (Physicians, local health workers, nursing and/or medical students). Physicians from the area will be available to assist in the evaluation and management of adverse events.

8.1 Definitions

Adverse Event (AE)

Any untoward medical occurrence in a clinical investigation participant who has received a study product intervention and that does not necessarily have to have a causal relationship with the study product. An AE can, therefore, be any unfavorable and unintended sign (including an abnormal laboratory finding, for example), symptom, or disease temporally associated with the use of a study medicinal product, whether or not considered related to the study medicinal product.

An AE does not include:

- Medical or surgical procedures (e.g. surgery, tooth extraction, transfusion). The condition that leads to the procedure is an adverse event
- Pre-existing diseases or conditions or laboratory abnormalities present or detected prior to the screening visit that do not worsen

Serious Adverse Event (SAE)

An SAE is any adverse event that results in any of the following outcomes:

- Death;
- Life-threatening (immediate risk of death);
- Inpatient hospitalization or prolongation of existing hospitalization;
- Persistent or significant disability or incapacity;
- Congenital anomaly/birth defect;
- Important medical events that may not result in death, be life threatening, or require hospitalization may be considered a serious adverse event when, based upon appropriate medical judgment, they may jeopardize the participant and may require medical or surgical intervention to prevent one of the outcomes listed in this definition. Examples of such medical events include allergic bronchospasm requiring intensive treatment in an emergency room or at home, blood dyscrasias or convulsions that do not result in inpatient hospitalization, or the development of drug dependency or drug abuse.

Unexpected

An adverse reaction, the nature or severity of which is not consistent with the applicable product information (e.g., Package Insert).

Expedited Safety Report

Documentation in appropriate form and format summarizing an SAE that meets expedited safety reporting criteria, submitted within the required reporting time frame of applicable regulatory authorities and/or IRBs/IECs of participating countries.

8.2 Assessment of Adverse Events

Adverse event monitoring will be performed approximately 24 and 48 hours following drug administration (late afternoon and evenings following treatment, house visit #3 and 4, Appendix 1). All dosed participants will be followed for adverse events through Day 7.

Evaluations will be documented on pre-printed Patient Monitoring forms (Appendix 3) using the scoring instructions for AEs (Appendix 4) or entered directly into an electronic form using tablet computers.

Most adverse events after mass drug administration are associated with killing of MF and are seen in the first 12-24h following treatment. However, occasional adverse events related to adult worm death may be delayed by several days.

To capture these adverse events and to assure that any systemic adverse events that occurred earlier have resolved, study personnel will also visit study villages daily on days 3 through 7 after treatment (passive AE monitoring). Individuals with AEs that interfere with activities of daily living (grade 2 or higher) will have more detailed assessments that will include a brief physical examination (including measurement of temperature, blood pressure and pulse).

8.2.1 Serious Adverse Event (SAE) Assessment and Management

Study participants with definite or suspected serious AEs (any event ≥ grade 3) will be referred to a physician or appropriate health care professional for evaluation. These evaluations will be documented with special adverse event evaluation forms (Appendix 5), following the instructions (Appendix 5a).

An SAE may qualify for reporting to regulatory authorities if the SAE is possibly attributable to one or more of the study drugs, and is unexpected based on the Company Core Safety Information.

The investigator should notify the Institutional Review Board (IRB) or Ethics Committee (EC) as soon as is practical, of serious events in writing where this is required by local regulatory authorities, and in accordance with the local institutional policy.

8.3 Reporting of Pregnancy

Pregnancy is an exclusion criteria for this study. Although not AEs, pregnancies are reportable events. The pregnancy outcome (e.g., any premature terminations, elective or therapeutic, and any spontaneous abortions or stillbirths, as well as the health status of the mother and child including date of delivery and infant’s gender and weight) should be reported. Any pregnant woman inadvertently dosed who has a miscarriage or spontaneous abortion within the week of follow-up will be reported as an SAE.

8.4 Safety Monitoring by the Oversight Committee

A Data Safety Monitoring Board consisting of 4 experts (including 3 physicians) knowledgeable in neglected tropical diseases will be in place to monitor the safety data per country and across countries participating in the DOLF project.

9 CLINICAL MANAGEMENT OF EVENTS

Individuals who have basic medical training (physicians and/or nursing or medical students) are who are able to complete and pass a training course will be responsible for the initial adverse event evaluations.

In the case of mild symptomatic reactions local health workers/study personnel will provide antipyretics/analgesics and anti-allergic agents at the time of follow-up. It is anticipated that the majority of adverse events will resolve within a day or two and will not require treatment. In the initial adverse event monitoring if any of the following are noted a physician will be notified to evaluate the participant for a potential serious adverse event:

- Participant reports they are unable to participate in their normal daily activities
- Participant has or reports a temperature >39ºC
- Participant has or reports a significant drop in blood pressure
- Participant has other significant objective findings that should be referred to a physician

All grade 3, 4 or 5 events or overnight hospitalization will require completion of the Adverse Event Evaluation and Report Form (Appendix 5). The physician will provide any required immediate treatment and facilitate admission into the hospital or health centre as deemed appropriate.

9.1 Adverse Event Monitoring and Management

Adverse Event monitoring and management will follow or exceed WHO guidelines. Participants will be visited on the two days following treatment by study personnel with medical training. Formal assessment of adverse events (with a standard form) will take place on days 1 and 2 and later if symptoms persist or start late.

Study personnel will use the toxicity table (Appendix 4) to score adverse events for severity. Serious adverse events will be followed until resolution. Study personnel will visit each study area daily for 7 days following MDA treatment to manage any adverse events as follows:

9.1.1 Mild Localized Symptoms

Participants who develop painful lymphadenopathy, scrotal pain or painful swelling or nodules along lymphatic vessels will be treated with acetaminophen or ibuprofen.

9.1.2 Moderate to Severe Localized Adverse Events

Participants with more severe local adverse effects (Grade 3, Appendix 4) like acute swelling or severe scrotal pain that is not relieved by acetaminophen will be transported by study personnel to the medical facility identified for the study for evaluation by one of the physicians or other qualified medical personnel involved in the study. If appropriate, participants will be transferred (after stabilization) to the Departmental Hospital.

9.1.3 Moderate to Severe Systemic Adverse Events

Participants with more severe systemic adverse effects (fever over 39°C > 72 hours, other adverse events ≥ grade 3, syncope, jaundice, or any condition that might require hospitalization) will be transported by study personnel for physician or other qualified medical personnel for evaluation at the medical facility identified for the study. If appropriate, participants will be transferred (after stabilization) to a local hospital.

9.2 Rapid Response Teams for Management of Adverse Events

Medical teams will be located at strategic places close to the study sites. Participants, and persons involved in the study (inclusion process and AE monitoring) will be informed about the location and phone numbers of these teams so that they can report directly to these teams if necessary. These teams will be in position from the day of drug administration until the completion of operations.

10 STATISTICAL CONSIDERATIONS

All participants receiving study drug will be included in both the safety and efficacy analysis

10.1 Safety

The sample size of 3000 participants per arm in Papua New Guinea will contribute to the total sample size for the project. The WHO requires a total of 10,000 participants to detect a SAE rate of 0.1% for each of the treatment regimens and recruitment in other countries (e.g., India, Indonesia, Haiti, and Sri Lanka) is planned to contribute to the overall sample size required. It is well known that systemic AEs are related to killing of MF and that the severity of AEs is related to MF counts. Since MF rates in the study area are relatively low, the study will not be powered to compare rates of SAEs between MDA regimens.

The primary endpoint for safety studies will be the rates of SAEs that occur in infected and in uninfected participants within the first 7 days post MDA. Total AEs will be a secondary endpoint for the study.

10.2 Efficacy

Assuming an MF-prevalence of 1% in the study population at baseline, the survey is expected to detect at least 30 MF positive participants in each arm. A minimum of 21 (70%) of these MF-positive participants in each arm will be retested at 12 months post-treatment for antigenemia and microfilaremia. This sample size is adequate to demonstrate superiority of the IDA regimen (assumptions: 90% reduction in MF prevalence after IDA and 60% reduction after DA, 80% power for detecting an effect size of 30%). The primary endpoint for efficacy will be complete clearance of MF 12 months post MDA. Clearance of filarial antigenemia at 12 months will be a secondary endpoint for the efficacy analysis.

10.3 Enrolling Additional Participants

It is possible that recruitment in other countries may be less than anticipated. In this case the number of participants enrolled in this study may need to be increased to make up for the loss in another country. The number of additional people enrolled will be no more than is necessary to reach the total of 10,000 participants treated with IDA. In this situation the pricinple investigators will seek an amendment from the ethics review committees for the expanded enrollment.

11 DATA HANDLING/RECORD KEEPING/SOURCE DOCUMENTS

Data will be collected using a tablet based system, pre-loaded with study templates. Field teams will be trained in the use of the instruments and data will be uploaded as entries are completed.

11.1 Types of Data Collected

Enrollment Data will include (Appendix 2):

- Site Identification
- Participant Identifier
- Informed Consent Date
- Demographic Information
- Pregnancy/last menstrual period
- Medical History
- Presence of hydrocele and lymphedema
- Bed Net and Window Screen Use
- History of prior MDA tratement
- Pre-treatment adverse event assessment
- Limited Physical Exam

Laboratory Results

- FTS (filarial antigen test)
- FTS score
- MF slide (including MF count)

Participant Monitoring Forms (24 & 48 hour post treatment):

- Adverse Event Assessment
- Physical Examination, as appropriate

Adverse Event Evaluation and Report (Appendix 5)

- Participant Identification
- MDA Treatment
- Concomitant Medication taken at the time of the MDA
- AE Description,
- Start and Stop Date
- Outcome
- SAE Evaluation and causality to MDA (definite, probable, possible, or unrelated)

11.2 Study Records Retention

Study documents will be retained for a minimum of three (3) years after the last participant has completed the study. These documents will be retained for a longer period, however, if required by local regulations. No record will be destroyed without the written consent of DOLF.

Each participating site will maintain appropriate medical and research records for this trial, in compliance with ICH E6, Section 4.9, regulatory and institutional requirements for the protection of confidentiality of particpants. Each site participating in this study will permit authorized representatives of the sponsor and regulatory agencies to examine (and when required by applicable law, copy) clinical records for the purposes of clinical site monitoring, quality assurance reviews, audits, and evaluation of the study safety and progress.

11.3 Source Documents

This study will use both paper and electronic source and this may vary by location due to local availability. All sites will be provided with hard copy data collection forms derived from the eCRFs. If data is first entered on paper the study staff will enter the data into the electronic capture system.

12 RESPONSIBILITIES

12.1 Investigator Responsibilities

12.1.1 Good Clinical Practice

The investigator will ensure that the basic principles of Good Clinical Practice are followed along with the appropriate laws and regulations of the country in which the research is conducted.

12.2 Institutional Review Board (IRB)/Ethics Committee (EC)

The protocol and any accompanying material to be provided to the participants such as the informed consent will be submitted to the EC for review and approval. Approval from the committee must be obtained before starting the study and should be documented in correspondence to the investigator.

Any modifications to the protocol after receipt of the IRB or EC approval must be submitted to the committee for approval prior to implementation.

12.3 Informed Consent

It is the responsibility of the investigator to obtain written informed consent from each individual participating in the study after adequate explanation of the aims, methods, objectives and potential risk of any study related procedures. The investigator must use an IRB/EC approved informed consent. The investigators will accept either signed (cursive) or printed signatures or a witnessed mark in the case of illiterate study participants on the consent form.

Only the principal investigators or study staff authorized to obtain consent will consent participants for this study. Only individuals who have signed the consent form and meet eligibility criteria will be enrolled in the study.

Entry into the study and participation will be strictly voluntary. It will be made clear that refusal to participate or a decision to withdraw can occur at any time throughout the course of the study and will not influence their rights or the care they receive at local health facilities. Potential participants will be told that all of their health information will be confidential and that records will be coded without personal identifiers before they are shared with statisticians or project scientists outside of the village/region/country. They will also be told that no monetary or other gains are offered in exchange for participation apart from compensation for time and reimbursement of travel expenses as described above.

12.3.1 Informed Consent Training

Each step of the study will be explained in detail to the local study personnel. The basic principles of informed consent process, documentation of informed consent, protection of participants' rights, confidentiality, and handling of data will be covered in these training sessions. Study personnel will be monitored by the on-site project coordinatoron a regular basis to ensure compliance with the principles of informed consent. The investigators and study

personnel who will obtain consent from study participants will also receive training in the informed consent process and good clinical practices (GCP).

12.3.2 Country Specific ICF Information

Professionals, PIs and site project coordinators will conduct on-site training sessions for study staff who will be collecting study information, specimens, and/obtaining consent from participants in the study. The study will be explained in detail to the local study staff. The basic principles of informed consent process, documentation of informed consent, protection of participants' rights, confidentiality, and handling of data will be covered in these training sessions. All training sessions will be documented, and study staff monitored by the on-site project coordinator on a regular basis to ensure compliance with the principles of informed consent. The Principal Investigator will provide training and readings materials on human participant regulations with an emphasis on informed consent. If the field staff in PNG has difficulty with the use of modern technology (computers, mouse, etc.) the Principal Investigators and/or professional staff will provide the specified training (as outlined in the protocol) and submit a signed attestation for the informed consent process training. The investigators will accept either signed (cursive) or printed signatures or a witnessed mark in the case of illiterate study participants on the consent form.

Only the principal investigators and study staff authorized and trained to obtain consent will consent participants for this study. Only individuals who have signed the consent form and meet eligibility criteria will be enrolled in the study.

12.4 Participant Privacy

Privacy of the study participants will be maintained by assigning study participants a unique study identification number (UNID). All data, blood samples and laboratory results will be recorded and analyzed by UNID with no personal identifiers. All information collected, including demographic information about enrolled participants will be kept confidential and available only to the investigators and authorized study personnel such as the data manager.

Though most data will be collected on tablets, all written forms (i.e., consent and any paper data collection forms) will be stored in a designated locked area with limited access. All forms will be labelled and filed in cabinets with the study protocol umber, PI's names and collection dates. These cabinets will be metal and have functioning locks. Keys will be kept with the Project Coordinator. All electronic devices on which data are entered will be password protected. PIs and/or Project Coordinatorr will authorize access. The paper forms will be stored for the duration of the study plus three years per IRB protocol for primary data storage.

12.5 Data Ownership

The data are the property of PNGIMR. The Principal Investigators, Co-investigators and key personnel may use the results of this study for publications, presentations at scientific meetings or as preliminary data for subsequent grant applications. Confidentiality of study participants will be maintained by not using names or personal identifiers. PNGIMR will provide de-identified data from the study to DOLF for use in publications and presentations that present results across different study sites. At least one Papua New Guinea based researcher will be included as an

author for any publications with data from Papua New Guinea.

The study site Project Coordinator will permit access to all documents and records that may require inspection by the funding agencies, governmental regulatory agencies, institutional review boards or its authorized representatives.

13 PUBLICATION POLICY

Manuscripts should be submitted for publication no later than one year following the date of the "last patient/last visit". This study includes follow-up data collection past the primary end point, including acceptability and efficacy results. It is not necessary to wait for the follow-up studies to be completed in order to publish the primary safety data.

Endemic country investigators have an obligation to publish the results of DOLF studies conducted in their country. These results benefit the national NTD programs and the citizens of the country where the study was completed. DOLF collaborating institutions are willing to help their endemic country partners with the data analysis, manuscript preparation, publication fees, etc. However, the lead author should be an investigator from the country where the study was performed.

DOLF scientists will be responsible for publishing the results from the aggregated data that combines the results from multiple study sites. The purpose of these manuscripts is to consider the similarities and differences in results obtained in different countries. These publications will not include as much detailed data or analyses as the country specific publications. Publications that report multi-country results will have at least one co-author from each country included in the manuscript.

14 LITERATURE REFERENCES

1. Hooper PJ, Bradley MH, Biswas G, Ottesen EA. The Global Programme to Eliminate Lymphatic Filariasis: health impact during its first 8 years (2000-2007). Annals of tropical medicine and parasitology. 2009;103 Suppl 1:S17-21.

2. Hooper PJ, Chu BK, Mikhailov A, Ottesen EA, Bradley M. Assessing progress in reducing the at-risk population after 13 years of the global programme to eliminate lymphatic filariasis. PLoS neglected tropical diseases. 2014;8(11):e3333.

3. Thomsen EK, Sanuku N, Baea M, Satofan S, Maki E, Lombore B, et al. Efficacy, Safety, and Pharmacokinetics of Coadministered Diethylcarbamazine, Albendazole, and Ivermectin for Treatment of Bancroftian Filariasis. Clin Infect Dis. 2016;62(3):334-41.

4. Romani L, Whitfeld MJ, Koroivueta J, Kama M, Wand H, Tikoduadua L, et al. Mass Drug Administration for Scabies Control in a Population with Endemic Disease. The New England journal of medicine. 2015;373(24):2305-13.

5. Graves PM, Makita L, Susapu M, Brady MA, Melrose W, Capuano C, et al. Lymphatic filariasis in Papua New Guinea: distribution at district level and impact of mass drug administration, 1980 to 2011. Parasites & vectors. 2013;6:7.

6. Bockarie MJ, Tavul L, Ibam I, Kastens W, Hazlett F, Tisch DJ, et al. Efficacy of single-dose diethylcarbamazine compared with diethylcarbamazine combined with albendazole against Wuchereria bancrofti infection in Papua New Guinea. Am J Trop Med Hyg. 2007;76(1):62-6.

7. Bockarie MJ, Tisch DJ, Kastens W, Alexander ND, Dimber Z, Bockarie F, et al. Mass treatment to eliminate filariasis in Papua New Guinea. The New England journal of medicine. 2002;347(23):1841-8.

8. Weil GJ, Kastens W, Susapu M, Laney SJ, Williams SA, King CL, et al. The impact of repeated rounds of mass drug administration with diethylcarbamazine plus albendazole on bancroftian filariasis in Papua New Guinea. PLoS neglected tropical diseases. 2008;2(12):e344.

9. Yahathugoda TC, Supali T, Rao RU, Djuardi Y, Stefani D, Pical F, et al. A comparison of two tests for filarial antigenemia in areas in Sri Lanka and Indonesia with low-level persistence of lymphatic filariasis following mass drug administration. Parasites & vectors. 2015;8:369.

10. Weil GJ, Ramzy RM. Diagnostic tools for filariasis elimination programs. Trends in parasitology. 2007;23(2):78-82.

11. Thomsen, E.K., et al., Efficacy, Safety, and Pharmacokinetics of Coadministered Diethylcarbamazine, Albendazole, and Ivermectin for Treatment of Bancroftian Filariasis. Clin Infect Dis, 2015.
12. Horton, J., Albendazole: a review of anthelmintic efficacy and safety in humans. Parasitology, 2000. **121 Suppl**: p. S113-32.
13. Goa, K.L., D. McTavish, and S.P. Clissold, Ivermectin. A review of its antifilarial activity, pharmacokinetic properties and clinical efficacy in onchocerciasis. Drugs, 1991. **42**(4): p. 640-58.
14. Edwards, G., Ivermectin: does P-glycoprotein play a role in neurotoxicity? Filaria J, 2003. **2 Suppl 1**: p. S8.
15. Ottesen, E.A. and W.C. Campbell, Ivermectin in human medicine. J Antimicrob Chemother, 1994. **34**(2): p. 195-203.
16. Awadzi, K., et al., The co-administration of ivermectin and albendazole--safety, pharmacokinetics and efficacy against Onchocerca volvulus. Ann Trop Med Parasitol, 2003. **97**(2): p. 165-78.
17. Ottesen, E.A., Efficacy of diethylcarbamazine in eradicating infection with lymphatic-dwelling filariae in humans. Rev Infect Dis, 1985. **7**(3): p. 341-56.
18. Bockarie, M.J., et al., Randomised community-based trial of annual single-dose diethylcarbamazine with or without ivermectin against Wuchereria bancrofti infection in human beings and mosquitoes. Lancet, 1998. **351**(9097): p. 162-8.
19. Noroes, J., et al., *Assessment of the efficacy of diethylcarbamazine on adult Wuchereria bancrofti in vivo.* Trans R Soc Trop Med Hyg, 1997. **91**(1): p. 78-81.

LIST OF APPENDICES

Appendix 1: Study Drug Flow Diagram (country specific)
Appendix 2: Participant Enrollment Form [Example]
Appendix 3: Participant Monitoring Form Example
Appendix 4: Guide to Assigning Adverse Event Severity
Appendix 5: Adverse Event Evaluation and Report Form (AEERF) [Example]
Appendix 5a: Required Reporting & Guidelines for SAE(s)
Appendix 6: Informed Consent Form [Example]
Appendix 7 Treatment Acceptability Study Protocol

APPENDIX 1: STUDY FLOW DIAGRAM (COUNTRY SPECIFIC)

AE Adverse Events
FTS Filarial Test Strip detects LF Cir. Antigen
IRB Institutional Review Board
KAP Knowledge Attitudes and Practices
LF Lymphatic filariasis
MDA Mass Drug Administration
Mf Microfilaria
SOP Standard Operating Procedures

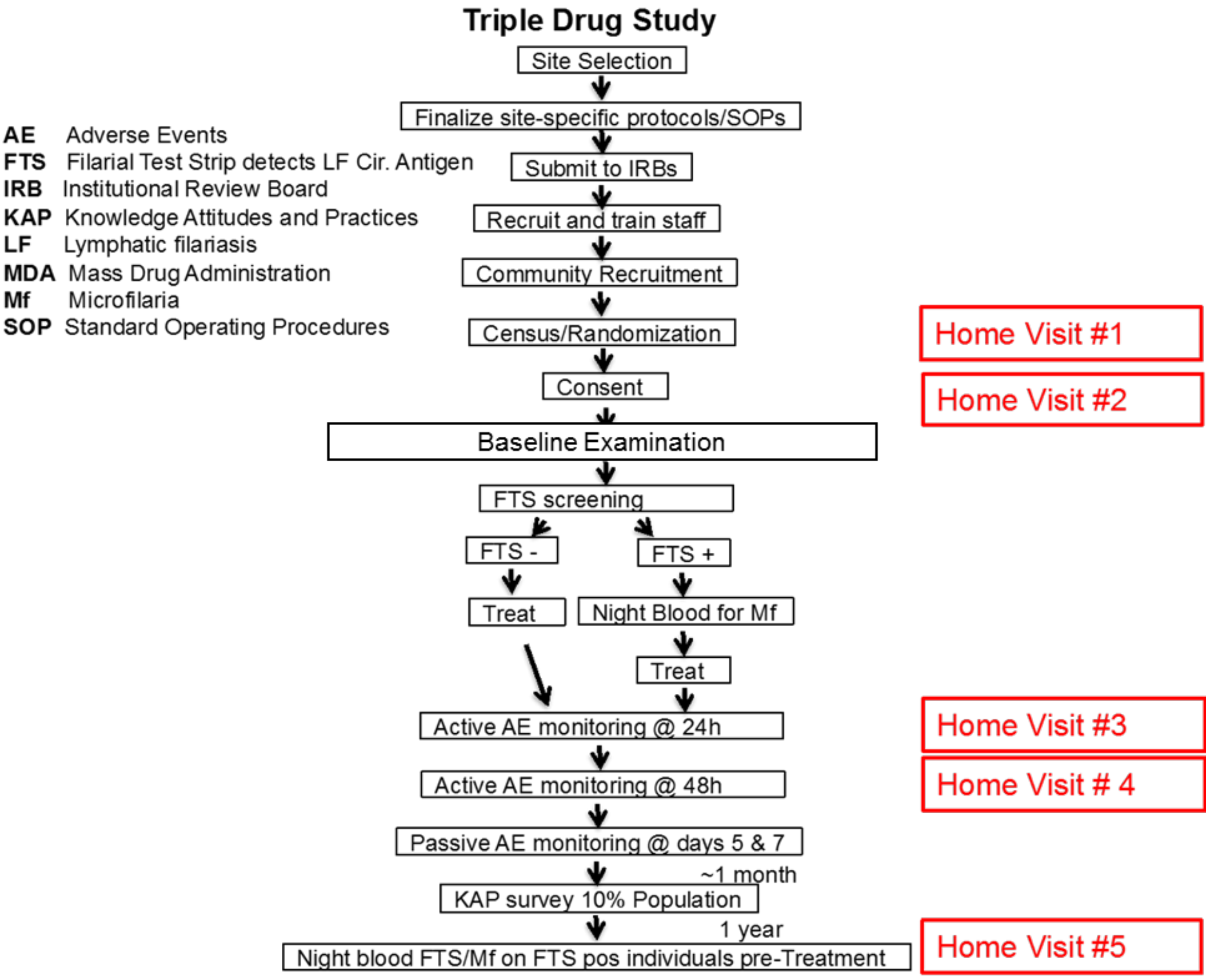

APPENDIX 2: PARTICIPANT ENROLLMENT FORM [EXAMPLE]

Participant ID (Barcode):

---

1. SITE INFORMATION

Team (**required**):	Data Entry Clerk ID (**required**):
Enrollment Date (**required**) (DD-MM-YYYY):	
Consent Method (**required**)<br>☐Self<br>☐Parent<br>☐Other guardian (specify):<br>☐Teacher<br>☐No consent (<u>STOP</u>)	Enrollment Location<br>☐Home<br>☐School<br>☐Village meeting point
Enrollment Village:	

2. PARTICIPANT INFORMATION AND MEDICAL HISTORY

Gender: ☐M ☐F	*Note: if exact date is not known, birth year is sufficient*			Age (Years):
	Birth Day (DD):	Birth Month (MM):	Birth Year (YYYY):	
Village of Residence:		House Number:		
Participant ID (Barcode):<br>(affix barcode at the top of each form AND write in ID number at top of each page)				
Females only: **When was the date of your last menstrual period? (*read options)***<br>☐Definitely less than 4 weeks ago<br>☐Post-menopause<br>☐4 weeks of longer (<u>STOP</u>)<br>☐Uncertain (<u>STOP</u>)				
Males only: **Do you have swelling or enlargement of your scrotum?** ☐Yes ☐No				
Males only: **Do you feel pain in your testicles or scrotum?** ☐Yes ☐No				
**Do you have any of the following chronic medical conditions? (*read options*)**<br>☐Hypertension (high blood pressure)<br>☐Asthma or chronic lung disease<br>☐Chronic kidney disease (renal insufficiency)<br>☐Diabetes<br>☐Other (specify):				
**Do you have swelling in your arms or legs (lymphedema)?** ☐Yes ☐No				
If participant reports lymphedema exam them and confirm presence of edema.<br>Left arm ☐No edema ☐Yes edema				

Appendix 2: PARTICIPANT ENROLLMENT FORM [Example]

Participant ID (Barcode):

Left leg	☐No edema	☐Yes edema
Right arm	☐No edema	☐Yes edema
Right leg	☐No edema	☐Yes edema

Did you use a bed net last night? ☐Yes ☐No

Does your house have screens on the windows? ☐Yes ☐No

Do you spray indoors to prevent mosquitos? ☐Yes ☐No

Did you swallow medicines during MDA treatment for filariasis in the last twelve months? (If YES enter the date)

☐Yes Date of last MDA (MM-YYYY):____________ ☐Do not remember date

☐No

☐Uncertain

☐N/A—no MDA distributed

Have you ever taken the following medication called…?

**Albendazole?**	**Ivermectin?**	**DEC?**
☐Yes ☐No ☐Don't know	☐Yes ☐No ☐Don't know	☐Yes ☐No ☐Don't know
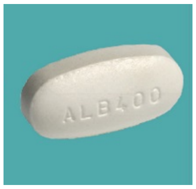	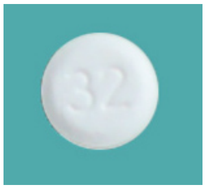	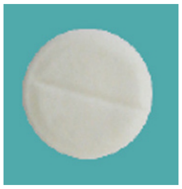

3. AE ASSESSMENT

Today, were you too sick to work or go to school?

☐Yes (**exclude, but finish the pre-treatment assessment**)

☐No

EXPLAIN: ***Now I will ask you some questions about your health during the past two days.***

In the past 2 days have you experienced…?

..Fever	☐Yes	☐No
..Dizziness, giddiness, or fainting	☐Yes	☐No
..Confusion	☐Yes	☐No
..Drowsiness	☐Yes	☐No
..Headache	☐Yes	☐No
..Cough	☐Yes	☐No
..Difficulty breathing (wheezing or dyspnea)	☐Yes	☐No
..Nausea	☐Yes	☐No
..Vomiting	☐Yes	☐No
..Diarrhea	☐Yes	☐No
..Stomach pain	☐Yes	☐No

Appendix 2: PARTICIPANT ENROLLMENT FORM [Example]

Participant ID (Barcode):

..Unusual swelling, beyond baseline lymphedema ☐Yes ☐No<br>Specify Location: ☐Arm ☐Leg ☐Breast ☐Scrotum<br>..Joint or muscle pain ☐Yes ☐No<br>..Weakness ☐Yes ☐No<br>..Swelling or pain in your armpit or groin ☐Yes ☐No<br>..Men only: pain in you testicles or scrotum ☐Yes ☐No<br>..Itchy skin ☐Yes ☐No<br>..Rash (specify location):____ ☐Yes ☐No<br>Is the rash in the spaces between your fingers? ☐Yes ☐No<br>..Other illness or symptoms (specify):____ ☐Yes ☐No
**Have you ever suffered from scabies?** (*note: use local name for scabies*)<br>☐Yes<br>☐No<br>☐Don't know
Additional notes or comments:

4. EXAMINATION

Team (**required**):	Clinician (**required**):
Data Entry Clerk ID (**required**):	

Measurements	Values / status
Height (cm)	
Weight (kg)	
BMI (calculated)	
Scabies	☐Yes ☐No
If Yes, please take photograph	

APPENDIX 3: PARTICIPANT MONITORING FORM [EXAMPLE]

FOR: Day 1 & 2, if needed days 3-7

Participant ID (Barcode):

Use this form for active monitoring of adverse events on day 1 (24 hours) and 2 (48 hours) following therapy, as well as for recording symptoms reported by those presenting with complaints on days 3-7 post-treatment.

Team (**required**):	Clinician (**required**):
Data Entry Clerk ID (**required**):	

1. PARTICIPANT INFORMATION

Gender: ☐M ☐F	Age (Years):	Village of Residence:
Treatment Village:		Treatment Date (DD-MM-YYYY):

2. ASSESSMENT INFORMATION

*Day 1 and 2: **All** participants should be asked **all** the questions in Table 1.*

*Days 3-7: Any participant who presents with a complaint should be asked **all** the questions in Table 1*

Table 1: Reported Symptoms

- *Record a symptom grade from 0-5 for each day on which the participant experienced symptoms.*
- *For participants reporting ANY symptom, complete every questions in Table 1.*
- *Refer to the Appendix 4 for symptom-specific scoring criteria.*
- *Anyone with a symptom typed in **bold** needs to have Table 2 completed.*

Symptom Grading
0 = No adverse event or within normal limits
1 = Mild adverse event, does not interfere with work or school
2 = Moderate adverse event, interferes with work or school at least 1 day
3 = Severe and undesirable adverse event; interferes with ADL, requires medical assessment
4 = Potentially life-threatening or disabling adverse event; requires transfer to medical facility
5 = Death

Symptoms/Signs	**Post-treatment day(s) on which symptoms or signs were present**						
**Since you took the tablets have you experienced…?**	**Day 1**	**Day 2**	**Day 3**	**Day 4**	**Day 5**	**Day 6**	**Day 7**
..**Fever**							
..**Dizziness, giddiness, or fainting**							
..**Confusion**							
..**Drowsiness**							

Appendix 3: PARTICIPANT MONITORING FORM [Example]

FOR: Day 1 & 2, if needed days 3-7

Participant ID (Barcode): ____________________

..Headache							
..Cough							
..**Difficulty breathing (wheezing or dyspnea)**							
..Nausea							
..Vomiting							
..Diarrhea							
..Stomach pain							
..Unusual swelling, beyond baseline lymphedema (specify location below)							
Arm							
Leg							
Breast							
Scrotum							
..Joint or muscle pain							
..Weakness							
..**Swelling or pain in your armpit or groin**							
..Men only: pain in your testicles or scrotum							
..Itching skin							
..**Rash (specify location and brief description):**							
..Other illness or symptoms (specify):							

If there is any symptom grade ≥ 3, you must notify the supervising medical officer and the participant must be evaluated by the medical team.

Appendix 3: PARTICIPANT MONITORING FORM [Example]

FOR: Day 1 & 2, if needed days 3-7

Participant ID (Barcode):

**Table 2: Physical Examination**							
• *You must complete this table for any participant reporting any* ***bolded*** *symptom in Table 1 OR for any symptom* ***grade ≥2***<br>• *Record the result under the column that corresponds to the day the assessment was taken.*							
	**Post-treatment day(s)**						
**Measurements**	**Day 1**	**Day 2**	**Day 3**	**Day 4**	**Day 5**	**Day 6**	**Day 7**
Height (cm)							
Weight (kg)							
BMI (calculated)							
Temperature							
Blood pressure, sitting							
Blood pressure, lying down (*measure only if sitting systolic BP <100)*							
**Post-Exam Adverse Event Grade** (Assign grade of 0-5 for the adverse reactions below based on physical exam. See Appendix 4 under "post-exam assessment" for specific grading criteria)							
Allergic reaction							
Hypotension (low blood pressure)							
Lymphangitis (streaks of redness, warmth, and swelling in arms or legs)							

APPENDIX 4: GUIDE TO ASSIGNING ADVERSE EVENT SEVERITY

(G*rade 0 = no symptoms; grade 5 = death from adverse event*)

Symptoms/Signs	Grades			
	**1. Mild**	**2. Moderate**	**3. Severe**	**4. Life-threatening**
Fever (non-axillary temperatures only)	38.0 – 39.0ºC	39.1 – 40.0ºC	> 40.0ºC	> 40.0ºC for > 48 hrs
Dizziness, giddiness, or fainting	Mild, not interfering with work or school	Moderate, unable to work or attend school for 1 day, but no fainting	Any loss of consciousness (fainting)	-
Confusion or excess drowsiness*	Mild, not interfering with work or school	Moderate; confusion or drowsiness interfering with ability to work	Confusion, loss of memory, or sleepiness interfering with activities of daily living	Delerium, inability rouse, or coma
Fatigue	Mild, not interfering with work or school	Moderate, unable to work or attend school at least 1 day	Unable to perform activities of daily living, > 1day	Required hospitalization
Headache	Mild pain not interfering with work or school	Moderate pain; pain or analgesics interfering with ability to work or attend school	Severe pain; pain or analgesics interfering with activities of daily living	Disabling, duration > 48 hr
Cough	Mild, relieved by non-prescription medication	Requiring narcotic antitussive	Severe cough or coughing spasms, poorly controlled by treatment	Hospitalization or respiratory failure requiring mechanical ventilation
Difficulty breathing (wheezing or dyspnea)	Mild, not interfering with work or school	Moderate, unable to work or attend school for 1 day	Severe, more than 1 day and required transfer to clinic or hospital	Hospitalization or respiratory failure requiring mechanical ventilation
Nausea	Able to eat	Oral intake significantly decreased	No significant intake, requiring IV fluids	-

Appendix 4: GUIDE TO ASSIGNING ADVERSE EVENT SEVERITY

(G*rade 0 = no symptoms; grade 5 = death from adverse event*)

Symptoms/Signs	Grades			
	**1. Mild**	**2. Moderate**	**3. Severe**	**4. Life-threatening**
Vomiting	1 episode in 24 hours over pretreatment	2-5 episodes in 24 hours over pretreatment	≥ 6 episodes in 24hours, or need for IV fluids (0upatient)	Hemodynamic collapse or overnight hospitalization
Diarrhea	Increase of < 4 stools/day over pre-treatment	Increase of 4-6 stools/ day, or nocturnal stools	Increase of ≥ 7 stools/ day or need for outpatient parenteral support for dehydration	Physiologic consequences wth hemodynamic collapse or requiring hospitalization
Abdominal pain	Mild pain not interfering with work or school	Moderate pain; pain or analgesics interfering with ability to work or attend school	Severe pain; pain or analgesics interfering with activities of daily living	Disabling, duration > 48 hr
Unusual swelling (beyond baseline lymphedema)	Mild, not interfering with work or school	Moderate, unable to work or attend school 1 day	Severe, unable to work/school >1 day	Severe, limiting activities of daily living (unable to walk) > 2 days
Joint or muscle pain	Mild pain not interfering with work or school	Moderate pain; pain or analgesics interfering with ability to work or attend school	Severe pain; pain or analgesics interfering with activities of daily living	Disabling, duration > 48 hr
Swelling or pain in your armpit or groin*	Mild, not interfering with work or school	Moderate, unable to work or attend school 1 day	Severe, unable to work/school >1 day	Severe, limiting activities of daily living (unable to walk) > 2 days
Men only: testicular or scrotal pain	Mild, not interfering with work or school	Moderate, unable to work or attend school 1 day	Severe, unable to work/school >1 day	Severe, limiting activities of daily living (unable to walk) > 2 days
Itching skin	Mild, not interfering with work or school	Moderate, unable to work or attend school 1 day	Severe, unable to work/school >1 day	

Appendix 4: GUIDE TO ASSIGNING ADVERSE EVENT SEVERITY

(G*rade 0 = no symptoms; grade 5 = death from adverse event*)

Symptoms/Signs	Grades			
	**1. Mild**	**2. Moderate**	**3. Severe**	**4. Life-threatening**
Rash	Localized rash (covers only one part of the body)	Diffuse rash (covers multiple parts of the body)	Diffuse rash (covers multiple parts of the body) AND has any blisters or ulcers or mouth sores	Extensive areas with blisters or ulcers OR peeling or blackening of skin
Other illness or symptoms	Mild, not interfering with work or school	Moderate, unable to work or attend school at least 1 day	Unable to perform activities of daily living, > 1day	Required hospitalization

Post-Exam Assessment	Grades			
	**1. Mild**	**2. Moderate**	**3. Severe**	**4. Life-threatening**
Acute allergic reaction	Transient rash, drug Fever <38ºC (<100.4ºF)	Urticaria, drug fever ≥38ºC (≥100.4ºF) and/or asymptomatic bronchospasm	Symptomatic bronchospasm, requiring parenteral medication(s) with or without urticaria	Anaphylaxis with hypotension required hospitalization
Hypotension (low blood pressure)	Changes, but not requiring therapy (including transient orthostatic hypotension)	Requiring brief fluid replacement (such as oral rehydration) but not hospitalization	Requiring i.v. fluids without overnight hospitalization. No sequelae.	Required overnight hospitalization for i.v. fluids, or Shock (acidemia and impaired vital organ function due to tissue hypoperfusion)
Lymphangitis	Mild, not interfering with work or school	Moderate, unable to work or attend school 1 day	Severe, unable to work/school >1 day	Severe, limiting activities of daily living (unable to walk) > 2 days

Note on general aspects of grading

0 = No adverse event or within normal limits
1 = Mild adverse event, does not interfere with work or school
2 = Moderate adverse event, interferes with work or school at least 1 day
3 = Severe and undesirable adverse event; interferes with ADL, requires medical assessment
4 = Potentially life-threatening or disabling adverse event; requires transfer to medical facility
5 = Death

Note: Any event ≥ grade 3 requires a medical evaluation and notification of the medical officer. Any grade 3, 4 or 5 event or overnight hospitalization requires an Adverse Event Evaluation and Report Form.

APPENDIX 5: ADVERSE EVENT EVALUATION AND REPORT FORM (AEERF) [EXAMPLE]

Participant ID (Barcode):

Instructions: Complete this form AFTER completing the Participant Monitoring Form for anyone with symptoms or signs of grade 3 or higher *(unable to perform activities of daily living without assistance for at least one day). The purpose of this form is to provide additional information on more severe adverse events and to assist the medical officer in determining whether a Serious Adverse Event (SAE) has occurred. Please refer to Appendix 5a for definitions.*

Clinician (**required**):

1. PARTICIPANT INFORMATION

**Participant ID (Barcode):**			
Gender: ☐M ☐F	Age:_____Years	Weight:_____Kg	Height:_____cm
Village of Residence:			

2. MDA TREATMENT

Treatment Date (DD-MM-YYY)___________<br>Treatment Village:_______________________<br>Anything irregular about treatment?<br>☐No ☐Yes (specify):	Medications received<br>☐ Albendazole (dose:____mg)<br>☐ DEC (dose:____mg)<br>☐ Ivermectin (dose:____mg)
**Was this the first time you have ever been treated with one of the MDA medications? If No, explain when and circumstances of prior treatment.**<br>Albendazole ☐Yes ☐No (explain):<br>DEC ☐Yes ☐No (explain):<br>Ivermectin ☐Yes ☐No (explain):	

3. OTHER MEDICATIONS AT TIME OF MDA

Please include prescription and non-prescription medications/supplements/herbal remedies taken within 5 days of the MDA. DO NOT include medications used to treat the SAE.

Medication	Indication	Dose and Frequency	Days on which each medication was taken, relative to MDA (if taken the day of MDA, mark “0”; the day before, mark “-1”; the day after, “+1”, and so forth.)
			-5 -4 -3 -2 -1 0 +1 +2 +3 +4 +5 uncertain
			-5 -4 -3 -2 -1 0 +1 +2 +3 +4 +5 uncertain
			-5 -4 -3 -2 -1 0 +1 +2 +3 +4 +5 uncertain

Appendix 5: ADVERSE EVENT EVALUATION AND REPORT FORM (AEERF) [Example]

Participant ID (Barcode):

Medication	Indication	Dose and Frequency	Days on which each medication was taken, relative to MDA (if taken the day of MDA, mark "0"; the day before, mark "-1"; the day after, "+1", and so forth.)
			-5 -4 -3 -2 -1 0 +1 +2 +3 +4 +5 uncertain

4. DESCRIPTION OF THE ADVERSE EVENT

Date of onset (DD-MM-YYYY):	How long after drugs were taken did the event begin?<br>_____hours OR _____days
Clinical signs and symptoms (please describe)<br><br>Do you (the clinician) think this adverse event is/was life-threatening? ☐Yes ☐No	
Was the participant hospitalized? ☐Yes ☐No<br>If yes, indicate<br>1. Date of admission (DD-MM-YYYY):<br>2. Reason for admission:<br>3. Date of discharge (DD-MM-YYYY):<br>4. Clinical course, including drug treatments given to treat adverse event:<br><br>*Attach a copy of any medical records relating to the diagnosis and treatment of the adverse event*	
Laboratory results and diagnostic tests (indicate date, test name, and results):	

Appendix 5: ADVERSE EVENT EVALUATION AND REPORT FORM (AEERF) [Example]

Participant ID (Barcode):

5. ADVERSE EVENT OUTCOME (Check only ONE)

☐Recovering/resolving		
☐Not recovered/not resolved		
☐Recovered/resolved	Date: (DD-MM-YYY)	
☐Recovered/resolved with sequelae	Date:(DD-MM-YYY)	Sequelae:
☐Unknown		
☐Fatal (death)	Date:(DD-MM-YYY)<br>Autopsy: ☐Not done ☐Done (*provide report*) ☐Planned ☐ Status Unknown<br>Death certificate: ☐Provided ☐Requested ☐Not available ☐ Status Unknown	

Appendix 5: ADVERSE EVENT EVALUATION AND REPORT FORM (AEERF) [Example]

Participant ID (Barcode):

---

6. CONCLUSIONS (to be completed by the health-care provider)

Presumptive diagnosis:
Do you think this adverse event was caused by the MDA medications? *Refer to Appendix 5a for detailed explanation of choices.*<br>☐ Definitely<br>☐ Probably (explain):<br>☐ Possibly (explain):<br>☐ Unrelated<br>If "unrelated", what do you believe was the cause of the adverse event?
Does this event meet the criteria for a Serious Adverse Event (SAE)? *Refer to Appendix 5a for detailed definitions of criteria.*<br>☐ Yes, based on the following criteria<br>☐ Death<br>☐ Life-threatening<br>☐ Hospitalization<br>☐ Disability or permanent damage<br>☐ Other serious important medical event: specify<br>☐ No

REPORTER INFORMATION AND SIGNATURES

Investigator Name:	Investigator Signature:	Date:
Reporter Name:	Reporter Signature:	Date:
Reporter's phone number:	Reporter's email address:	

APPENDIX 5a: REQUIRED REPORTING GUIDELINE FOR SERIOUS ADVERSE EVENTS

An Adverse Event Evaluation and Report Form (AEERF) should be completed for every severe adverse event (those scoring grade 3 or higher, see Appendix 4). However, a grade 3 or severe adverse event is NOT the same as a Serious Adverse Events (SAE) and the majority of grade 3 adverse events will not be classified as SAE. The term "severe" is often used to describe the intensity (severity) of a specific event (as in mild, moderate, or severe myocardial infarction); the event itself, however, may be of relatively minor medical significance (such as severe headache). This is not the same as "serious," which is based on patient/event outcome or action criteria usually associated with events that pose a threat to a patient's life or functioning. Seriousness (not severity) serves as a guide for defining regulatory reporting obligations.

The AEERF should guide the medical monitor or health care provider evaluating the patient experiencing a severe AE to determine whether a SAE has occurred. All SAE must be reported promptly. (See Safety Reporting Plan for SAE Reporting Timeline)

Required Reporting

A written report or case report form (CRF—in this study, the AEERF) must be sent from the local physician and local medical monitor by email (scanned records) in the stated timeframes to the Country PI, Global Medical Monitor including the Project PI for the events listed below.

Guidelines for Reporting - Standard Reporting Information

The following information should be included in the initial report/CRF (additional information may be requested):

Minimum Criteria for Reporting

Information for final description and evaluation of a case report may not be available within the required time frames for reporting outlined below. Initial reports should be submitted within the prescribed time as long as the following minimum criteria are met: an ***identifiable patient****; **an identifiable reporting source**; and* ***an event or outcome that can be identified as serious***. Follow-up information should be actively sought and submitted as it becomes available.

Appendix 5a: REQUIRED REPORTING GUIDELINE FOR SERIOUS ADVERSE EVENTS

Complete the following information if available on the initial report and complete a follow-up report as new or additional information becomes available as noted below:

- Description of the event
  Date, time of onset
  Clinical history
  Associated signs and symptoms
  Temporal association with study agent
  Medical management, including rationale
  Pertinent laboratory tests
  Severity – *see definitions or toxicity score*
  Causal relationship to the study drug/vaccine

- Other information
  Relevant past medical history
  Concomitant medications
  Autopsy report or expectation of an autopsy in the case of death

- Outcome of event
  Date, time of resolution, if resolved

- Plans for study participants
  Follow-up
  Treatment of event
  Return to treatment/Contraindicate

- Location/Study Centre

- Reporting Physician

- Verification of notification to IRB and Safety Monitor or DSMB

Definitions

- Adverse Event [Experience] (AE):

  Any untoward medical occurrence, including dosing errors, that may arise during administration of study agent, and which may or may not have a causal relationship with the study agent.

- Unexpected Adverse Event [Experience]:

  Any adverse experience that has not been previously observed (i.e., included in the labelling), whether or not the event is anticipated because of the pharmacologic properties of the study agent.

Appendix 5a: REQUIRED REPORTING GUIDELINE FOR SERIOUS ADVERSE EVENTS

- <u>Serious Adverse Event (SAE):</u>

  Any adverse event occurring at any dose that results in any of the following outcomes:

  a. Death

  b. Life threatening – defined as an experience that places the patient or participant, in the view of the Investigator, at *immediate risk* of death from the reaction as it occurred. (Note; this does not include a reaction that, had it occurred in a more severe form, might have caused death.)

  c. Requires inpatient hospitalization or prolongation of existing hospitalization

  d. Results in a congenital anomaly or birth defect

  e. Results in a persistent or significant disability or incapacity

  f. Important medical events that may not result in death, be life-threatening, or require hospitalization may be considered a serious adverse experience when, based upon appropriate medical judgment, they may jeopardize the patient or participant and may require medical or surgical intervention to prevent one of the outcomes listed in this definition. *(The event might be defined as serious based on progression of grade if Toxicity Tables are being used.)*

Severity

Adverse experience/events should be assessed by the on-site investigator as to their severity and/or intensity.

a. Life threatening

b. Severe: incapacitating with inability to work or do usual activity

c. Moderate: enough discomfort to cause interference with usual activity

d. Mild: awareness of sign or symptom, but easily tolerated

Relationship or Association with Use of Study Agent or Participation in the Study

Appendix 5a: REQUIRED REPORTING GUIDELINE FOR SERIOUS ADVERSE EVENTS

Causal relationship with the investigational study treatment must be assessed by the on-site investigator using the following or similar terms:

- **Definite** – clear-cut temporal association, with a positive re-challenge test or laboratory confirmation.
- **Probable** – clear-cut temporal association, with improvement upon drug withdrawal, and not reasonably explained by the particpant's known clinical state.
- **Possible** – less clear temporal association; other aetiologies are possible.
- **None** – no temporal association with the study drug; related to other aetiologies such as concomitant medications or conditions, or participant's known clinical state.

Other Reporting

Investigators are reminded that they may have other reporting obligations:

- For all studies, there must be compliance with the clinical site Ethics/IRB's policy for reporting adverse events. (As soon as possible for SAEs and as required for AEs.)

APPENDIX 6A: INFORMED CONSENT FORM [EXAMPLE]

Participant ID (Barcode):

This is a research study conducted by the Papua New Guinea Institute of Medical Research (PNGIMR) and Case Western Reserve University in Cleveland, Ohio (USA). It includes only individuals who choose to take part. Please take your time to make your decision. Discuss it with your friends and family.

In this consent form the term “you” may refer to you or your child. You are being asked to take part in this study because you live in an area where you may become sick with lymphatic filariasis. You are being asked to consent for yourself and/or your child or children

WHY IS THIS STUDY BEING DONE?

This study is being done to learn about the side effects people have when they take different kinds of medications to a parasite. The parasite causes hydroceles (bol solap) and lymphedemia (leg solap). This parasite is a small round worm that lives in your body. We call this illness lymphatic filariasis (LF).

We want to collect information about how your body reacts to these medications. We want to know if there is a difference between taking two drugs or three.

HOW MANY PEOPLE WILL TAKE PART IN THE STUDY?

Approximately 6,000 adult men, women and children will take part in this study.

WHAT IS INVOLVED IN THE STUDY?

You will be in this study for one year. You will be put in one of 2 groups at random, similar to flipping a coin. As part of this study one group take the standard two-drug treatment of Diethylcarbamize (DEC) and Albendazole (ALB) and another group will take a three-drug therapy of Diethylcarbamize (DEC), Albendazole (ALB) and Ivermectin (IVM). Before you receive any medication, we will take a small amount of blood from your finger by fingerprick to see if you are infected with LF. If you are infected we will ask you to return at night to get another fingerprick to see if there are worms in your blood. Even if test does not show infection you will be treated with drugs anyway because you may still be infected with LF because the tests can only identify heavy infections.

After you take the medication, a member of the study team will follow up with you 1 to 2 days later too see how you’re are feeling. During these visits the study team will take your temperate, blood pressures and ask how you are feeling. The study team will also return to your village 3 to 7 days to ask if you are not feeling well. If you are not, then we will exam you. If your illness

is severe then the study team will have you see a doctor who can provide treatment. The study team will record information about how you are feeling at each visit.

If you tested positive for LF at the beginning of the study, the study team will collect a small amount of blood from a finger prick 12 months after you're took the medication. If you still have LF, you will be treated again with the standard Papua New Guinea MDA treatment.

After we have finished the study, we will ask some of you about how you felt about the study.

HOW LONG WILL I BE IN THE STUDY?

You will be in this study for one year. We may test your blood for the presence of parasites or what makes your blood strong after six months and 12 months. Some of these tests may be performed in other countries such as the Australia or the United States where these tests are available.

Can I stop being in the study?

Yes. You can decide to stop at any time.

CAN I BE TERMINATED FROM THIS STUDY?

Your participation in this study may be discontinued by the investigators if you move away from the study area during the study.

(For Women) CAN I BE IN THIS STUDY IF I AM PREGNANT?

No, you cannot be in this study if you are pregnant at study enrollment. Being a part of this study while pregnant may expose your unborn child to unknown risks. If you are a woman of childbearing age, the study team will ask you are if you are pregnant.

WHAT ARE THE RISKS OF THE STUDY?

The risk of drawing blood from a finger prick is minimal, although some people become lightheaded after giving blood. You may experience momentary discomfort and/or bruising. Children may be uncomfortable and cry when blood is drawn. You will be watched by members of the research team and given an opportunity to rest if you feel lightheaded. Infection, excess bleeding, clotting, or lightheadedness may occur after a finger prick, but these events are unlikely. If you develop a local infection within 7 days at the site on your arm or finger from where we took the blood, we will provide transportation to your local provincial health facility and a voucher to cover the costs of medications to treat this infection.

When you take these medications, your body may react to the dying worms by developing a fever or you may feel tired or have body aches. This usually means that the drugs are killing worms. When you take three drugs, the medication may kill the worms faster which may lead to more severe side effects.

The following are some possible side effects of the three drugs you will be given, although these side effects are small with a single dose of the drug:

(DEC): – You might experience itching and swelling of face, headache, joint pain, unusual tiredness or weakness. These side effects will pass. Less common side effects you may experience are dizziness, nausea or vomiting. Fever, painful and tender glands in groin, neck armpits or skin rash can occur and usually happens because you are infected with LF.

(ALB): You might experience headache, nausea, stomach pain and vomiting that are usually associated with heavy intestinal helminth (worms in the belly) infections.   There is a very small chance that you might develop rash, hives, itching, difficulty breathing, tightness in the chest, swelling of the mouth, face, lips, or tongue, dark urine.

(IVM): You might experience are diarrhea, dizziness and nausea.   There is a very small chance that you might develop rash; hives; itching; difficulty breathing; tightness in the chest; swelling of the mouth, face, lips, or tongue; eye pain, swelling, or redness; fainting; and fast heartbeat.

If you experience any of these side effects of the drugs you will be treated for them by the health center physicians/staff.

ARE THERE BENEFITS TO TAKING PART IN THE STUDY?

This study will help treat your infection for LF and intestinal worms. The investigators hope that the information learned from this study will benefit people in Papua New Guinea as well as in other areas of the world affected by LF. From this work it may be possible to reduce the number of MDA (mass drug administration) treatments needed to treat the disease.

WHAT OTHER OPTIONS ARE THERE?

You do not have to participate in this study. Taking part in this study is voluntary. You may leave the study at any time. Leaving the study will not result in any penalty or loss of benefits to which you are entitled. If you have LF you will be referred to the local health center and be treated according to current treatment guidelines. The current treatment for LF is a single dose

of DEC and ALB given once a year for seven years. This is part of mass drug treatment (MDA) for LF administered by the PNG Board of Health.

WHAT ABOUT CONFIDENTIALITY?

We will keep the information we collect about you confidential. Your blood sample will not have any identifying information about you on it. Any test results we obtained as part of the study will be shared with the health care providers at the health center, only with your approval. This will occur even if you ineligible for the study or decide to withdraw at any time.

U.S. NATIONAL INSTITUTES OF HEALTH (NIH) CLINICAL TRIAL DATABASE: A description of this clinical trial will be available on http:///www.clinicaltrials.gov, as required by U.S. Law. This website will not include information that can identify you. At most, the website will include a summary of the results. You can search this website at any time to find out information about the trial and basic results.

WHAT ARE THE COSTS?

There is no cost to you to participate in this study. You will receive no payment for taking part in this study. All study drugs and laboratory tests will be paid for by the study. If you develop a local infection within 2-7 days at the site on your hand or arm from where we drew the blood, we will provide transportation to your local provincial health center and a voucher to cover the costs of medications to treat this infection.

STORAGE AND USE OF SAMPLES FOR FUTURE STUDIES

Samples of your blood will be stored in a freezer at the laboratories of the Papua New Guinea Institutes of Medical Research's laboratories and also at the laboratories of the Center for Global Health & Diseases at Case Western Reserve University in Cleveland, Ohio (USA), and may be used for future testing related to scientific studies not described here, including tests for genetic polymorphisms and immunity related to malaria, filariasis and other infectious diseases. However these samples will only be used with approval from the Papua New Guinea Institute of Medical Research's Institutional Review Board and the Principal Investigators' primary Institutional Review Board. You will not be contacted for additional consent. You may still participate in this study if you do not consent to us using your samples for future scientific studies about diseases affecting your community. If you check "no," then your samples will be stripped of your identification number in the database after the completion of this study and will not be used by the investigators after the study is completed.

If you change your mind in the future, you may contact Dr. Leanne Robinson or Dr. Moses Laman, Senior Research Fellows at Papua New Guinea Institute of Medical Research in writing or by phone at the Papua New Guinea Institute of Medical Research (Madang) (675/422-2909).

Consent for use of your blood samples for future studies (Please check one box only)	☐	yes	☐	no

Summary of your rights as a participant in a research study

Your participation in this research study is voluntary. Refusing to participate will not alter your usual health care or involve any penalty or loss of benefits to which you are otherwise entitled. If you decide to join the study, you may withdraw at any time and for any reason without penalty or loss of benefits. If information generated from this study is published or presented, your identity will not be revealed. In the event new information becomes available that may affect the risks or benefits associated with this study or your willingness to participate in it, you will be notified so that you can decide whether or not to continue participating in the study.

If you experience physical injury or illness as a result of participating in this research study, medical care is available at the local Health Center or, if more severe, you will be transported to the local district hospital. If you illness is determined to be related to taking the anti-filarial drugs, we will cover all appropriate medical costs.

Disclosure of your study records

Efforts will be made to keep the personal information in your research record private and confidential, but absolute confidentiality cannot be guaranteed. The University Hospitals Case Medical Center Institutional Review Board and/or the Papua New Guinea Institute of Medical Research Institutional Review Board may review your study records. In addition, for treatment studies, the study sponsor and possibly foreign regulatory agencies may also review your records. If your records are reviewed your identity could become known.

WHOM DO I CALL IF I HAVE QUESTIONS OR PROBLEMS?

____ has described to you what is going to be done, the risks, hazards, and benefits involved. The study coordinators, Livingstone Tavul or James Suamani can be contacted at 72930700 (Livingstone), 71066112 (James) (cell phones) if you have any questions. If you have any questions, concerns or complaints about the study in the future, you may also contact him later. Dr. Peter Siba, Director of the PNGIMR or his representative, can be contacted about individual rights as a research participant. If you are

unable to contact Mr. Suamani or Mr. Tavul, you may call the Institute of Medical Research (Madang) (675) 422-2909.

After we have finished distributing the drugs for lf, we will ask some of you how you felt about the study.

SIGNATURE

Signing below indicates that you have been informed about the research study in which you voluntarily agree to participate; that you have asked any questions about the study that you may have; and that the information given to you has permitted you to make a fully informed and free decision about your participation in the study. By signing this consent form, you do not waive any legal rights, and the investigator(s) or sponsor(s) are not relieved of any liability they may have. A copy of this consent form will be given to you.

Signature of Participant	Date
X	
Printed Name of Participant	

X	
Signature of Participant	Date
X	
Printed name of minor if used to obtain assent	
X	
Signature of Parent/Legal Guardian	Date

APPENDIX 6A: INFORMED CONSENT FORM [EXAMPLE]

X	
Printed name of Parent/Legal Guardian	
X	
If Legal Guardian, indicate relationship to child	

Study personnel (only individuals designated on the checklist may obtain consent)

X	
Signature of person obtaining informed consent	Date
X	
Printed name of person obtaining informed consent	
X	

Signature of Witness	Date
X	
Printed Name of Witness	

APPENDIX 6A: INFORMED CONSENT FORM [EXAMPLE]

Assessment of Informed Consent		
	Yes	No
Do you understand the consent form?		
Do you have any questions?		
Question:		
Do you have to participate in this study?		
Will you stay overnight at the Health Center during the study?		
Will we take blood from you during this study?		
Can you refuse to participate in the study at any time?		
Is there any charge for being in the study?		
Will you receive any money for being in the study?		
Do you know who to call if you have questions?		

Waiver of Consent and Assent Justification

Request for Waiver of Consent Documentation (45 CFR 46 117(c)(2) and 21 CFR 56.109(c)(1)) for Census, Georeferencing and Randomization portion of the study

The research presents no more than minimal risk or harm to the participants and involves no procedures for which written consent is normally required outside of the research context (45 CFR 46 117(c)(2) and 21 CFR 56.109(c)(1)).

The waiver of consent documentation is being requested for the census and geo-referencing portion of the study. The study procedures for this preliminary portion of the protocol will involve regional health workers and study team member collecting the name age and sex of each person who resides in the home. The information collected does not involve any physical risk to participants. If a head of household does not want to provide this information they can refuse. Consent is implied by the head of household and residents providing the study team members with this information. The information collected is standard information the regional health works have access.

Request for Waiver of Assent for minors ages 5-17 (45 CFR 46.408 and 21 CFR 50.55) for the Census, Georeferencing and Randomization portion of the study

Explain how the research involves no more than minimal risk.

APPENDIX 6A: INFORMED CONSENT FORM [EXAMPLE]

This portion of the study involves collecting of a minors name, age and sex. No physical procedures will be conducted that would involve risk to the child.

Explain why the waiver or alteration of assent will not adversely affect the rights and welfare of the participants.
It will be at the discretion of the head of household or the child's parents to determine if this information is provided. This is in line with the culture of Papua New Guinea and does not violate this child's rights.

Explain why the research could not practicably be carried out without the waiver or alteration of assent.
Without the census and geo-referencing portion of the study will assist the study team members in determine which communities in the region will be selected for this study. It is not feasible to collected written consent form all residents of a village since in the short amount of time needed to conduct the census and geo-referencing portion of this study. Potential participates will sign a consent form prior taking any study medication.

Request for Waiver of Assent for minors ages 7-13 (45 CFR 46.408 and 21 CFR 50.55) for the Evaluation for filariasis antigenemia and microfilaria & Two-drug therapy (DA) and triple drug therapy (IDA) portion of the study

This study is requesting a waiver of assent for minors ages 7-13 who are enrolled into the evaluation for filariasis antigenemia and microfilaria & Two-drug therapy (DA) and triple drug therapy (IDA) portion of the studyMinors ages 14-17 who participate in the valuation for filariasis antigenemia and microfilaria & Two-drug therapy (DA) and triple drug therapy (IDA) portion of the study will sign on the parental consent form

Explain how the research holds out a prospect of direct benefit that is important to the health or well-being of the children and is available only in the context of the research.

This process reflects the cultural norms and practices usually employed in studies in PNG, and respect the roles parents and the community have in the informed consent process. Minors from ages 5 to 7 years do not need to give assent to the study. Respecting the cultural norms and practices in PNG, minors between the ages of 7 and 13 will not sign an assent form. There will be direct benefit to minor's participating in this study, in that those infected will be treated for the LF infection and transmission to the whole community will be reduced under either regimen.

APPENDIX 7: TREATMENT ACCEPTABILITY STUDY PROTOCOL

Protocol for a treatment acceptability study following the Triple Drug Community Safety Trial

Finalized 4 May 2016

Research team

Alison Krentel PhD, Investigator, Bruyère Research Institute, Ottawa Canada
Joshua Bogus MPH, Global Health Project Manager for Operations, DOLF project, Washington University, USA
Research assistant, Bruyère Research Institute
Research coordinator to be determined in each country

A. Summary

As part of the larger "Community Based Safety Study of 2-drug versus 3-drug Therapy for Lymphatic Filariasis" a study to assess treatment acceptability in the community is planned in each research site: Papua New Guinea, Indonesia, Haiti, Sri Lanka and India. The overall aim of this research is to understand the community's acceptance of the 3-drug regimen as well as gain insight into the feasibility of administering this new therapy in the future. Part of the investigation will include assessing community member's perception of the possible adverse events experienced as a result of the 3-drug therapy, and how that might affect future rounds of mass drug administration (MDA) at the community level. Community acceptance will be measured using a survey to community members receiving treatment during the trial. In addition, focus group discussions (FGD) will be carried out with community members and community health workers to further investigate acceptability of the new therapy. To complement the community survey and focus group discussions, a series of key informant interviews are proposed with community leaders and health personnel in the same communities to assess perceptions about the 3-drug versus the 2-drug regimen as well as gain insight into the feasibility of distributing the new regimen as well as perceptions about managing adverse events.

B. Rationale for the study

With the introduction of a new treatment regimen for the elimination of lymphatic filariasis (LF), understanding community perceptions about the treatment, its adverse events (AE) as well as its efficacy will be an important component of assessing the acceptability of the 3-drug therapy. In particular, perceptions about the severity of experienced or observed AE, the efficacy of the treatment in killing the worms and understanding the positive presence of AE will be important to investigate.

Research has demonstrated the important impact that AE can have on individuals' acceptance of LF treatment using the 2-drug regimen [1, 2]. In some areas where MDA has been ongoing for many years, we might expect these AE to be objectively of minimal clinical significance, yet

subjectively community members continue to report “fear of AE” as a deterrent to comply with MDA. In recent research in a low prevalence area in Indonesia, 33% of individuals interviewed reported experiencing some form of side effect or AE as a result of taking the LF treatment (A. Krentel personal experience). Thomsen et al (2016) reported a higher rate of AE in those who were administered the 3-drug regimen versus those who received the 2-drug therapy [3]. As the wider application of this new therapy is considered, it will be important to understand if the perception of these AE is different in between the two treatment arms.

Another important deterrent to compliance with MDA is a lack of understanding of the benefit of treatment [4, 5]. The 3-drug regimen has been shown to be highly effective in the reduction of microfilariae [3]; therefore communicating this message to participants will be of crucial importance. Measuring participants’ understanding of this message will be essential in determining their acceptance of AE associated with the treatment. In PNG and in neighboring Indonesia when communities understand the reasons AE occur, they welcome them as a sign that the drugs are working [6, 7]. Knowing if this message also works with the 3-drug therapy where more AE are expected to occur is important in the future promotion of this treatment.

For the purposes of this research, a mixed method approach is recommended, combining the use of a community survey, focus group discussions and in depth interviews with key informants. The community survey will allow a robust comparison of treatment acceptability between those receiving the 2-drug regimen and those receiving the 3-drug regimen. A composite score will measure acceptability, combining outcomes like the respondents’ intention to take the treatment again and willingness to recommend it to other family members. Acceptability will be analyzed by the impact of some of the known factors that impact compliance: perception of AE, knowledge about AE, perceptions about the drug characteristics (safe, number of pills, taste), knowledge of vector, belief that the treatment is associated with health, and others. In order to assess the difference between the two treatment arms, the sampling frame for the community survey will take into account which regimen the individual received.

To complement the community surveys and provide further in depth analysis, focus group discussions (FGD) are planned with specific groups in the community, namely men, women, young people and community health workers. The FGDs will provide further insight and depth for some of the questions asked in the community survey. Specifically FGDs will investigate issues expected to relate to the 3-drug regimen: number of pills, perception of AE, how to ensure directly observed treatment and proposed messages to encourage compliance.

These results will be further substantiated by interviews with key community leaders, as well as community and professional health workers working in LF elimination at the village level. These interviews will provide an understanding of the macro level issues that key informants perceive as critical to consider with the use of the 3-drug therapy. With this, interview respondents will be

asked what advantages and concerns they have with regards to the 3-drug regimen based on their participation in and understanding of the safety trial.

The outcome of this research will provide operational recommendations to accompany the safety study. These will inform additional acceptability research if the 3-drug regimen is adopted as global policy. An important outcome will be to determine if there are any real differences in community acceptance of the 3-drug regimen when compared to the standard treatment. If there are any differences, then further investigation may be recommended. In addition, the global programme will need to consider how to adjust the delivery protocols and recommended messages used by community drug distributors giving out the 3-drug regimen. The acceptability study will provide a preliminary understanding of these issues and will provide important insights into the use of this regimen on a wider scale.

C. Study Objectives

A. Measure the perception of AE reported by safety trial participants, comparing those in the 2-drug versus 3-drug arms
B. Assess the overall acceptability in the community of the 3-drug regimen, as compared to the 2-drug regimen
C. Assess the overall acceptability in the community of those individuals who are MF positive, as compared to those who are MF negative
D. Investigate the acceptability and feasibility of delivering the 3-drug regimen

D. Community Survey

Community surveys are often called Knowledge, Attitudes and Practice (KAP) surveys because they use a cross sectional survey design to understand what community members know about disease, treatment and prevention; how they perceive factors related to the disease and finally what they do about it (e.g. take a drug, hang a bednet, use a condom). For the purposes of this survey, it is recommended to use a cross sectional survey design. However the terminology and format of the KAP may not be the most appropriate questionnaire design for the study proposed. Specific knowledge about LF disease is not a strong predictor for compliance in MDA for LF, with the exception of knowing that mosquitoes transmit LF [8, 9]. For the purposes of this research, focusing on knowledge of LF disease may not inform community acceptability of the 3-drug regimen as compared to the 2-drug regimen. Furthermore research has shown that there are important intrinsic reasons that affect people's decisions to take or not to take the LF treatment during MDA. Social norms of compliance, emotional cues, altruism and an individual's personal situation have all been shown to be associated with taking the LF drug [5, 10-12]. Understanding some of these intrinsic factors associated with taking the 3-drug regimen as opposed to the 2-drug regimen will be important in building a picture of community acceptability. As a result, although there may be similarities in some of the questions asked, it is

recommended to call the community survey a “treatment acceptability survey” as opposed to a “KAP survey.”

1. Timing
Coverage surveys are recommended to occur as soon as possible after MDA occurs in order to reduce recall bias in respondents [13]. In order to allow some space between the clinical assessment and monitoring of AE in the community trial as well as some time for the effects of ivermectin to become apparent, the community survey should occur at least two weeks after the completion of the drug administration, and preferably no later than one month afterwards.

Once the safety trial is completed, the community survey can begin.

2. Questionnaire Development
Questionnaire development is based on previous LF surveys carried out in Indonesia and in Papua New Guinea. In addition, known influences based on the most recent literature on compliance will be included in the acceptability survey, where appropriate.

Questionnaires will be written in English and translated into the local language. In order to test the understandability of the questionnaire with the local population, the enumerators will give advice on the vocabulary used during the training and a small sample of individuals will be administered the questionnaire prior to survey implementation. At the end of this testing, these respondents will be asked to comment on the questions themselves, whether they were clear and the language was appropriate. Changes will be made if needed. The questionnaire will then be translated back into English.

3. Sampling Frame
In estimating the sample size for the acceptability survey, one of the challenges we have is that we do not know the estimated acceptability rates in people who have received the 3-drug regimen. From recent research in Indonesia (A. Krentel, personal experience) in low (MF rate=1%) and high prevalence (MF=8%) areas, we know that acceptability with DEC+ALB, as measured in the intent to take the LF drugs again, was measured as 79% and 82% respectively.

Because we do not have a 3-drug acceptability rate, we cannot estimate the difference we might expect in between the regimen groups. As a result, this survey will create preliminary data, estimating the difference in acceptability rates between those individuals receiving the 2 and 3 drug regimens as well as the difference in rates between those with positive MF rates at the start of the safety trial and those who are MF negative. This survey will provide insight into possible trends in acceptability and will inform if further investigation is needed.

APPENDIX 7 TREATMENT ACCEPTABILITY STUDY PROTOCOL

In each country, one research site will be identified for the acceptability survey. 100 individuals will be interviewed in each of the four strata (2-drug, 3-drug, MF(-), MF(+)), totaling 400 individuals in each of the 5 countries:

- Strata 1: n=100 receiving the 2-drug regimen, MF (+) and antigen (+)
- Strata 2: n=100 receiving the 2-drug regiment, MF (-)
- Strata 3: n=100, receiving the 3-drug regimen, MF (+) and antigen (+)
- Strata 4: n=100, receiving the 3-drug regimen, MF (-)

In the low prevalence areas we recognize that it will not be possible to identify 200 MF (+) individuals, so in these locations we will oversample those who are MF positive until we have identified all of the individuals and the remaining sample will be filled with antigen positive individuals. For the purposes of analysis, we expect to combine the results from all five-research countries to increase the overall power in the sample.

In order to identify the participants in the sample, once the safety trials have been completed, the enrollment lists will be sent to the statistician at Washington University in St. Louis who will select individuals randomly according to the 4 stratums. Only one member of each household will be accepted for the survey. In addition, convenience factors will be taken into consideration, particularly where certain areas are remote and may be difficult for enumerators to reach in a timely manner.

Enumerators will travel to the house to interview the identified individual. Data will be collected using the REDCap system. Where individuals are not present at the time of the enumerator's visit, the enumerator can make a second attempt to reach them. After that, if they continue to remain unavailable, another randomly selected individual in the same treatment arm can replace them.

Because of the nature of the research questions, those individuals about the age of 14 years will be included in the survey sample. In addition, chronic manifestations of the disease begin to show at adolescence, so personal experience with LF may begin at this age [14].

4. <u>Outcome of Interest:</u>
Acceptability of the 3-drug therapy will be measured in a composite score from the following questions:

- Intention to take LF drugs in the future measured on a 5-point scale ranging from "I will never take this drug again" to "I will definitely take this drug again." (Adapted from Liau and Zimet 2001)

- Willingness to encourage other family members to take the LF drug, if offered in the future measured as a 5-point scale ranging from "I will never encourage my family to take the LF drugs" to "I will definitely encourage my family to take the LF drugs."
- Overall feeling about the LF elimination program as a 5-point scale ranging from "Very negative" to "Very positive"
- Perception of health since taking the LF drugs as a 5-point scale ranging from "Considerably worse" to "greatly improved"
- In addition to the scoring, each outcome can be converted to a binary variable for multivariate modeling.

- Inputs / Exposure variables:
- SES data
- Data from safety trial (clinical presence of AE, MF rate, household information)
- Treatment arm (2-drug versus 3-drug)
- Informed about the treatment before receiving the drug (e.g. did they receive any information)
- Belief in the efficacy of the treatment to eliminate / prevent LF (e.g. believe that the drugs work to prevent / treat LF)
- Belief in the efficacy of the treatment to treat scabies (e.g. believe that the drugs work to treat scabies)
- Belief in the efficacy of the treatment to treat other intestinal worms (e.g. believe that the drugs work to treat worms)
- Knowledge of the 'positive' component of AE (e.g. occur because the medicine is working)
- Perception of AE (e.g. none, mild, moderate, severe)
- Understanding that taking LF medicine is good for promoting health
- Knowledge that mosquitoes transmit LF
- Perception that the rest of the family/ household would take the LF drugs, if offered in the future (yes/no)
- Belief that the drug distributors are doing a good job (using a 10-point scale)
- Perceptions of the drugs (e.g. safe, neutral, dangerous)
- Components of the drugs (e.g. number, size, taste of pills)
- Emotions surrounding LF treatment (e.g. how does taking LF treatment make you feel?)

6. <u>Analysis</u>

For the data cleaning and data reduction, the following steps will be performed:

- Check response bias
- Clean the raw data set (range check, consistency checks)
- Transfer corrected data set to STATA statistical software (Stata Corporation, College Station, Texas).
- Group continuous variables into categorical variables, namely age. Recode certain variables where needed.

For the analysis, a descriptive analysis of the whole dataset will be prepared. The data from the community survey will be linked to the safety trial within the REDCap system.

Likert scales will be analyzed as both dichotomous and as continuous variables.

For both of the predictors of acceptability (drug regimen and presence of MF) logistic regression models will be created. Presence of AE as measured in the clinical surveys will be considered in the analysis, as will subjective perceptions of AE.

E. Focus Group Discussion

1. Timing

The focus group discussions will take place at the same time as the community survey, in the same communities.

2. Sampling Frame

For the focus group discussions, we will identify persons from specific groups of people: women of reproductive age, young people, men and community health workers. The rationale behind the selection of each of these groups is related to the prevailing evidence of their participation in MDA in the literature. Women of reproductive age often do not comply with treatment because they are either pregnant or breastfeeding, however they are often the gatekeepers for health in the household and ensure members of their household takes the treatment when offered. Men and young people have been known to be less compliant with MDA and so understanding their perceptions about the 3-drug regimen, MDA in general and soliciting their advice about how best to promote and reach their communities will be informative. Finally, as community health workers are usually the persons responsible for distributing the drug at the community level, understanding their perspectives on DOT, AE and messaging for the 3-drug regimen is important.

For the FGD, women, young men and men will be selected from the cohort of individuals receiving the 3-drug regimen.

3. Range of issues to explore include:
- How is LF elimination different / similar from the other health programs in their village?
- What are the health benefits from taking the treatment?
- What are the social benefits from taking the treatment?
- Do people like to take the pills in front of the distributor? Why or why not?
- How do you feel about the number of pills that you have to take?
- Why don't people want to take it?
- Did you have any side effects after you took the drugs (positive or negative)? How did you feel about them?
- What suggestions do you have to promote MDA to their community? Household?
- Are there any specific messages you would recommend to us?

4. Analysis
Recorded focus group discussions will be transcribed word for word in the local language. They will be translated into English. A second researcher with knowledge of English and the local language will check translation, sampling portions of each transcript and back translating them from English to the local language to check the reliability of the translation. The researchers will read through each transcript, recording emergent themes in an Excel matrix. NVivo will be used to assess trends and patterns in the interview transcripts.

F. In depth interviews with key informants

1. Timing
The key informant interviews will take place at the same time as the community survey, in the same communities.

2. Sampling Frame
A purposive sampling frame will be used, with individuals identified based on their leadership and cultural position with the village as well as their involvement with LF elimination and with the community trial. With this in mind, a range of 8-10 individuals will be included in the sample. In order to understand the acceptability of administering the 3-drug regimen, individuals to be interviewed would need to be those persons who are either directly involved with LF activities in the village or who would be involved in MDA in the future. Suggestions include community and/or religious leaders, community health workers, teachers.

3. Range of issues to explore include:
- What are the advantages of the 3-drug therapy in MDA? Disadvantages?

- What opportunities do they see in the administration of the 3-drug therapy, versus the 2-drug therapy?
- What concerns or challenges do they see in the administration of the 3-drug therapy, versus the 2-drug therapy?
- How do they feel about the number of pills that the community is asked to take?
- How do they feel about the side effects people might have / have?
- What suggestions do they have to promote MDA in this village? This province? The country? What messages would they recommend using?
- Which groups of people do they think will be difficult to reach with future MDA? Why? Any advice to approach them?

4. Analysis
Recorded interviews will be transcribed word for word in the local language. They will be translated into English. A second researcher with knowledge of English and the local language will check translation, sampling portions of each transcript and back translating them from English to the local language to check the reliability of the translation. The researcher will read through each transcript, recording emergent themes in an Excel matrix. NVivo will be used to assess trends and patterns in the interview transcripts.

G. Ethical Considerations

1. Community Survey
Ethical approval will be obtained from the local national research institution in each country as well as Washington University in St. Louis, Case Western University and Bruyère Research Institute.

Prior to giving consent to participate, the enumerator will read out the information sheet in the local language containing the aim of the survey, the length of time it is expected to take (15 minutes) as well as the protection of confidentiality for each respondent. Following this, each respondent will be asked to sign the informed consent form and where respondents are illiterate, a mark can be made. The enumerator will indicate that informed consent has been given. Age of eligible respondents is 14 years of age and older. For those aged 14 – 18 years, parental consent will be sought and provided on the informed consent form before the interview can begin. All forms will remain with the research team and will not contain any personal information other than the individual's signature.

At the end of the interview, each respondent will be given an information sheet with the principal investigator's contact details, should there be any questions. With this sheet, the respondent will

also receive a brief information sheet on lymphatic filariasis, the mass drug administration and who is eligible for treatment.

The data will be stored on Washington University servers during the duration of the study. After the study ends, electronic copies of the de-identified datasets will be kept by the PI indefinitely.

2. Focus Group discussions

Ethical approval will be obtained from the local national research institution in each country as well as Washington University in St. Louis, Case Western University and Bruyère Research Institute.

The interviewer will read the informed consent form to each person participating in the focus group discussion. The respondents will be asked to each sign an informed consent form for their participation. All interviews will be recorded with the permission of the respondent. Where permission is not granted, the interviewer will ask to take notes throughout the interview.

Any identifying information (name, address) will not be recorded. Individuals will not be identified in the transcripts or in the recordings and their anonymity will be maintained in all reporting and in the manuscripts. Transcripts of the interviews will remain with the research team.

The data will be stored with the PI, under password protection. After the study ends, electronic copies of the de-identified datasets will be kept by the PI indefinitely.

3. In-depth interviews with key informants

Ethical approval will be obtained from the local national research institution in each country as well as Washington University in St. Louis, Case Western University and Bruyère Research Institute.

The interviewer will read the informed consent form to each person participating in the interview. The respondents will be asked to sign an informed consent form for their participation. All interviews will be recorded with the permission of the respondent. Where permission is not granted, the interviewer will ask to take notes throughout the interview.

Any identifying information (name, address) will not be recorded and the identity of the respondent will be kept confidential in reporting. Transcripts of the interviews will remain with the research team. The data will be stored with the PI under password protection. After the study ends, electronic copies of the de-identified datasets will be kept by the PI indefinitely.

APPENDIX 7 TREATMENT ACCEPTABILITY STUDY PROTOCOL

References

1. Krentel A, Fischer PU, Weil GJ. A Review of Factors That Influence Individual Compliance with Mass Drug Administration for Elimination of Lymphatic Filariasis. PLoS neglected tropical diseases. 2013;7(11):e2447. Epub November 21, 2013. doi: doi:10.1371/journal.pntd.0002447.
2. Babu BV, Babu GR. Coverage of, and compliance with, mass drug administration under the programme to eliminate lymphatic filariasis in India: a systematic review. Transactions of the Royal Society of Tropical Medicine and Hygiene. 2014;108(9):538-49. Epub 2014/04/15. doi: 10.1093/trstmh/tru057. PubMed PMID: 24728444.
3. Thomsen E, Sanuku N, Baea M, Satofan S, Maki E, Lombore B, et al. Efficacy, Safety, and Pharmacokinetics of Coadministered Diethylcarbamazine, Albendazole, and Ivermectin for Treatment of Bancroftian Filariasis. Clinical Infectious Diseases. 2016;62(3):334-41. doi: 10.1093/cid/civ882.
4. Ramaiah KD, Kumar KNV, Hosein E, Krishnamoorthy P, Augustin DJ, Snehalatha KS, et al. A campaign of 'communication for behavioural impact' to improve mass drug administrations against lymphatic filariasis: structure, implementation and impact on people's knowledge and treatment coverage. Annals of Tropical Medicine and Parasitology. 2006;100(4):345-61. doi: 10.1179/136485906x105598. PubMed PMID: WOS:000238656500007.
5. Babu BV, Suchismita M. Mass drug administration under the programme to eliminate lymphatic filariasis in Orissa, India: a mixed-methods study to identify factors associated with compliance and non-compliance. Transactions of the Royal Society of Tropical Medicine and Hygiene. 2008;102(12):1207-13. doi: http://dx.doi.org/10.1016/j.trstmh.2008.05.023. PubMed PMID: 20083317171.
6. Bockarie MJ, Tisch DJ, Kastens W, Alexander NDE, Dimber Z, Bockarie F, et al. Mass treatment to eliminate filariasis in Papua New Guinea. New England Journal of Medicine. 2002;347(23):1841-8.
7. Krentel A, Fischer P, Manoempil P, Supali T, Servais G, Rückert P. Using knowledge, attitudes and practice [KAP] surveys on lymphatic filariasis to prepare a health promotion campaign for mass drug administration in Alor District, Indonesia. Tropical Medicine & International Health. 2006;11(11):1731-40.
8. Cantey PT, Rao G, Rout J, Fox LM. Predictors of compliance with a mass drug administration programme for lymphatic filariasis in Orissa State, India 2008. Tropical Medicine and International Health. 2010;15(2):224-31. doi: http://dx.doi.org/10.1111/j.1365-3156.2009.02443.x. PubMed PMID: 2010046751.
9. Mathieu E, Lammie PJ, Radday J, Beach MJ, Streit T, Wendt J, et al. Factors associated with participation in a campaign of mass treatment against lymphatic filariasis, in Leogane, Haiti. Annals of Tropical Medicine and Parasitology. 2004;98(7):703-14. doi: 10.1179/000349804x3135. PubMed PMID: WOS:000224651200006.
10. Krentel A, Aunger R. Causal chain mapping: a novel method to analyse treatment compliance decisions relating to lymphatic filariasis elimination in Alor, Indonesia. Health

Policy Plan. 2011. Epub 2011/06/30. doi: 10.1093/heapol/czr048. PubMed PMID: 21712348.

11. Fraser M, Taleo G, Taleo F, Yaviong J, Amos M, Babu M, et al. Evaluation of the program to eliminate lymphatic filariasis in Vanuatu following two years of mass drug administration implementation: results and methodologic approach. The American journal of tropical medicine and hygiene. 2005;73(4):753-8.

12. Nandha B, Sadanandane C, Jambulingam P, Das P. Delivery strategy of mass annual single dose DEC administration to eliminate lymphatic filariasis in the urban areas of Pondicherry, South India: 5 years of experience. Filaria J. 2007;6:7. Epub 2007/08/28. doi: 10.1186/1475-2883-6-7. PubMed PMID: 17718908; PubMed Central PMCID: PMC2020462.

13. Worrell C, Mathieu E. Drug Coverage Surveys for Neglected Tropical Diseases: 10 Years of Field Experience. American Journal of Tropical Medicine and Hygiene. 2012;87(2):216-22.

14. Witt C, Ottesen EA. Lymphatic filariasis: an infection of childhood. Tropical Medicine & International Health. 2001;6(8):582-606.

APPENDIX 7 TREATMENT ACCEPTABILITY STUDY PROTOCOL

Information Sheet And Informed Consent For Community Survey

As part of the "Community Based Safety Study of 2-drug versus 3-drug Therapy for Lymphatic Filariasis" that just happened in your area last month, we are asking some people who participated in that study to take part in a short survey so that we can understand more about lymphatic filariasis [or local name], the drugs used in the safety trial and health in general. Your name was selected randomly from the list of people who participated in that safety trial.

It is important that you understand why we are doing this survey, so please read this information sheet carefully. If you have any more questions, ask the interviewer and they will try to answer them for you.

We are interested in the experiences people had participating in the safety trial and what they understand about lymphatic filariasis [or local name]. We would like to talk to about 400 people in this area so that we can understand better how people felt about taking the LF drugs. Your participation is entirely voluntary and you are under no obligation to participate. Whether or not you choose to participate, your status and access to health care will not be affected in any way.

If you do choose to help with this study, we will only need about 15 minutes of your time to ask you some questions. At any time during this discussion, you are free to stop and withdraw from the study. You do not have to give the interviewer a reason.

The information that you provide during our discussion will be completely confidential. We will record your answers on a tablet. All digital files will remain with the main investigator and will be password protected.

APPENDIX 7 TREATMENT ACCEPTABILITY STUDY PROTOCOL

Consent for Community Survey

I have read the information sheet provided or it has been read to me concerning this study and I understand what will be required of me if I participate in this study, which will be a verbal interview and discussion.

My questions regarding this study have been answered by: _________________.

I understand that at any time I may withdraw from this study without giving a reason and without having any effect on my access to health care.

I agree to take part in this study.

Signature of the respondent: ______________________________________

Signature of a witness: ____

Signature of the enumerator to indicate that the informed consent has been read and the information sheet given to the respondent: __________________________________

APPENDIX 7 TREATMENT ACCEPTABILITY STUDY PROTOCOL

Information sheet and informed consent for in depth interviews with key informant

As part of the "Community Based Safety Study of 2-drug versus 3-drug Therapy for Lymphatic Filariasis" that just happened in your area last month, we are asking some people who participated in that study to take part in a verbal discussion so that we can understand more about lymphatic filariasis [or local name], the drugs used in the safety trial and health in general. It is important that you understand why we are doing this survey, so please read this information sheet carefully. If you have any more questions, ask the interviewer and they will try to answer them for you.

We are interested in the experiences people had participating in the safety trial and what they understand about lymphatic filariasis [or local name]. We would like to talk to about 8 people in this area so that we can understand better how people felt about taking the LF drugs. Your participation is entirely voluntary and you are under no obligation to participate. Whether or not you choose to participate, your status and access to health care will not be affected in any way.

If you do choose to help with this study, we will only need about one hour of your time to ask you some questions and to discuss informally. At any time during this discussion, you are free to stop and withdraw from the study. You do not have to give the interviewer a reason.

The information that you provide during our discussion will be completely confidential and we will not even write down your name or address. We will take some written notes during our discussion and if you agree, we may also record the interview using a digital recorder so that it will be easier to remember what we discussed. All digital files will remain with the main investigator and your name and address will not be recorded. We will write down the conversation and store it safely, with a password. Other researchers may ask to look at our discussion together, and we may share it with them, provided that they respect the same rules of confidentiality.

Consent for in depth interviews with key informant

I have read the information sheet provided or it has been read to me concerning this study and I understand what will be required of me if I participate in this study, which will be a verbal interview and discussion.

My questions regarding this study have been answered by: _________________.

I understand that at any time I may withdraw from this study without giving a reason and without having any effect on my access to health care.

I agree to take part in this study.

Signature of the respondent: ______________________________________

Signature of a witness: ____

Signature of the enumerator to indicate that the informed consent has been read and the information sheet given to the respondent: __________________________________

APPENDIX 7 TREATMENT ACCEPTABILITY STUDY PROTOCOL

Information Sheet And Informed Consent For Focus Group Discussion Participants

As part of the “Community Based Safety Study of 2-drug versus 3-drug Therapy for Lymphatic Filariasis” that just happened in your area last month, we are asking some people who participated in that study to take part in a focus group discussion so that we can understand more about lymphatic filariasis [or local name], the drugs used in the safety trial and health in general. It is important that you understand why we are doing this survey, so please read this information sheet carefully. If you have any more questions, ask the interviewer and they will try to answer them for you.

We are interested in the experiences people had participating in the safety trial and what they understand about lymphatic filariasis [or local name]. We would like to talk to about 4 groups of people in this area so that we can understand better how people felt about taking the LF drugs. Your participation is entirely voluntary and you are under no obligation to participate. Whether or not you choose to participate, your status and access to health care will not be affected in any way.

If you do choose to help with this study, we will only need about one hour of your time to ask you some questions and to discuss informally. At any time during this discussion, you are free to stop and withdraw from the study. You do not have to give the interviewer a reason.

The information that you provide during our discussion will be completely confidential and we will not even write down your name or address. We will take some written notes during our discussion and if you agree, we may also record the interview using a digital recorder so that it will be easier to remember what we discussed. All digital files will remain with the main investigator and your name and address will not be recorded. We will write down the conversation and store it safely, with a password. Other researchers may ask to look at our discussion together, and we may share it with them, provided that they respect the same rules of confidentiality.

Consent for Focus Group Discussion Participants

I have read the information sheet provided or it has been read to me concerning this study and I understand what will be required of me if I participate in this study, which will be a verbal interview and group discussion.

My questions regarding this study have been answered by: _________________.

I understand that at any time I may withdraw from this study without giving a reason and without having any effect on my access to health care.

I agree to take part in this study.

Signature of the respondent: ______________________________________

Signature of a witness: ____

Signature of the enumerator to indicate that the informed consent has been read and the information sheet given to the respondent: __________________________________

Effectiveness of 3-drug therapy for Lymphatic Filariasis elimination in Papua New Guinea: community prevalence and vector surveys to monitor LF indicators following mass drug administration

Protocol Identifier: DOLF_LF effectiveness surveys following IDA_PNG

Type: Cross-sectional community prevalence and vector surveys

DOLF Project Principal Investigator: Gary Weil, MD, Washington University, USA

Case Western University Principal Investigator: Christopher L King, MD, PhD,

Local Study Principal Investigators:
Moses Laman, MBBS PhD, PNG Institute of Medical Research, PNG
Leanne Robinson, PhD MPH, Burnet Institute, Melbourne; PNG Institute of Medical Research, PNG
Livingstone Tavul, PhD, PNG Institute of Medical Research, PNG
Stephan Karl, PhD, PNG Institute of Medical Research, PNG & WEHI, Melbourne

Study Co-Investigators:
Sibauk Bieb, MBBS, National Department of Health, PNG
Lucy Ninmongo John, MBBS MMED, National Department of Health, PNG
Leo Makita, National Department of Health, PNG
Mary Yohogu, National Department of Health, PNG
James Wangi, MPH, MBBS, MBBE, World Health Organisation, PNG
William Pomat, PhD, PNG Institute of Medical Research, PNG
Lincoln Timinao, MSc., PNG Institute of Medical Research, PNG
Tobias Maure, BSc., PNG Institute of Medical Research, PNG
Daniel Tisch, MPH PhD, Case Western Reserve University, Cleveland, USA
Alison Krentel, Alison Krentel PhD, Bruyère Research Institute, Ottawa Canada

Initial Protocol: 06 September 2017

Version: 1.3

Statement of Compliance

Effectiveness of 3-drug therapy for Lymphatic Filariasis elimination in Papua New Guinea: community prevalence and vector surveys to monitor LF indicators following mass drug administration

DOLF_LF effectiveness surveys following IDA_PNG v1.0 22 July 2017

This study will be carried out in accordance with Good Clinical Practice (GCP) as required by the:

- U.S. Code of Federal Regulations applicable to clinical studies (45 CFR 46)
  https://www.hhs.gov/ohrp/regulations-and-policy/regulations/45-cfr-46
- ICH GCP E6 Completion of Human Subjects Protection Training
  https://grants.nih.gov/grants/guide/notice-files/NOT-OD-16-148.html
- Bill and Melinda Gates Foundation "grant agreement" terms and conditions

SIGNATURES
I have read the protocol, including the appendices, and I agree that it contains all necessary details for me and my staff to conduct this study as described. I agree that this study will be conducted according to all stipulations of the protocol, including all statements regarding confidentiality and according to local legal and regulatory requirements and to the principles outlined in applicable U.S. federal regulations and ICH guidelines.
I will provide all study personnel participating in the study under my supervision copies of the protocol and access to all study related information provided by the DOLF project. I will discuss with them to ensure they are full informed about the study procedures.

Principle Investigator: _Gary Weil____________________

Name/Title (Print/Type)

Signed: ____________________ **Date:** 30 Oct 2017

Principle Investigator: _Moses Laman____________________

Name/Title (Print/Type)

Signed: ____________________ **Date:** 18/09/2017

Principle Investigator: __Leanne J. Robinson____

Name/Title (Print/Type)

Signed: ________________________________ **Date:** 18.09.17

Principle Investigator: _Christopher L. King____

Name/Title (Print/Type)

Signed: ________________________________ **Date:** 18.09.17

Principle Investigator: __Livingstone Tavul____

Name/Title (Print/Type)

Signed: ________________________________ **Date:** 18/09/2017

Principle Investigator: __Stephan Karl____

Name/Title (Print/Type)

Signed: ________________________________ **Date:** 18/09/2017

NOTE: Both the Project PI and Study PIs should have signed investigator agreements on file.

Table of Contents

Table of Contents

List of Abbreviations 6
1 PROTOCOL SUMMARY 7
2 BACKGROUND INFORMATION AND SCIENTIFIC RATIONALE 8
2.1 Country Specific Background 9
3 POTENTIAL RISKS AND BENEFITS 9
3.1 Risks of Blood Draw 9
3.2 Potential Participant and Community Benefit 9
3.3 Participant Participation and Cost 10
4 Study Objectives and Design 10
4.1 Study Objectives: Community effectiveness study. 10
4.1.1 Primary Objective 10
4.2 Study Design 10
4.3 Study Enrollment 11
4.4 Study Population 11
4.5 Social Mobilization 11
4.6 Community Census 11
4.7 Inclusion and Exclusion Criteria 11
4.8 Informed Consent 12
4.9 Screening for Circulating Filarial Antigenemia (CFA), Antibodies and Microfilaremia. 12
4.10 Assessment of Soil Transmitted Helminthe Infections 12
4.11 Withdrawal 13
4.12 Mass Drug Administration 13
4.13 Vector Collections 13
4.14 Community Acceptability of IDA and DA 13
5 Study Procedures 14
5.1 Blood tests for *Wuchereria bancrofti* infection 14
5.2 Stool Examination for Infection with STH 14
5.3 Mosquito identification and molecular diagnostics 14
5.4 Study Duration and Timeline 14
5.5 Location of sample processing and analyses 15
6 Instructions for Specimen Preparation, Handling, and Storage 15
6.1 Specimen Shipment 15
7 Safety Reporting and Safety Monitoring 15
8 STATISTICAL CONSIDERATIONS 16
9 Data Handling 16
9.1 Types of Data Collected 16
9.2 Study Records Retention 16
9.3 Participant Privacy 17
10 Future Use of Stored Specimens 17

11 INFORMED CONSENT PROCESS 17
11.1 Informed Consent/Assent Process (in Case of a Minor or Others Unable to Consent for Themselves. 17
12 Responsiblities 18
12.1 Good Clinical Practice 18
12.2 Institutional Review Board (IRB)/Ethics Committee (EC) 18
12.3 Data Ownership and storage 18
13 LITERATURE REFERENCES 18

List of Abbreviations

**ALB**	Albendazole
**CFA**	Circulating Filarial Antigenemia
**CFR**	Code of Federal Regulations
**DEC**	Diethylcarbamazine
**DA**	Diethylcarbamazine and Albendazole
**DOLF**	Death to Onchocerciasis and Lymphatic Filariasis
**DSMB**	Data Safety Monitoring Board
**EDTA**	Ethylenediaminetetra-acetic Acid
**FTS**	Filaria Test Strip
**GPELF**	Global Program to Eliminate Lymphatic Filariasis
**GCP**	Good Clinical Practice
**IATA**	International Air Transport Association
**ICH**	International Conference on Harmonisation
**IRB**	Institutional Review Board
**IDA**	Ivermectin, Diethylcarbamazine, and Albendazole
**KK**	Kato-Katz
**LF**	Lymphatic Filariasis
**MDA**	Mass Drug Administration
**MF**	Microfilaria(e)
**PI**	Principal Investigator
**RT**	Room Tempareature
**SOP**	Standard Operating Procedure
**STH**	Soil Transmitted Helminth
**TAS**	Transmission Assessment Survey
**WHO**	World Health Organization
**µl**	Microlitre

1 PROTOCOL SUMMARY

**Study Title:**	Effectiveness of 3-drug therapy for Lymphatic Filariasis elimination in Papua New Guinea: community prevalence and vector surveys to monitor LF indicators following mass drug administration.
**Type of Study:**	Cross-sectional Community Prevalence and Vector Surveys
**Population:**	Approximately 6000 people will participate per year. The study will include participants greater than or equal to 5 years of age who live in communities where safety studies of 2-drug versus 3-drug therapies for Lymphatic Filariasis were conducted. Participant selection will not be based on previous treatment, study participation, or health status.
**Number of Survey Areas:**	Bogia District
**Study Duration**	Approximately 12 months
**Duration of Participant Participation**	Participants will be studied in two cross-sectional surveys 12 and 24 months following initial treatment for lymphatic filariasis
**Primary Objective:**	To assess the effectiveness of mass drug administration with 2-drug (DA) or 3-drug (IDA) regimens on lymphatic filariasis infection parameters (circulating filarial antigenemia and microfilaremia) in communities.
**Secondary Objectives:**	1. To ***compare*** the impact of MDA with IDA or DA on the proportion of mosquitoes infected with *W. bancrofti* and the prevalence of anti-filarial antibody.<br>2. To ***compare*** the impact of MDA with IDA or DA on acquisition of anti-filarial antibodies in previously unexposed or serologically negative individuals.<br>3. To ***compare*** the impact of MDA with IDA or DA on soil-transmitted helminth (STH) infection parameters in communities and infection intensities (eggs per gram).<br>*4.* To ***assess*** whether community acceptability of IDA or DA changes over time, ascertain reasons for non-participation in the community based safety trial
**DOLF Project**	This protocol is specific to Papua New Guinea but results will also be considered together with data from study sites in other countries that have participated in the multi-center IDA safety study. Data will be analyzed at the country level and at the overall project level.

2 BACKGROUND INFORMATION AND SCIENTIFIC RATIONALE

Lymphatic filariasis (LF) is a parasitic worm infection caused by the filarial nematodes *Wuchereria bancrofti*, *Brugia malayi* and *Brugia timori*. Adult worms reside in the human lymphatic system and release immature forms (microfilariae) into the blood stream. These are taken up by mosquitoes that continue the cycle when they bite another person. Dying adult worms provoke disabling and disfiguring obstruction of the lymphatic vessels and can cause lymphedema leading to elephantiasis. In addition, the species *W. bancrofti* causes hydrocele in men and breast/ vulva enlargement in women. In 2000, WHO launched the Global Programme to Eliminate Lymphatic Filariasis (GPELF) to eliminate LF as a public health problem by 2020. Outside of Africa, WHO recommended the co-administration of diethylcarbamazine (DEC) and albendazole. The programme's current strategy to interrupt transmission relies on the annual single dose mass drug administration (MDA) of these 2 drugs (DA) given to the entire eligible population in endemic districts. The program has made significant progress driving microfilaria (MF) prevalence (a measure of the distribution of infection) below 1% in many areas of the world as of 2015 [1]. Despite this success in some endemic countries, MF prevalence has remained >1% in a many areas within a number of countries, including Papua New Guinea (PNG). Therefore, additional treatment strategies are needed to help interrupt transmission in these areas and ensure that the GPELF meets its goal by 2020 [2].

Results from a pilot efficacy study in PNG [3] showed that a single dose of three drugs (ivermectin, DEC, albendazole [IDA]) completely cleared *W. bancrofti* MF for at least 1 year in all participants, which was superior to the currently recommended two-drug regimen (DA). This observation was confirmed with results from larger individually randomized clinical trials in PNG and Cote d'Ivoire where 12 months after treatment IDA completely cleared MF in 98% and 87% of people, respectively, while DA cleared only 32% and 26% participants (King et al. unpublished data). Recently, large community-based safety studies that treated more than 23,000 participants across four countries were conducted to determine if IDA was safe and acceptable for use in MDA (DOLF Project, unpublished data). The WHO is reviewing results from these large safety studies to determine if IDA will be recommended for use in national MDA programs.

If IDA is approved by WHO and country programs, MDA programs are expected to take fewer rounds to reach elimination [4]. The current WHO guidelines for stopping MDA are based on treatment with 2-drug therapy and assume 5 annual rounds of treatment [5]. Following 5 rounds of MDA with sufficient coverage, national LF elimination programs are supposed to conduct transmission assessment surveys (TAS) to determine if MDA can stop. The guidelines currently recommend that smaller "pre-TAS" surveys use 1% MF prevalence as a pre-condition for performing the more comprehensive TAS. TAS results trigger the decision to stop providing MDA within an evaluation area. Currently, there is no information about what community indicators of infection look like following shorter IDA programs. It is possible that current WHO guidelines for stopping MDA may need to be modified for shorter MDA programs that use IDA. Observing the levels of infection indicators in a community following treatment with IDA will provide important information to the GPELF if IDA is approved for use in MDA programs.

There is an opportunity to survey the same communities in Bogia District that were treated with IDA as part of the DOLF Project's previous *Community Based Safety Study of 2-drug (Diethylcarbamazine and Albendazole) versus 3-drug (Ivermectin, Diethylcarbamazine and Albendazole) Therapy for Lymphatic Filariasis (IRB1601;MRAC16.07)*. Communities in this study were randomly assigned to receive IDA or DA treatment. A large percentage of individuals in these communities participated in

the study thereby approximating a mass distribution of the treatments. By surveying these communities 12 and 24 months following their initial treatment and repeating treatment in all individuals at the 12 month follow-up we will be able to assess the impact of MDA with IDA or DEC/Alb on lymphatic filariasis infection parameters at the level of communities.

2.1 Country Specific Background

PNG has some of the most heavily infected populations with lymphatic filariasis in the world and very few areas of PNG have received comprehensive treatment programs for lymphatic filariasis. One of the reasons for this is due to the cost and complexity of delivering five annual rounds of MDA with DA. The data from studies using IDA in PNG suggests that not only is IDA safe and efficacious but that 1-2 annual rounds of MDA with IDA might be sufficient to interrupt transmission. This would make delivering MDA for LF elimination far more achievable in PNG.

To determine whether one or two rounds of triple drug therapy (IDA) can better interrupt transmission compared to the standard treatment of (DA), we propose to undertake repeat community prevalence and vector surveys. As potential markers of interruption of LF transmission, we hypothesize that one or potentially two rounds of MDA with IDA can i) demonstrate reduction of community Mf rates to <1%, ii) show the lack of LF exposure/infection in previously uninfected individuals and iii) the failure of the uptake of LF parasites by mosquito vectors. By contrast we postulate that communities treated with one round DA in same area are unlikely to reach these endpoints. This community-based survey will be conducted in the Bogia District where we have already undertaken the safety, efficacy and acceptability of IDA versus DA in 2016-17 of approximately 4600 individuals. Treatment with IDA versus DA was randomized to different communities.

3 POTENTIAL RISKS AND BENEFITS

3.1 Risks of Blood Draw and Stool collections

Blood collection via finger prick is considered to be minimal risk and little or no discomfort is anticipated. The risk of infection is minimized by disinfecting the skin and the use of standard sterile technique. On occasion a participant may faint during or after the finger prick. Study personnel will be alert to participant reactions after the blood collection and will provide aid as needed. There may be some cultural sensitivity, negative social stigma or embarrassment associated with providing a small stool sample. The study team will therefore conduct very careful and thorough community awareness from the first survey to show what positive influences have risen from the study in relation to this component. The study team will also ensure procedures for the provision of stool samples are set up in a way that minimises this risk. .

3.2 Potential Participant and Community Benefit

The study will screen participants for parasitic infections and either directly treat or refer infected subjects for treatment at their local government health center. We will also provide data on infection rates to district health officials. This information will help the PNG Ministry of Health to improve programs to eliminate lymphatic filariasis.

Society will benefit from the information on the impact of IDA on filarial and STH infections at the community level.

3.3 Participant Participation and Cost

Participation is voluntary and participants may decline participation without consequences. There will be no cost to the participant to participate in the study and they will not be paid for their participation.

4 STUDY OBJECTIVES AND DESIGN

4.1 Study Objectives: Community effectiveness study.

4.1.1 Primary Objective

To assess the effectiveness of mass drug administration (MDA) with 2-drug (DA) or 3-drug (IDA) regimens on lymphatic filariasis infection parameters in communities. Parameters measured will include: circulating filarial antigenemia (CFA) assessed with the Filariasis Test Strip (FTS), and microfilaremia (assessed by night blood smears and microscopy). The primary endpoints for the effectiveness study will be rates of microfilaremia and CFA. The study will test the hypothesis that one or two annual rounds of MDA can reduce community infection parameters to targets established by WHO (microfilaremia <1% and CFA <2%) and interrupt transmission.

Secondary Objectives: Comparative MDA effectiveness study

1. To ***compare*** the impact of MDA with IDA or DA on the proportion of mosquitoes infected with *W. bancrofti*.
2. To ***compare*** the impact of MDA with IDA or DA on acquisition of anti-filarial antibodies and CFA in previously unexposed, uninfected or serologically negative individuals.
3. To ***compare*** the impact of MDA with IDA or DA on soil-transmitted helminth (STH) infection parameters in communities. Endpoints will be percent of persons with moderate or heavy infections, infection prevalence rates, and infection intensities (eggs per gram).
4. To ***assess*** whether community acceptability of IDA or DA changes over time, ascertain reasons for non-participation in the community based safety trial.

4.2 Study Design

The project is comprised of repeated annual cross-sectional surveys and vector collections in the same communities of Bogia District where community safety studies of 2-drug versus 3-drug therapies for Lymphatic Filariasis were conducted in 2016-2017. Communities that were assigned to 2-drug and 3-drug MDA as part of the safety study will be included in survey.

4.3 Study Enrollment

The study will survey communities approximately 12 and 24 months after they last received treatment. All villages that underwent MDA in 2016-17 in Bogia District will be included, with a total estimated population of 6000 – 7000.

The community prevalence surveys will be conducted at or about the same time as the 12-month follow-up visits for participants who are enrolled in the *Community Based Safety Study of 2-drug (Diethylcarbamazine and Albendazole) versus 3-drug (Ivermectin, Diethylcarbamazine and Albendazole) Therapy for Lymphatic Filariasis*. In that study participants who tested positive for lymphatic filariasis or who provided a baseline stool sample will be visited at 12 months to determine their infection status. Individuals who are enrolled in the community prevalence study who are re-visited as part of the safety study will not be re-tested. Their test results will be imported from the safety study dataset.

Proposed schedule:

- One year post-treatment: January – March 2018 (field studies), April – May 2018 data clean up and analysis.
- Two year post-treatment: January – March 2019 (field studies), April – May 2018 data clean up and analysis. June-July 2019 – write up results.

4.4 Study Population

Males and females will be included in population-based village prevalence surveys without regard to race, religion, or ethnic group. There is no reason to exclude pregnant women from the prevalence surveys but they will be excluded from MDA, as per PNG National Department of Health guidelines. There is also no reason to exclude children from the studies, and they are included. Exclusion of children less than 5 years of age from community prevalence surveys and MDA is justified because prevalence rates for filariasis tend to be very low in young children and IVM is not recommended for children younger than 5 years.

4.5 Social Mobilization

Prior to the survey, social mobilization activities will be conducted to ensure maximum community participation. This will include development and distribution of key messages to explain why it is important for the community to be tested in order to understand if they are still at risk of being infected.

4.6 Community Census

Recent census data from the study communities will be updated as part of social mobilization and awareness and the updated census will be used to identify and recruit participants into the study.

4.7 Inclusion and Exclusion Criteria

Inclusion Criteria

1. Age $\geq$ 5 years (males and females).
2. Able to provide informed consent (or parental/guardian consent for minors to participate in the study)

Exclusion Criteria

1. Unable or unwilling to provide informed consent or (for minors) lacking parental/guardian consent to participate in the study
2. Pregnancy (exclusion criteria for treatment but not prevalence survey)

4.8 Informed Consent

All participants will provide verbal informed consent before any study procedures are performed. Participation of minors (less than 18 years of age) will require their verbal assent and the verbal consent of at least one parent or legal guardian.

A waiver of written consent and a waiver for written documentation of verbal consent is justified, because this is a minimal risk study involving public health screening procedures that normally do not require documentation of consent outside of the research context. This is consistent with United States Title 45 CFR 46 Protection of human subjects, paragraph 46.117.c.2.

4.9 Screening for Circulating Filarial Antigenemia (CFA), Antibodies and Microfilaremia.

Blood samples will be collected from all eligible participants from the study communities. Blood will be tested for *Wuchereria bancrofti* antigenemia with the Alere Filariasis Test Strip (FTS) and dried blood spots will be collected to detect filarial antibodies. Blood from persons with positive filarial antigen tests will also be tested for microfilaremia.

In addition to the diagnostic tests, data collection will include demographic data, history of lymphedema, scrotal swelling (hydrocele), and prior treatment for LF.

4.10 Assessment of Soil Transmitted Helminthe Infections

Stool examinations will be performed for diagnosis of STH infections.

Expected number of positive individuals per treatment arm: Based on preliminary results, 78% of participants had hookworm infection. Results on strongyloides are pending (requires PCR analysis) and no other STH were detected.

Study sites: Bogia District

Collection of stool samples: We aim to collect stool samples from 300 participants in the IDA study villages and 300 participants in the DA study villages. We will follow the DOLF '*SOP for Stool Collection*'. A convenience sampling method will be used without special regard to sex and age, because drug efficacy is unlikely to depend on these parameters.

Detection and quantitation of STH in stool samples: We will examine stool samples of the study population mentioned above for the presence and the density of STH eggs. We will use the Kato-Katz (KK) methods to examine a single stool sample from each participant. These methods provide data on both the prevalence and intensity of infections with hookworm, *Ascaris*, and *Trichuris*. Molecular analysis will be used to detect strongyloides infection because parasitological techniques are insensitive. We will also use PCR analysis to confirm hookworm prevalence and intensity and other STH as required.

Participants will be informed that that the samples may also be tested with molecular methods for the presence of parasites and bacteria in stool. These samples may be archived for later testing beyond the period of this study. No HIV or human genetic testing will be performed.

4.11 Withdrawal

Participation in this project is completely voluntary, and participants may terminate their participation at any time. However, this is not very meaningful for cross-sectional studies, because a participants' role in the study is over after they have been tested. Since test results will be anonymous, once the test results are recorded they will be included in the analysis even if the participant withdraws.

4.12 Mass Drug Administration

Following the first survey the study teams will provide MDA for the study communities. Communities will be treated with IDA if WHO has approved IDA as an option for MDA by the time of study commencement. Treatment with IDA may be substituted with DA if there are not sufficient supplies of ivermectin in PNG.

Medical personnel from the study team or the local health clinic will be available to manage any adverse events related to treatment. Such adverse events are usually mild, transitory, and are related to the death of worms, and therefore occur most often in people with LF infection.

After the second survey the study will be completed and treatment will be the responsibility of the national LF elimination program.

4.13 Vector Collections

Xenomonitoring in the preceding trial indicated very high anopheline biting rates (average 120 bites per person night and an average 2.3% LF positivity in the mosquito population. Following the first round of MDA, this was reduced to 0.2%.

Xenomonitoring will be conducted immediately prior to the follow-up survey and MDA at the 12 month timepoint and immediately prior to the 24 month follow-up. This will be conducted in 4 or more villages (half with IDA, half with DA) that have baseline vector data and in 2 or more additional villages that did not have baseline xenomonitoring. Approximately 20 mosquito collectors will be enrolled from each village.

4.14 Community Acceptability of IDA and DA

A selection of acceptability questions based on key variables that were shown to be associated with acceptability in research following the community based safety trial will be included in the community prevalence survey questionnaire. These questions will be administered at the same time as the clinical assessment questions (either to all participants or, if deemed necessary due to operational considerations at the time of implementation, to a subset of participants).

5 STUDY PROCEDURES

5.1 Blood tests for *Wuchereria bancrofti* infection

All participants will be tested with the Alere Filariasis Test Strip (FTS) to detect filarial infection. The tests will be performed according to the manufacturer's instructions using capillary blood collected from finger prick. The antigen tests will be performed on blood from individual residents 5 years of age and older.

Participants with positive filarial antigen tests will be tested for microfilaremia with three-line thick smears prepared with a measured 60 µl quantity of finger prick blood collected between 8 pm and 2 am. Night blood samples are needed for microfilaria detection, because the parasites only circulate in peripheral blood at night. Slides will be fixed, stained with Giemsa, and examined by microscopy with a 10x objective according to the DOLF '*SOP for Giemsa Stain Preparation*' and '*SOP for Thick Blood Smears*'. The species of MF present will be determined by morphological criteria.

Serum eluted from dried blood spots will be tested with commercially available and validated tests for anti-filarial antibodies. Anti-filarial antibodies indicate current or past infection/exposure with filarial parasites and they can be used to assess the impact of MDA on filariasis endemicity and transmission in populations.

5.2 Stool Examination for Infection with STH

The primary diagnostic method will be the Kato-Katz test, which is widely used around the world for assessment of STH infection rates and intensities. Stool aliquots will be preserved for later examination by qPCR. This will enable us to archive STH DNA samples before and after IDA treatment to assess efficacy of MDA for filariasis on *Strongyloides* and other STH. We will follow the DOLF SOPs for *'Preparation of Kato-Katz Materials,' 'Stool Collection,' 'Stool Sample Management,'* and *'Kato Katz Procedure'.*

5.3 Mosquito identification and molecular diagnostics

Mosquitoes will be collected by using human landing catches and light traps. Individuals performing human landing catches will be offered malaria prophylaxis and to be tested for LF using the filarial test strip and for malaria using malaria RDT. If positive they will be offered treated with DA for lymphatic filariasis (or IDA if approved by WHO prior to drug administration) and for malaria according to PNG National Department of Health guidelines (currently artemisinin combination therapies). Malaria prophylaxis using low dose primiquine will be offered as an option to collector on days doing landing catches to prevent possible malaria infection. Collected mosquitos will be identified and those species known to transmit LF (primarily anopheline species) will then be examined for the presence of LF using PCR for *Wuchereria bancrofti* as previously described or with comparable assay methods.

5.4 Study Duration and Timeline

The consent process, collection of demographic data, and blood take less than 20 minutes to complete. Stool containers will be left with participants and will be collected no later than the following morning. Therefore, the duration of subject participation will be no more than one day. There is no follow-up planned.

One year later we will return to conduct a repeat cross-sectional survey. Many participants will be included in both surveys, but this is not a longitudinal cohort study. Verbal informed consent will be asked of all participants before each survey.

5.5 Location of sample processing and analyses

All efforts will be made to process, evaluate and analyze samples in laboratories at PNG IMR. In the event this is not possible for any number of reasons mutually agreed upon by project PIs, then the assays can be performed in laboratories at Case Western Reserve University, Washington University and/or Burnet Institute.

6 INSTRUCTIONS FOR SPECIMEN PREPARATION, HANDLING, AND STORAGE

Finger prick blood will be collected in microtainer tubes with EDTA or Heparin anticoagulant and CFA and MF tests will be performed within 48h following collection. FTS will be read in daylight or in a well-lit area 10 minutes after applying fresh finger prick blood to the test or blood collected in a microtainer. Persons with positive antigen tests will be tested for blood microfilaremia by thick smear examination (60 µl). Blood samples will be stored at RT or 4°C (if available) and not in the direct sunlight. Blood samples (no more than 40 µl) from finger prick will be placed on filter paper and air dried. Dried blood samples will be stored with dessicant at -20C (or at ambient temperatures during transport) in the dark.

Stool samples will be stored at RT and examined within 36 hours of collection for helminth eggs. Aliquots of stool can be dried on FTA cards for qPCR testing according to the procedure described in the DOLF SOP for '*Stool Sample Management*'.

Project personnel will treat all human blood and stool specimens as if they were infectious. Universal precautions for individuals collecting and working with blood samples to include proper disposal of contaminated materials (test strips, lancets, capillary tubes, blood film slides) will be in accordance with the guidelines prescribed by the local health authorities.

6.1 Specimen Shipment

Some preserved stool samples, blood smears and plasma extracted from DBS may be tested in parallel at collaborating laboratories at Case Western Reserve University (CWRU), Washington University in St. Louis and/or Burnet Institute for quality control. Shipments will comply with PNG rules and with IATA regulations.

7 SAFETY REPORTING AND SAFETY MONITORING

Study personnel will monitor subjects for adverse events related to blood collection. No medical monitor or DSMB is needed for this minimal risk, non-intervention study.

8 STATISTICAL CONSIDERATIONS

This study is observational in nature and is not hypothesis driven. However, we have chosen our sample size to be large enough to show that with high confidence that the true prevalence rate for MF is less than 1%. Current WHO recommendations for "pre-TAS" use this 1% MF prevalence as a pre-condition for performing TAS. TAS results trigger the decision to stop providing MDA within an evaluation area. Based on the assumption that the true MF rate is 0.5%, then a sample size of 1000 will yield 95% certainty that the measured MF rate will be less than 1%. Therefore our study sample of 6000 should be sufficient to show whether the true MF rate in the community meets the criteria for stopping MDA.

9 DATA HANDLING

Data will be collected using a smart phone based system. Phones will be pre-loaded with data collection forms. Field teams will be trained in the use of the instruments and data will be uploaded as entries are completed, provided there is internet access.

9.1 Types of Data Collected

Enrollment Data will include:

- Site Identification
- Participant Identifier
- Informed Consent Date
- Demographic Information
- Presence of hydrocele and lymphedema
- Bed Net and Window Screen Use
- History of prior MDA treatment

Laboratory Results

- FTS (filarial antigen test)
- FTS score
- MF slide (including MF count)
- LF Antibody results
- Soil Transmitted Helminthes egg counts and results of qPCR diagnostics.
- LF infected mosquitos using molecular diagnostic techniques (PCR).

9.2 Study Records Retention

Study documents will be retained for a minimum of three (3) years after the last participant has completed the study. These documents will be retained for a longer period, however, if required by local regulations. No record will be destroyed without the written consent of DOLF.

9.3 Participant Privacy

Privacy of the study participants will be maintained by assigning study participants a unique study identification number (UNID). All blood samples and laboratory results will be recorded and analyzed by UNID with no personal identifiers. All information collected, including demographic information about enrolled participants will be kept confidential and available only to the investigators and authorized study personnel such as the data manager.

No information concerning the study or the data will be released to any third party without prior written approval of the Principal Investigator. The study monitor or sponsor representatives may inspect all documents and records required to be maintained by the Investigator.

10 FUTURE USE OF STORED SPECIMENS

Residual specimens (serum, blood smears) may be maintained after the study is completed for future research on infectious diseases. Samples will be stripped of unique identifiers and stored in endemic country laboratories or in the laboratories Case Western Reserve University or at Washington University. Sequencing may be performed with DNA isolated from a small number of stool, blood samples or mosquito samples to detect and characterize worm parasite DNA (blood , stool, mosquito) and bacterial DNA (stool). The samples will be de-identified prior to any sequencing procedures. In addition, all human DNA sequences will be filtered out prior to bioinformatic analysis and no genetic testing of humans will be performed.

11 INFORMED CONSENT PROCESS

Informed consent is a process that is initiated prior to the individual's agreeing to participate in the study and continuing throughout the individual's study participation. Study physicians will read the consent script (with potential risks and benefits) in a locally understood language to participants and their families and leave a copy of this with each family. The consent document will be reviewed and approved by project IRB's prior to initiation of the study.

Only the principal investigators or study staff authorized and certified to obtain consent will consent participants for this study. Only individuals who have provided consent and meet eligibility criteria will be enrolled in the study.

Study staff will discuss risks and possible benefits of participation in this study with participants and their families. Study staff will explain the purpose of the study to participants and answer any questions that may arise. The participants may withdraw consent at any time throughout the course of the study. The rights and welfare of the participant will be protected by emphasizing to them that the quality of their medical care will not be adversely affected if they decline to participate in this study.

11.1 Informed Consent/Assent Process (in Case of a Minor or Others Unable to Consent for Themselves.

Inclusion of minor children less than 18 years of age in community surveys will require consent from at least one parent or guardian and assent of the child.

12 RESPONSIBLITIES

12.1 Good Clinical Practice

The investigator will ensure that the basic principles of Good Clinical Practice (GCP) are followed along with the appropriate laws and regulations of the country in which the research is conducted. Study personnel who will obtain consent from study participants will also receive training in the informed consent process and GCP.

12.2 Institutional Review Board (IRB)/Ethics Committee (EC)

The protocol and any accompanying material to be provided to the participants such as the informed consent will be submitted to the EC for review and approval. Approval from the committee must be obtained before starting the study and should be documented in correspondence to the investigator.

Any modifications to the protocol after receipt of the IRB or EC approval must be submitted to the committee for approval prior to implementation.

12.3 Data Ownership and storage

The data are the property of PNGIMR. The Principal Investigators, Co-investigators and key personnel may use the results of this study for publications, presentations at scientific meetings or as preliminary data for subsequent grant applications. Confidentiality of study participants will be maintained by not using names or personal identifiers. PNGIMR will provide de-identified data from the study to DOLF for use in publications and presentations that present results across different study sites. At least one PNGIMR researcher will be included as an author for any publications with data from PNG.

The study site Project Coordinator will permit access to all documents and records that may require inspection by the funding agencies, governmental regulatory agencies, institutional review boards or its authorized representatives. The PIs of the study will have full access to all the data during the course of the study. Data will be stored on servers at PNG IMR, CWRU and/or Washington University.

13 LITERATURE REFERENCES

1. Hooper, P.J., et al., *Assessing progress in reducing the at-risk population after 13 years of the global programme to eliminate lymphatic filariasis.* PLoS Negl Trop Dis, 2014. **8**(11): p. e3333.
2. Ichimori, K., et al., *Global programme to eliminate lymphatic filariasis: the processes underlying programme success.* PLoS Negl Trop Dis, 2014. **8**(12): p. e3328.
3. Thomsen, E.K., et al., *Efficacy, Safety, and Pharmacokinetics of Coadministered Diethylcarbamazine, Albendazole, and Ivermectin for Treatment of Bancroftian Filariasis.* Clin Infect Dis, 2016. **62**(3): p. 334-41.
4. Irvine, M.A., et al., *Effectiveness of a triple-drug regimen for global elimination of lymphatic filariasis: a modelling study.* Lancet Infect Dis, 2016. **17**(4): p.451-458.
5. WHO/Department of Neglected Tropical Diseases. Lymphatic filariasis: monitoring and epidemiological assessment of mass drug administration. A manual for national elimination programmes. WHO/HTM/NTD/PCT/2011.4.
   See http://www.who.int/lymphatic_filariasis/resources/9789241501484/en/

6. Bockarie MJ, Fischer P, Williams SA, Zimmerman PA, Griffin L, Alpers MP, et al. Application of a polymerase chain reaction-ELISA to detect Wuchereria bancrofti in pools of wild-caught Anopheles punctulatus in a filariasis control area in Papua New Guinea. The American journal of tropical medicine and hygiene. 2000;62(3):363-7.

APPENDIX A: Informed Consent for Community Surveys

A1 Information Sheet for participants of community surveys

Project title: **Community Prevalence Surveys to Monitor Lymphatic Filariasis Indicators Following Treatment with 3-drug Therapy in Papua New Guinea**

Study PIs: **Dr Moses Laman, Dr Leanne Robinson, Dr Christopher L. King, Dr Livingstone Tavul, Dr Stephan Karl**

Introductions: You and your family members are being invited to participate in a project to test the impact of a public health program on parasite infections that are common in this area.

Purpose: The purpose of this project is to understand how treatment for filariasis and other worm infections has worked in your community. This project is being done by PNGIMR together with scientists at Burnet Institute, Australia, Washington University in St Louis, USA and Case Western Reserve University in Cleveland, USA. About 6000 people will participate in the project.

Procedures: You will be in this study for one year. Today we will collect a small amount of blood from your finger by fingerpick to see if you are infected with LF and to test for antibodies. We will return in approximately one year and collected another fingerpick blood sample. Before we collected any blood, we will clean your finger with alcohol, and a few drops of blood will be collected. We will keep some blood to do other research studies of infectious diseases in the future. We will also ask you for information such as name, age, and address or house location. It will take a few minutes of your time for you to answer our questions and to collect the blood samples.

We will ask if you are willing to provide a small amount of your stool the approximate size of a betel nut today and will return in approximately one year to collect another stool sample. The stool sample will be used to test for other worms that live in your gut and to see how well the drug treatment programs worked on reducing the burden of these worms in your community.

All blood and stool samples will be kept in PNGIMR laboratories in Madang and Goroka. Some samples may be sent to other laboratories to check that the tests for worms were done correctly.

After these samples have been collected, you will receive standard treatment for LF and other worm infections recommended by the PNG National Department of Health.

Risks: The risk of drawing blood from a finger prick is minimal. This may cause a little pain and sometimes a small bruise. You will be asked to keep pressure with cotton on your finger for 2 minutes to stop bleeding.

Benefits: Participating in this health project will benefit you and your family by providing testing for worm infections. If you or any of your family members have worm infections, you will be referred to your local health center for treatment. The project will also help the Ministry of Health understand how much disease is in your community.

Costs: You will not be charged to be in the study. You will not be given any gifts to be in the study.

Confidentiality: We will keep your identity secret. Your name and information about you will be kept in a safe place and can only be seen by the study team. A code using only numbers will be used to identify your blood and stool samples. Your information will be kept secret even if you stop the study.

Alternatives to Participation Your participation in this public health project is voluntary. Children should not be forced to participate if they do not want to. You will not be punished if you do not want to volunteer. Your worms can still be treated during any regular government run community treatment program. You may stop the study at any time and you do not have to give a reason.

U.S. NATIONAL INSTITUTES OF HEALTH (NIH) CLINICAL TRIAL DATABASE: A description of this clinical trial will be available on http://www.clinicaltrials.gov, as required by U.S. Law. This website will not include information that can identify you. At most, the website will include a summary of the results. You can search this website at any time to find out information about the trial and basic results.

STORAGE AND USE OF SAMPLES FOR FUTURE STUDIES:
Samples of your blood will be stored in a freezer at the laboratories of the Papua New Guinea Institutes of Medical Research's laboratories and also at the laboratories of the Center for Global Health & Diseases at Case Western Reserve University in Cleveland, Ohio (USA) and/or Burnet Institute, Melbourne, Australia and may be used for future testing related to scientific studies not described here including tests related to infection related to malaria, filariasis and other infectious diseases. We will not use your samples for human genetic studies. However these samples will only be used with approval from the Papua New Guinea Institute of Medical Research's Institutional Review Board and the Principal Investigators' primary Institutional Review Board. You will not be contacted for additional consent. You may still participate in this study if you do not consent to us using your samples for future scientific studies about diseases affecting your community. If you check "no," then your samples will be stripped of your identification number in the database after the completion of this study and will not be used by the investigators after the study is completed. If you change your mind in the future, you may contact Dr. Moses Laman, Head of Vector Borne Diseases Unit at Papua New Guinea Institute of Medical Research in writing or by phone at the Papua New Guinea Institute of Medical Research (Madang) (675/422-2909).

Consent for use of your blood samples for future studies yes no (Please tick one box only)

YES ☐

NO ☐

Summary of your rights as a participant in a research study

Your participation in this research study is voluntary. Refusing to participate will not alter your usual health care or involve any penalty or loss of benefits to which you are otherwise entitled. If you decide to join the study, you may withdraw at any time and for any reason without penalty or loss of benefits. If information generated from this study is published or presented, your identity will not be revealed. In the event new information becomes available that may affect the risks or benefits associated with

this study or your willingness to participate in it, you will be notified so that you can decide whether or not to continue participating.

If you experience physical injury or illness as a result of participating in this research study, medical care is avalible at the local Health Center of if more sever, you will be transported to the local district hospital.

Disclosure of your study records

Efforts will be made to keep the personal information in your research record private and confidential, but absolute confidentiality cannot be guaranteed. The University Hospitals Cleveland Medical Center Institutional Review Board and/or the Papua New Guinea Institute of Medical Research Institutional Review Board may review your study records. If your records are reviewed your identity could become known.

CONTACT INFORMATION

____ has described to you what is going to be done, the risks, hazards, and benefits involved, and can be contacted at _______________________. If you have any questions you can call or contact the study coordinator. Dr William Pomat, PNG IMR Acting Director, or his representative can be contacted about individual rights as a research subject. If you are able to get to a telephone, you may call the Institute of Medical Research (422-2909) in Madang. There are no toll free telephone numbers in Papua New Guinea.

A2 Signature Sheet for participants of community surveys

Project title: **Community Prevalence Surveys to Monitor Lymphatic Filariasis Indicators Following Treatment with 3-drug Therapy in Papua New Guinea**

Study PIs: **Dr Moses Laman, Dr Leanne Robinson, Dr Christopher L. King, Dr Livingstone Tavul, Dr Stephan Karl**

SIGNATURE FORM

Signing below indicates that you have been informed about this study in which you voluntarily agree for your participation; that you have received the study information brochure; that you have asked any questions about the study that you may have; and that the information given to you has permitted you to make a fully informed and free decision about your willingness to collect mosquitos for this study. By signing this consent form, you do not lose any rights, and the investigators are not relieved of any obligations they may have. A copy of this consent form will be provided to you. If you do not know how to write, then an independent witness to the consent process will be asked to also sign on your behalf.

X	
Signature of Participant	Date
X	
Printed Name of Participant	

X	
Signature of Participant	Date
X	
Printed name of minor if used to obtain assent	
X	
Signature of Parent/Legal Guardian	Date
X	
Printed name of Parent/Legal Guardian	
X	
If Legal Guardian, indicate relationship to child	

X	
Signature of Witness	Date

X	
Printed Name of Witness	

Study personnel (only individuals designated on the checklist may obtain consent)

X		
Signature of person obtaining informed consent		Date
X		
Printed name of person obtaining informed consent		

Note: Program personnel must mark each electronic record to verify that they have read this script to all participants and answered any questions. A copy of this information form should be left in each house included in the program.

A3 Toksave pepa

Project title: **Community Prevalence Surveys to Monitor Lymphatic Filariasis Indicators Following Treatment with 3-drug Therapy in Papua New Guinea**

Study PIs: **Dr Moses Laman, Dr Leanne Robinson, Dr Christopher L. King, Dr Livingstone Tavul, Dr Stephan Karl**

Mipela invitim yu na femili bilong yu long stap insait long dispela wok painim aut long testim wok bilong dispela pablik helt program bilong dispela binantang we i save kamap planti long dispela peles.

AS TINGTING BILONG DISPELA WOK PAINIM AUT

Dispela wok painim aut i kamap long save sapos dispela marasin bilong filariasis na arapela sik bilong liklik sinek i stap insait long peles bilong yu. Dispela em wanpela wok painim aut we PNGIMR wantaim ol scientist bilong Burnet Institue, Australia na Washington University long St Louis, USA na Case Western Reserve University long Cleveland, USA. Em bai gat 6000 manmeri bai stap insait long dispela wok painim aut.

WEI BILONG MEKIM WOK

Yu bai stap insait long dispela wok painim aut long wanpela yia. Tete bai mipela kisim liklik blut long pinga bilong yu long sekim sapos yu gat binatang bilong sik bik lek na lo testim sapos blut bilong yu i soim olsem yu bin gat dispela binatang insait long bulut bipo, ol i kolim antibodies. Bai mipela bai kam bek behain long wanpela yia i go pinis na kisim liklik blut gen long pinga bilong yu. Mipela bai klinim skin long pinga bilong yu wantaim sampla marasin bilong klinim skin na behain bai mipla kisim liklik blut. Mipela bai holim sampla blut bilong behain taim long painim aut sampla arapela sik. Mipela bai askim yu sampla infomesen olsem nem, krismas, na we ples na haus bilong yu stap long em. Em bai mipla kisim liklik taim bilong yu tasol long askim ol askim pas bipo mipla kisim liklik blut bilong yu.

Mipla bai askim sapos yu ken givim liklik hap pekpek bilong yu long size bilong buai na mipla bai kam bek taim wanpla yia i go pinis long kisim liklik hap pekpek gen. Mipla kisim pekpek long sekim sapos ol liklik sinek i stap insait long bel bilong yu na tu long lukim sapos marasin mipela bin givim i wok gut long rausik ol dispela liklik sinek long ol manmeri long ples bilong yu.

Olgeta blut na pekpek bai stap insait long PNGIMR laboratory boxice long Madang na Goroka. Sampela bai mipla salim go long ol arapla laboratories lo sekim sapos tes bilong liklikl sinek mipla mekim em i kamap stret.

Taim olgeta samting mipla kisim pinis, yu bai kisim marasin bilong sik bik lek na narapela marasin bilong sik bilong liklik sinek we PNG national helt department i markim long em.

WONEM KAIN HEVI BAI STAP INSAIT LONG DISPELA WOK PAINIM AUT?

Hevi bilong pulim blut bilong yu long pinga bai liklik tasol. Em bai pen liklik na sua ken kamap. Bai mipla askim yu long holim go daun kapuk long pinga bilong yu inap long tupela minit long stopim blut long pinga.

IGAT SAMPELA GUTPELA SAMTING BLONG MI SAPOS MI WOK BUNG INSAIT LONG DISPELA WOK PAINIM AUT?

Stap insait long dispela wok painim aut bai helivim yu na femili bilong yu long sekim sik bilong ol liklik sinek. Sapos yu o wanpela long femili bilong yu gat sik bilong liklik sinek, bai mipla askim yu long go long hausik bilong yu long kisim marasin bilong liklik sinek. Dispela wok painim aut bai helpim Ministry bilong Health save gut long hamaspla sik i stap insait long ples bilong yu.

BAIM BILONG STAP INSAIT LONG WOK PAINIM AUT

Yu nonap baim moni long stap insait long dispela wok painim aut. Bai mipla nonap givim yu ol kainkain gutpla samting nambaut long stap insait long dispela wok painim aut.

MI LAIK SAVE WANEM SAMTING BAI YUPELA MEKIM LONG HAITIM OL INFOMASEN BILONG MI?

Mipela bai lukautim gut tru ol infomasen o stori bilong yu. Nogat narapela manmeri bai save long stori bilong yu. Wanpla mak long namba tasol bai stap long blut na hap pekpek bilong yu, na mipla nonap putim nem bilong yi, em bai hait. Sapos yu lusim dispela wok painim aut, mipla bai haitim ol stori bilong yu wea mipela bin kisim long yu taim yu bin stap yet insait long dispela wok painim aut.

NARAPELA TINGITNG LONG LUKSAVE

Yu stap insait long dispela wok painim aut em long laik bilong yu yet. Noken tok strong long ol pikinini long stap insait long dispela wok painim aut sapos ol i les long stap insait. Bai nogat nogut samting bai kamap long yu sapos ol pikinini les long stap insait long dispela wok painim aut. Yu yet ken stop long stap insait long dispela wok painim aut sapos yu laik na yu no nid long tokim mipla wanem as yu laik lusim.

U.S. NATIONAL INSTITUTES OF HEALTH (NIH) CLINICAL TRIAL DATABASE: Dispela wok bai stap long intanet long http://www.clinicaltrials.gov, we bihainim U.S law. Dispela bai ino nap soim infomasen o nem bilong yu. Dispela bai givim liklik infomasen long wanem samting ol painim long em. Na tu yu ken go long intanet na sekim ol infomasen long dispela wok na sampela samting wea ol painim long em.

ROT BLONG LUKAUTIM BLUT BILONG YU NA USIM LONG OL WOK PAINIM AUT LONG BEHAIN TAIM.

Taim mipela mekim pinis ol wok painim aut wantaim blut bilong yu, mipela bai putim blut insait long strongpela bokis ais (freezer) we istap long laboratory bilong PNG Institute of Medical Research na tu long Center for Global Health & Diseases long Case Western Reserve University long America. Mipela bai lukautim stap long wanem nogut bai mipela usim ken dispela blut long mekim sampela moa wok painim aut long bihain taim, kain olsem sampela wankain wok painim aut long malaria, sik bik lek na ol narapela sik. Mipela nonap yusim blut bilong yu bilong kainkain tes bilong blut na skin. Tasol mipela no inap usim blut bilong yu long ol narapela wok painim aut long bihain taim inap mipela kisim tok orait long Institutional Review Board blong PNG Institute of Medical Research na tu long ol Prinicipal Investigators' primary Institutional study. Mipela no inap long toksave long yu na askim tok orait blong yu ken sapos mipela laik usim dispela blut bilong yu long bihain taim. Yu ken still stap yet insait long dispela wok painim aut sapos yu no laikim mipla long yusim blut bilong yu bilong wok painim aut bihain taim. Sapos yu tok 'nogat' bai mipela rausim namba bilong yu wea i makim blut long pepa bilong mipela na mipela nonap yusim blut bilong yu long bihain taim. Sapos yu senisim tingting bilong yu behain taim, yu ken contactim Dr Moses Laman, senior wokman bilong wok painaim aut long PNG Institute of Medical Research o kolim em long telefon namba (Madang) (675/422-2909).

Yu wanbel long mipela lukautim na usim blut blong yu long ol narapela wok painim aut long bihain taim?<br>(Makim wanpela bokis tasol)	☐	Yes	☐	Nogat

RAITS BLONG YU SAPOS EM STAP INSAIT LONG DISPELA WOK PAINIM AUT

Em laik blong yu long oraitim yu yet o pikinini blong yu long stap insait long dispela wok painim aut. Sapos yu ino wanbel, dispela bai no inap pasim yu long kisim halivim long haus sik na ol narapela halivim we yu gat rait long kisim. Na tu sapos mipela putim long ripot o presentim ol infomasen mipela kisim long dispela wok painim aut, mipela bai no inap kolim nem o tokaut ol hait stori blong yu. Mipela bai toksave long yu sapos sampela senis kamap bihain we inap givim hevi o halivim long yu o mekim yu tingting long lusim wok painim aut.

Sapos yu sik o bungim hevi long bodi bilong yu taim yu stap insait long dispela wok painim aut, igat ol halivim i stap long locol Helt senta o, sapos bikpela hevi tumas, kar bai kisim yu long locol district hausik. Sapos sik i kamap long yu taim yu kisim marasin mipela i bin givim yu, bai mipela baim hausik bilong yu.

PASIN BLONG HAITIM O SOIM OL INFOMASEN BLONG YU

Nogat narapela manmeri bai save long wanem samting mipela painim aut long yu long taim mipela mekim dispela wok painim aut wantaim em. Mipela bai haitim ol hait infomasen o stori blong yu long ol narapela manmeri. Tasol yu mas save olsem ino isi long haitim stori olsem na igat sans olsem stori bilong yu ken go aut. Long sait blong nem bilong yu tasol em nogat manmeri bai save. Univeristy Hospital Cleveland Medical Center nstitutional Review Board na PNG IMR Institutional Review Board bai lukim stori bilong yu. Yu mas save olsem sapos planti lain lukim stori blong yu, igat sans olsem ol ken lukim nem blong yu wantaim stori bilong yu tasol dispela ino save kamap tumas.

HUSAIT BAI YU LUKIM SAPOS YU GAT ASKIM OR HEVI?

____ itok klia pinis long wanem ol hevi na ol gutpela samting bai kamap long dispela wok painim aut. Yu inap kolim tu hetman blong dispela wok painim aut, wok painim aut cooordinator. Dr William Pomat, Actin Dairekta bilong PNGIMR o ol het wokman bilong em long save gut long raits bilong yu long stap insait long displa wok painim aut. Sapos yu inap long kism telepon, yu ken ringim Institute of Medical Research (422-2909) long Madang. Em i nogat telepon namba wea ol nonap sasim yu long ringim ol insait long Papua New Guinea.

A4 Hanmak pepa

Project title: **Community Prevalence Surveys to Monitor Lymphatic Filariasis Indicators Following Treatment with 3-drug Therapy in Papua New Guinea**

Study PIs: **Dr Moses Laman, Dr Leanne Robinson, Dr Christopher L. King, Dr Livingstone Tavul, Dr Stephan Karl**

HAN MAK BLONG YU

Han mak bilong yu i soim olsem yu bin askim pinis olgeta askim wea yu gat na yu klia long as tingting blong dispela wok painim aut. Olsem na yu wanbel long stap insait long dispela wok painim aut. Dispela han mak blong yu ino pasim yu long ol raits bilong yu. Dispela tu ino soim olsem mipela ken abrusim ol nogut we mipla mekim long yu.

Han mak bilong man/meri husait bai stap insait long wok painim aut Dei (Date)	
X	
Nem bilong man/meri husait bai stap insait long wok painim aut	

X	
Han mak bilong man/meri husait bai stap insait long wok painim aut Dei (Date)	
X	
Nem bilong pikinini sapos mama o papa tok orait long stap long stadi	
X	

Han mak bilong papa/mama o wasman husait bai tok orait long piknini long stap insait long stadi
X
Nem bilong papa/mama o manmeri husait bai tok orait long piknini long stap insait long stadi
X
Sapos wasman bilong pikinini, hau em save long dispela pikinini

Study personnel (only individuals designated on the checklist may obtain consent)

X
Han mak bilong wok man/meri ………………………………….. Dei (date)
X
Nem bilong wok man/meri

Han mak bilong man/meri husait stap na lukim Dei (date)
Nem bilong man/meri husait stap na lukim

Luksave bilong dispela pepa		
	Yes	Nogat
Yu kisim klia tingting long ol askim insait long dispela pepa o nogat?		
Yu gat sampela askim o nogat?		
Askim:		
Bai yu stap insait long dispela wok painim aut o nogat?		
Bai yu stap long Helt Center long nait long taim blong wok painim aut?		
Bai mipela kisim blut blong yu long dispela wok painim aut?		
Inap yu les long wok bung wantaim mipela long dispela wok?		
Bai igat sampela sas sapos mi stap insait long dispela wok painim aut?		
Bai yu kisim sampela moni sapos yu stap insait long dispela wok painim aut?		
Yu save long wanem man long kolim taim yu gat sampela askim?		

APPENDIX B: Informed Consent for Mosquito Collectors

B1 Information Sheet for Mosquito Collectors

Project title: **Community Prevalence Surveys to Monitor Lymphatic Filariasis Indicators Following Treatment with 3-drug Therapy in Papua New Guinea**

Study PIs: **Dr Moses Laman, Dr Leanne Robinson, Dr Christopher L. King, Dr Livingstone Tavul, Dr Stephan Karl**

This is a research study. Research studies include only people who choose to take part. Please take your time to make your decision. Discuss it with your friends and family.

The purpose of this study is to find out the relationship between the number of mosquitoes in your community and the diseases that they transmit in your village. The investigators hope to learn more about what kind of mosquitoes are found in your village and homes on the Lihir Islands.

You are invited to work as a mosquito collector because you are over 18 years old, live in this community, and have received training in how to collect mosquitoes for a research study.

As a mosquito collector, you can be exposed to mosquitoes that can transmit malaria and other sicknesses. The risk of being bitten by mosquitoes that transmit malaria or other sicknesses is the same or less as other community residents who do not act as mosquito collectors. However, you will be offered a preventive malaria treatment (chemoprophylaxis) for the duration of the mosquito collections and the following 7-14 days to avoid any malaria infection due to your activity as a collector.

The risk of receiving mosquito bites may actually be less than you would otherwise experience, as you will remain awake and will be taught how to catch the mosquitoes that land on the skin over the lower part of your leg before they bite. In your normal night clothing and with the aid of a flashlight, you will use a sucking tube (with a sieve to prevent the mosquito being sucked into your mouth) to catch mosquitoes that attempt to bite you on your legs and feet.

You will be asked to collect mosquitoes for a number of consecutive nights. Every night, you will work in pairs so that one collector will capture mosquitoes between 6 PM and 12 Midnight and the other one will collect mosquitoes between 12 Midnight and 6 AM (Morning). This mosquito collection will be conducted both outside and inside village/household dwellings where residents have given prior permission for the mosquito collection to take place.

As a mosquito collector you will be offered a course of antimalarial prophylaxis (primaquine) which you can choose to take throughout the collections (starting 2 days before and finishing 2 days after collections cease). Should you fall ill with malaria during the collection, you will receive treatment at no cost. There are no benefits to you for being a mosquito collector and you can choose not to work as a mosquito collector. You are free to stop being a mosquito collector at any time.

You will be paid 20 kina per night (6 hours) for working as a mosquito collector.

CONTACT INFORMATION

____ has described to you what is going to be done, the risks, hazards, and benefits involved, and can be contacted at ________________________. If you have any questions you can call or contact the study coordinator. Dr William Pomat, PNG IMR Director, or his representative can be contacted about individual rights as a research subject. If you are able to get to a telephone, you may call the Institute of Medical Research (422-2909) in PNG. There are no toll free telephone numbers in Papua New Guinea.

B2 Signature Sheet for Mosquito Collectors

Project title: **Community Prevalence Surveys to Monitor Lymphatic Filariasis Indicators Following Treatment with 3-drug Therapy in Papua New Guinea**

Study PIs: **Dr Moses Laman, Dr Leanne Robinson, Dr Christopher L. King, Dr Livingstone Tavul, Dr Stephan Karl**

SIGNATURE FORM

Signing below indicates that you have been informed about this study in which you voluntarily agree for your participation; that you have received the study information brochure; that you have asked any questions about the study that you may have; and that the information given to you has permitted you to make a fully informed and free decision about your willingness to collect mosquitos for this study. By signing this consent form, you do not lose any rights, and the investigators are not relieved of any obligations they may have. A copy of this consent form will be provided to you. If you do not know how to write, then an independent witness to the consent process will be asked to also sign on your behalf.

____ Date ____________________

Printed Name of Mosquito Collector

____

Signature of Mosquito Collector

(If unable to read and/or write, the signature of an independent witness to the consent must be obtained for the consent to be valid)

____ Date ____________________

Printed Name of Witness

____

Signature of Witness

This consent was obtained by:

____

Printed Name of Person Obtaining Consent

____

Signature of Person Obtaining Consent

____

Signature of Study Coordinator

B3 Toksave pepa bilong ol lain husait i wanbel long kisim natnats

Project title: **Community Prevalence Surveys to Monitor Lymphatic Filariasis Indicators Following Treatment with 3-drug Therapy in Papua New Guinea**

Study PIs: **Dr Moses Laman, Dr Leanne Robinson, Dr Christopher L. King, Dr Livingstone Tavul, Dr Stephan Karl**

Dispela em wanpela wokpainimaut. Ol lain husait i wanbel long stap insait long dispela wokpainimaut em ol tasol bai stap insait long dispela wokpainimaut. Yu mas tingting na skelim gut pastaim long yu wanbel long stap insait long dispela wokpainimaut. Toktok wantaim femili na frens bilong yu.

As tingting bilong dispela wokpainimaut em long luksave long namba bilong natnat na sik malaria wea natnat save karim insait long ples bilong yu. Ol save man/meri husait i wokim dispela wokpainimaut laik painimaut tu wonem kain ol natnat tru i stap insait long ples bilong yu.

Mipela askim yu long halipim mipela long kisim natnat long wonem krismas bilong yu antap long 18 yia, yu save stap insait long dispela ples, na kisim pinis skul long pasin bilong kisim natnat bilong dispela wokpainimaut.

Sapos yu wanbel long wok bilong kisim natnat, yu mas save olsem yu gat sans long kisim malaria na ol narapela sik wea natnat save karim. Sans bilong yu long kisim sik malaria bai wankain or antap liklik tasol long ol lain insait long ples husait no wok long kisim natnat. Mipela bai givim yu marasin bilong malaria pastaim long yu stat kisim natnat long pasim yu long kisim malaria. Mipela bai givim yu dispela marasin insait long taim yu wok long kisim natnat na 7-14 deis behain long taim yu kisim natnat.

Sans bilong natnat long kaikai yu taim yu wok long kisim natnat i wankain or antap liklik tasol long taim yu no wok long kisim natnat bilong wonem yu bai stap kirap long nait na kisim natnat wea i sindaun long lek bilong yu. Yu bai kisim natnat sindaun long lek blong yu pastaim long em kisim blut bilong yu. Mipela bai skulim yu long pasin bilong kisim natnat yusim sutlam na wanpela kain gumi.

Mipela bai askim yu long kisim natnat algeta nait long taim mipela kamap long ples bilong yu long kisim natnat. Tupela man bai wok wantaim long taim bilong kisim natnat. Wanpela bai wok long six kilok long apinun inap biknait (twelve kilok long nait). Narapela bai kisim ples bilong em na wok long biknait igo inap six kilok long monin. Yupela bai kisim natnat insait na autsait long haus wea papa bilong haus i givim tok orait pinis long kisim natnat insait long haus bilong em.

Sapos yu tok orait long wok wantaim mipela long kisim natnat, bai mipela givim yu marasin bilong banisim yu long sik malaria. Marasin wei bai yu kisim em ol i kolim primaquine. Bai yu statim marasin 2 pela dei bipo yu statim wok lo kisim natnat na bai yu stop long kisim marasin 1 pla dei behain lo taim u stopim wok wantaim mipela. Mipela bai givim yu marasin bilong sik malaria sapos yu kisim sik malaria long taim yu wok wantaim mipela. Ino gat wanpela gutpela samting bai yu kisim sapos yu wanbel long halipim mipela long kisim natnat. Mipela ino inap pasim yu sapos yu tingting long lusim wok.

Mipela bai givim yu twenti kina (K20) long olgeta nait wea yu wok long kisim natnat.

____ i givim pinis tok klia long wonem samting bai kamap, wonem ol hevi yu ken bungim sapos yu wok long kisim natnat, na wonem gutpela bilong dispela wokpainimaut. Sapos yu gat askim orait yu ken kolim em long telefon namba______________________. Yu can kolim tu direkta bilong PNG IMR, Dr. William Pomat, or ol wokman bilong em sapos yu gat askim long ol raits bilong yu taim yu halipim mipela long kisim natnat. Sapos yu gat telefon, yu ken kolim PNG IMR long namba 422-2909.

B4 Hanmak pepa bilong ol lain husait i wanbel long kisim natnats

Project title: **Community Prevalence Surveys to Monitor Lymphatic Filariasis Indicators Following Treatment with 3-drug Therapy in Papua New Guinea**

Study PIs: **Dr Moses Laman, Dr Leanne Robinson, Dr Christopher L. King, Dr Livingstone Tavul, Dr Stephan Karl**

HANMAK PEPA

Hanmak bilong yu i soim olsem yu kisim gut pinis klia tingting long as tingting bilong dispela wokpainimaut na yu wanbel long wok bilong kisim natnat. Hanmak bilong yu i soim to olsem yu kisim pinis wanpela pepa wea is givim olgeta tok klia long as tingting bilong dispela wokpainimaut na dispela pepa i helpim yu long mekim tingting long halipim mipela long kisim natnat.

Taim yu putim hanmak bilong yu long pepa, dispela ino pasim ol raits bilong yu. Dispela tu ino pasim yu long kotim ol hetman bilong dispela wokpainimaut sapos ol brukim ol lo wea is banisim ol raits bilong yu. Wanpela kopi bilong dispela hanmak pepa bai mipela givim long yu. Sapos yu nogat save long rit na rait orait wanpela witness bai putim hanmak bilong em long nem bilong yu long soim olsem yu wanbel long wok bilong kisim natnat.

____ Dei_______________

Raitim nem bilong yu

____

Hanmak bilong yu *(Sapos yu no save long rit na rait orait mipela bai kisim hanmak bilong witness bilong yu long soim olsem yu wanbel long wok bilong kisim natnat.)*

____________________________________ Dei________________

Nem bilong witness

____

Hanmak bilong witness

Wokman bilong IMR husait kisim hanmak bilong yu:

__________________________ Dei________________

Nem bilong IMR wokman

__________________________ _______________________________

Hanman bilong IMR wokman Hanmak bilong hetman bilong dispela wokpainimaut

APPENDIX C: Questionnaire template for Community Surveys

**Project title:**	**Community Prevalence Surveys to Monitor Lymphatic Filariasis Indicators Following Treatment with 3-drug Therapy in Papua New Guinea**
**Study PIs:**	**Dr Moses Laman, Dr Leanne Robinson, Dr Christopher L. King, Dr Livingstone Tavul, Dr Stephan Karl**
